# The Effects of the Mediterranean Diet on Biomarkers of Vascular Wall Inflammation and Plaque Vulnerability in Subjects with High Risk for Cardiovascular Disease. A Randomized Trial

**DOI:** 10.1371/journal.pone.0100084

**Published:** 2014-06-12

**Authors:** Rosa Casas, Emilio Sacanella, Mireia Urpí-Sardà, Gemma Chiva-Blanch, Emilio Ros, Miguel-Angel Martínez-González, Maria-Isabel Covas, Rosa M Lamuela-Raventos, Jordi Salas-Salvadó, Miquel Fiol, Fernando Arós, Ramon Estruch

**Affiliations:** 1 Department of Internal Medicine, Hospital Clinic, Institut d’Investigació Biomèdica August Pi i Sunyer (IDIBAPS), University of Barcelona, Barcelona, Spain; 2 CIBER 06/03: Fisiopatología de la Obesidad y la Nutrición, Instituto de Salud Carlos III, Madrid, Spain; 3 Nutrition and Food Science Department, Pharmacy Faculty, University of Barcelona, Barcelona, Spain; 4 Lipid Clinic, Service of Endocrinology & Nutrition, Hospital Clinic, IDIBAPS, Barcelona, Spain; 5 Department of Preventive Medicine and Public Health, School of Medicine, University of Navarra, Pamplona, Spain; 6 Cardiovascular Epidemiology Unit, Municipal Institute for Medical Research (IMIM), Barcelona, Spain; 7 Human Nutrition Unit, Hospital Universitari de Sant Joan de Reus, IISPV, Universitat Rovira i Virgili, Reus, Spain; 8 University Institute for Health Sciences Investigation, Palma de Mallorca, Spain; 9 Department of Cardiology, Hospital de Alava, Vitoria, Spain; University of Catanzaro Magna Graecia, Italy

## Abstract

**Background:**

Adherence to the Mediterranean diet (MD) is associated with reduced morbidity and mortality due to cardiovascular disease. However, how the MD exerts its effects is not fully known.

**Aim:**

To assess the 12-month effects of two enhanced MDs compared to a low-fat diet on inflammatory biomarkers related to atherosclerosis and plaque vulnerability in a subcohort of the PREDIMED (Prevención con Dieta Mediterránea) study.

**Methods:**

A total of 164 participants at high risk for cardiovascular disease were randomized into three diet groups: MD supplemented with 50****mL/d of extra virgin olive oil (MD+EVOO) or 30 g/d of nuts (MD+Nuts) and a low-fat diet. Changes in classical cardiovascular risk factors, inflammatory biomarkers of atherosclerosis and plaque vulnerability were measured after 12 months of intervention.

**Results:**

Compared to participants in the low-fat diet group, those receiving MD+EVOO and MD+Nuts showed a higher decrease in systolic (6****mmHg) and diastolic (3****mmHg) blood pressure (P = 0.02; both), as well as a reduction of 10% and 8% in LDL-cholesterol (P = 0.04), respectively. Patients in the MD+Nuts group showed a significant reduction of 34% in CD40 expression on monocyte surface compared to low-fat diet patients (P = 0.03). In addition, inflammatory biomarkers related to plaque instability such as C-reactive protein and interleukin-6 were reduced by 45% and 35% and 95% and 90% in the MD+EVOO and MD+Nuts groups, respectively (P<0.05; all) compared to the low-fat diet group. Likewise, sICAM and P-selectin were also reduced by 50% and 27%, respectively in the MD+EVOO group (P = 0.04) and P-selectin by 19% in MD+Nuts group (P = 0.04) compared to the low-fat diet group.

**Conclusions:**

Adherence to the MD is associated with an increase in serum markers of atheroma plaque stability which may explain, at least in part, the protective role of MD against ischemic heart disease.

**Trial Registration:**

www.controlled-trials.com ISRCTN35739639

## Introduction

Atherosclerosis, the pathological substratum of coronary heart disease (CHD), is a low-grade chronic inflammatory disease of the vascular wall initiated by the accumulation of cholesterol-laden inflammatory cells (monocytes and T-lymphocytes) in the subendothelial space in a self-perpetuating process that leads to the formation of atheroma plaques, the hallmark of the disease [1]. Inflammatory mediators such as adhesion molecules (selectins, integrins) and interleukins (e.g., IL-6, IL-1β, IL-18) participate in this process. In some instances, the atheroma plaque becomes unstable, leading to cap rupture and ensuing thrombosis that occludes the artery and finally, induces cardiovascular events such myocardial infarction or stroke [2]. Some of these inflammatory mediators (e.g., C-reactive protein [CRP], IL-6, intercellular adhesion molecule-1 [ICAM-1], vascular cell adhesion molecule-1 [VCAM-1]) have been considered as useful predictive markers of atherosclerosis [3], whereas other biomarkers (matrix metalloprotease-9 and IL-18) have been associated with plaque vulnerability [4].

The PREDIMED (*Prevención con Dieta Mediterránea*) study is the first randomized clinical trial designed to assess the beneficial effects of the MD on the primary prevention of cardiovascular diseases in elderly subjects at high cardiovascular risk. Up to now the PREDIMED study has demonstrated that adherence to the MD is associated with a reduced incidence of diabetes [5]–[6], the metabolic syndrome [7], hypertension [8], and better control of other cardiovascular risk factors [9]–[10]. In fact, Estruch et al [11] have recently reported that a MD intervention reduces the incidence of cardiovascular events by 30% in subjects at high cardiovascular risk. However, improvement in classical cardiovascular risk factors associated with the MD intervention could not fully explain the protective effect of the MD against CHD [11]. Some authors have suggested that, at least in the short term, MD reduces oxidative stress [12], vascular inflammation [13]–[14], and endothelial dysfunction [15], all of which are related to atheroma plaque formation. Thus, in a previous study we have observed lower serum concentrations of VCAM-1, ICAM-1, E- and P-selectin, CRP and IL-6, as well as a down-regulation of adhesion molecules on T-lymphocyte and monocyte surfaces after 3 months of MD intervention [10], [14], [16]. However, whether this effect is maintained in the long run is unknown. Moreover, little is known about the effect of the MD on markers of plaque vulnerability. Therefore, we embarked on a study to assess 1-y changes in inflammatory biomarkers of atherosclerosis as well as markers of plaque instability in a free-living population with high risk of CHD participating in the PREDIMED study.

## Methods

The protocol for this trial and supporting CONSORT checklist are available as supporting information; see Checklist S1 and Protocol S1.

### Subjects and Design

The PREDIMED study is a parallel-group, multicenter, randomized, controlled 5-year clinical trial aimed to assess the effects of the MD on the primary prevention of cardiovascular disease (CVD) (http://www.predimed.es) [17]. Recruitment took place between October 2003 and January 2009, and the 7447 participants were randomly assigned to one of three interventions (two Mediterranean diets enriched with extra virgin olive oil (EVOO) or mixed nuts, and a control low-fat diet). The design, methodology and eligibility criteria for the PREDIMED study have been previously described [10]–[11], [17]. Briefly, we recruited men aged 55 to 80 years and women aged 60 to 80 years with no previously documented CVD. They were eligible if they had type 2 diabetes, or 3 or more major cardiovascular risk factors (hypertension, high plasma LDL-cholesterol, low plasma HDL-cholesterol, overweight or obesity, current smoking, or a family history of premature coronary heart disease). At baseline and after 12 months of follow-up, the participants filled out a 137-item validated food frequency questionnaire (FFQ), a 14-item questionnaire assessing adherence to the MD and the Minnesota leisure-time physical activity questionnaire. We also recorded medication use, measured anthropometric parameters and blood pressure, and collected fasting blood and a spot urine samples, as described previously [14].

In the current study we screened 193 consecutive potential participants from October 2003 to November 2004 in a primary care center associated with the Hospital Clínic of Barcelona, Spain, but 29 did not fulfill the inclusion criteria. Thus, 164 were finnaly included in this substudy. Main outcome measures were 12-month changes in classical cardiovascular risk factors, and inflammatory markers predictive of atherosclerosis or related to plaque instability.

### Diets

After two screening visits with the dietician, participants who fullilled inclusion criteria signed an informed consent and were randomily assigned in a 1∶1∶1 ratio to one of three dietary interventions (MD+EVOO, MD+Nuts or low-fat diet). Randomization was performed centrally by means of a computer-generated random-number sequence. All of the participants had a face-to-face interview with the dietician and a group session at the baseline visit and quarterly thereafter. The dietary intervention in the three treatment groups was delivered by the same dietician as described [11]. The group sessions were organized separately for each of the three intervention groups. In each session the 14-point score of adherence to the MD was the main tool to change dietary habits for the two MDs, and a similar 9-point score was used in the participants of the low-fat diet group. The focus in the MD groups was to change portion sizes and the frequency of intake of the different foods and to modify cooking methods towards the traditional MD of Mediterranean countries in the sixties. Thus, participants in both MD groups were recommended to increase the intake of vegetables (≥2 servings/d), fresh fruit (≥3 servings/d), legumes, nuts, fish or seafood (≥3 servings/wk), and the use of olive oil for cooking and dressings, as previously described [11]. The focus in the control group was to reduce all types of fat, with particular emphasis on recommending the consumption of lean meats, low-fat dairy products, cereals, potatoes, pasta, rice, fruits and vegetables. All participants were provided with descriptions of seasonal foods, shopping lists, weekly meal plans and cooking recipes, according to the their intervention group. Olive oil and nut industry companies supplied EVOO (50 mL/d) or 30 g/d of walnuts, almonds and halzelnuts free of charge to the respective MD groups, whereas those in the control group received small nonfood gifts. The fatty acid composition of the EVOO and nuts used in the trial is described elsewhere [10]. No total calorie restriction was advised, nor was physical activity promoted. From the beginning of the study all participants were recommended to not use multivitamin or antioxidant supplements.

### Ethics Statement

All participants provided informed consent. Participants had signed the informed Consent. The Institutional Review Board (IRB) of the Hospital Clinic (Barcelona, Spain) accredited by the US Department of Health and Human Services (DHHS) update for Federal wide Assurance for the Protection of Human Subjects for International (Non-US) Institutions #00000738 approved the study protocol on July, 16, 2002. The protocol was also approved by the ethical review board of our hospital.

### Clinical and Laboratory Measurements

Trained personnel measured weight and height using calibrated scales and a wall-mounted stadiometer, respectively; waist circumference was determined midway between the lowest rib and the iliac crest using an anthropometric tape, and blood pressure (BP) was measured in triplicate with a validated semiautomatic oscillometer (Omron HEM-705CP) [10]–[11]. Samples of serum, EDTA-plasma, and urine were coded and stored at −80°C until assay. A technician blinded to group allocation processed peripheral blood mononuclear cells (PBMCs) on the same day of blood extraction. PBMCs were isolated from whole blood by Ficoll-Hypaque (Lymphoprep, Axis-Shield PoC AC) density-gradient. The expression of adhesion molecules on the surface of PBMCs was analyzed via double direct immunofluorescence with the use of commercial monoclonal antibodies following the manufacturer’s instructions. The adhesion molecules analyzed were: anti-CD49d (Cytogmos), anti-CD11a and anti-CD11b (Bender Medsystems), anti-CD40, anti-CD14 and anti-CD2 monoclonal antibodies (Caltag). Cell counts (5000 events for T-lymphocytes and 2000 for monocytes) and fluorescence analysis were performed in a FACSCalibur Flow Cytometer (Becton-Dickinson) using CellQuest software. Results are expressed as mean fluorescence intensity (MFI) in arbitrary units.

ELISAs were performed in baseline and 1-year samples at the end of the study period in thawed plasma with commercial kits for soluble (s) E- and P-selectin, sVCAM-1, sICAM-1, IL-18, IL-6 (BLK and PelkinElmer Elast Amplification System), IL-10 and tissular inhibitor of metalloproteases-1 (TIMP-1) (R&D Systems), MMP-9 (Amersham), and transforming growth factor beta 1 (TGF-β1) (R&D Systems). A technician blinded to group allocation processed the ELISA kits.

Additional serum analytes determined included fasting glucose and immunoreactive insulin, total cholesterol, triglycerides, HDL and LDL-cholesterol, and high-sensitivity CRP, as described elsewhere [10]–[11]. In a random sample of 56 participants (34%) we measured urinary tyrosol and hydroxytyrosol levels (as a measure of adherence to EVOO consumption recommendations) and the plasma α-linolenic acid (ALA) proportion (as a measure of adherence to walnut consumption recommendations), as reported previously [10]. For all laboratory methods, the intra- and inter-assay variation coefficients ranged from 1.8 to 8.9% and from 0.9 to 9.9%, respectively.

### Statistical Analyses

For a parallel design, the sample size was determined with the ENE 3.0 statistical program (GlaxoSmithKline, Brentford, United Kingdom) assuming a maximum loss of 10% of participants. To detect a mean difference of 10 MFI units on the expression of monocyte CD49d with a conservative standard deviation (SD) of 10, 20 subjects would be needed to complete the study (α risk = 0.05, power = 0.9). The monocyte expression of CD49d was considered the primary outcome and used to determine the sample size, but changes in all endpoints were of equal interest in this study.

We used descriptive statistics with the mean ± SD for the baseline characteristics of the participants. Categorical variables are expressed as percentages. Variables with a skewed distribution (Kolmogorov) were transformed to their natural logarithm for analysis. One-factor analysis of variance or chi-square tests, as appropriate, were used to determine differences in baseline characteristics among the three study groups. Changes in all outcomes were assessed with repeated-measures analysis of variance for the two factors, diet and time, and their interactions. Significant interactions were analyzed by the simple effects test with multiple contrasts of Bonferroni. Within- and between-group differences are expressed as means and 95% confidence intervals (CI).

The relationship between monounsaturated acid (MUFA) intake and inflammatory markers was determined by partial correlation analysis. All statistical tests were two-tailed, with significance set at *P*<0.05. Analyses were performed using the SPSS, version 18.0 (SPSS Inc., Chicago, IL).

## Results

### Participant Characteristics

**Figure 1:**
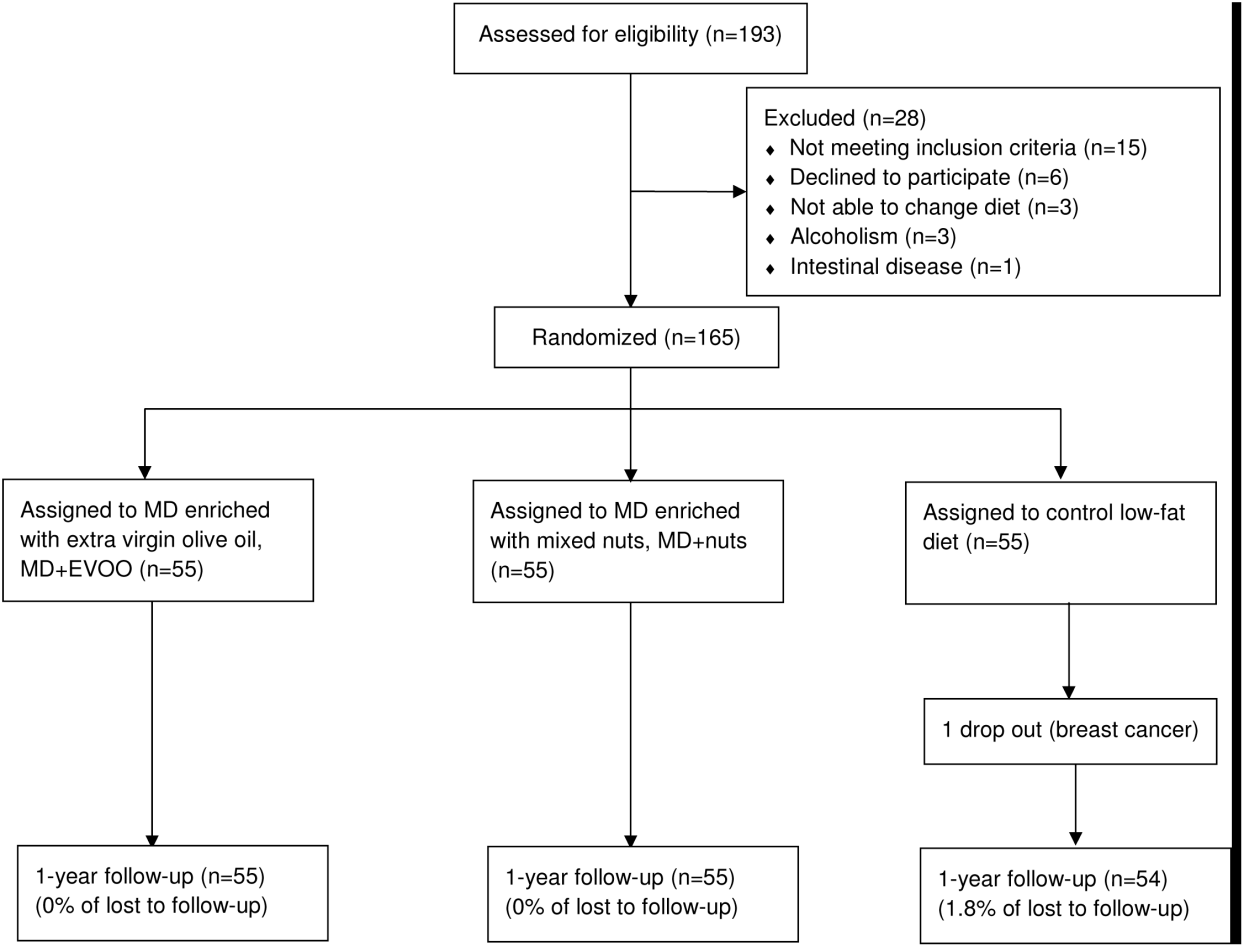
Flowchart of study participants. The diagram includes detailed information on the excluded participants. Abbreviations: MD, Mediterranean diet.

Of the 164 participants finnally included, 55, 55 and 54 were randomized to a MD supplemented with virgin olive oil, a MD supplemented with nuts, and a low-fat control diet, respectively. The retention rates for 1 year follow-up were 100%, 100% and 98.2%, respectively (**Figure 1**). **Table 1** shows the baseline characteristics of these 164 participants, of whom none were lost to follow-up. The groups were well balanced regarding demographic characteristics, adiposity and cardiovascular risk factors. The medication taken and occupation levels were also similar in the three groups. Drug regimens did not appreciably change during the 12-month follow-up.

**Table 1:** Baseline characteristics of the participants.^1^

	MD+EVOO (n = 55)	MD+nuts (n = 55)	Low-fat diet (n = 54)
Age (years)	68.1±6	67.6±6	67.4±6
Men (%)	24 (43.6)	31 (56.4)	22 (40.7)
Family history of CHD (%)	16 (29.1)	9 (16.4)	13 (24.1)
Current smokers (%)	10 (18.2)	11 (20)	11 (20.4)
BMI (kg/m^2^)	27.9±3.4	27.8±3.1	28.5±3.7
BMI≥25 kg/m (%)	47 (85.5)	45 (81.8)	44 (81.5)
Type 2 diabetes (%)	46 (83.6)	44 (80)	37 (68.5)
Hypertension (%)	39 (70.9)	29 (52.7)	37 (68.5)
Dyslipidemia (%)	32 (58.2)	34 (61.8)	38 (70.4)
Medications (%)			
ACE inhibitors	11 (20)	12 (21.8)	13 (24.1)
Diuretics	13 (23.6)	6 (10.9)	14 (25.9)
Other antihypertensive agents	10 (18.2)	8 (14.5)	9 (16.7)
Statins	17 (31)	14 (25.5)	10 (18.5)
Other-lipid-lowering agents	4 (7.3)	2 (3.6)	6 (11.1)
Insulin	3 (5.5)	7 (12.7)	3 (5.6)
Oral hypoglycemic drugs	30 (54.5)	25 (45.5)	29 (53.7)
Aspirin or antiplatelet drugs	10 (18.2)	8 (14.5)	5 (9.3)
NSAIDS	6 (10.9)	10 (18.2)	8 (14.8)

1 Values are mean ± SD or n (%). ACE, angiotensin converting enzyme; BMI, body mass index; CHD, coronary heart disease; EVOO, extra virgin olive oil; MD+EVOO, Mediterranean diet supplemented with extra virgin olive oil; MD+Nuts, Mediterranean diet supplemented with nuts; NSAIDS, Non-steroidal antiinflammatory drugs.

### Cardiovascular Risk Factors

The baseline and 12-month values for the classical cardiovascular risk factors are shown in **Table 2**. The MD+EVOO and MD+Nuts groups showed a mean reduction in systolic BP of 6 mmHg (P = 0.02; both) and in diastolic BP of around 3 mmHg (P = 0.02; both), of 6% and 7% (P = 0.04), respectively, in total-cholesterol, of 10% and 8% (P = 0.04), respectively, in LDL-cholesterol, and of 9% and 5% (P = 0.01), respectively in the cholesterol/HDL-cholesterol ratio. Both MDs showed a decrease in the waist perimeter (P<0.05; both) of the participants from baseline.

**Table 2:** Changes in adiposity, blood pressure and cardiovascular risk factors.

		MD+EVOO (n = 55)		MD+Nuts (n = 55)		Low-fat diet (n = 54)		
		Mean	P^3^	Mean	P^3^	Mean	P^3^	*Pint* 4
Weight (Kg)	Baseline^1^	75.5±1.6		76.9±1.6		75.3±1.7		0.70
	1y.^1^	74.9±1.6		76.7±1.7		75.0±1.7		
	Mean changes^2^	−0.6 (−1.4 to 0.1)	0.09	−0.2 (−1.0 to 0.5)	0.54	−0.3 (−1.1 to 0.5)	0.44	
BMI (Kg/m^2^)	Baseline	29.2±0.5		28.9±0.5		29.3±0.5		0.85
	1y.	29.1±0.5		28.8±0.5		29.2±0.6		
	Mean changes	−0.1 (−0.4 to 0.2)	0.99	−0.1 (−0.4 to 0.2)	0.90	−0.1 (−0.5 to 0.2)	0.44	
Waist circumference (cm)	Baseline	102±1.3		102±1.3		100±1.4		0.09
	1y.	98.6±1.4		99.2±1.4		99.4±1.5		
	Mean changes	−3.2 (−4.6 to −1.7)	<0.001	−2.8 (−4.3 to −1.4)	<0.001	−0.6 (−2.1 to 0.9)	0.42	
Systolic blood pressure (mmHg)	Baseline	152±2.6		148±2.6		153±2.7		0.02
	1y.	146±2.6		141±2.5		155±2.7		
	Mean changes	−6.0 (−10.1 to −2.0)^a^	0.004	−6.4 (−10.5 to −2.4)^a^	0.002	2.2 (−2.1 to 6.5)	0.32	
Diastolic blood pressure (mmHg)	Baseline	85.0±1.3		85.1±1.3		86.8±1.4		0.02
	1y.	81.8±1.2		82.5±1.2		88.4±1.3		
	Mean changes	−3.2 (−5.4 to −0.9)^a^	0.07	−2.6 (−4.9 to −0.4)^a^	0.02	1.6 (−0.8 to 4.0)	0.20	
Glucose (mg/dL)	Baseline	130±5.5		127±5.6		132±5.6		0.83
	1y.	131±5.5		127±5.5		129±5.5		
	Mean changes	1.2 (−6.8 to 9.2)	0.77	−0.2 (−8.3 to 7.9)	0.96	−2.9 (−11.1 to 5.3)	0.49	
Glycated hemoglobin (mg/dL)	Baseline	6.1±0.2		6.0±0.2		6.1±0.3		0.90
	1y.	6.3±0.2		6.1±0.2		6.0±0.2		
	Mean changes	0.2 (−0.1 to 0.5)	0.23	0.1 (−0.2 to 0.3)	0.66	−0.1 (−0.4 to 0.2)	0.45	
Triglycerides (mg/dL)	Baseline	147±11.1		138±11.1		148±11.5		0.80
	1y.	143±8.9		135±8.9		133±9.2		
	Mean changes	−4.2 (−24.0 to 15.5)	0.67	−2.9 (−22.6 to 16.9)	0.77	−15.5 (−36.0 to 5.0)	0.14	
Total-cholesterol (mg/dL)	Baseline	228±4.6		225±4.6		208±4.7		0.04
	1y.	214±4.5		209±4.5		207±4.6		
	Mean changes	−13.5 (−23.0 to −4.1)^a^	0.005	−15.7 (−25.1 to −6.3)^a^	0.001	0.1 (−9.4 to 9.6)	0.98	
HDL-Cholesterol (mg/dL)	Baseline	54.5±1.6		53.8±1.7		55.6±1.7		0.47
	1y.	56.6±1.7		52.6±1.7		53.2±1.8		
	Mean changes	2.1 (−0.2 to 4.4)	0.07	−1.2 (−3.5 to 1.1)	0.31	−2.4 (−2.8 to −2.0)	0.74	
LDL-Cholesterol (mg/dL)	Baseline	145±3.9		144±3.9		128±4.0		0.04
	1y.	130±4.0		132±4.0		124±4.0		
	Mean changes	−14.4 (−21.1 to −7.7)^a^	<0.001	−11.7 (−18.4 to −5.0)^a^	0.001	−3.6 (−10.4 to 3.2)	0.56	
Cholesterol: HDL-Cholesterol ratio	Baseline	4.4±0.1		4.3±0.1		3.8±0.1		0.01
	1y.	4.0±0.1		4.1±0.1		3.9±0.1		
	Mean changes	−0.4 (−0.6 to −0.2)^a^	<0.001	−0.2 (−0.5 to −0.03)^a^	0.03	0.05 (−0.2 to 0.3)	0.83	

Data analyzed by repeated-measures 2-factor ANOVA (simple-effect analysis by Bonferroni’s multiple contrast).

1 Values are mean ± SD.

2 Mean differences (95% CI).

3 P: Significant differences (P<0.05) between before and after the intervention.

4 Pint: comparison between measures obtained before and after intervention and among the 3 diet groups, P<0.05.

a MD+EVOO or MD+nuts vs. low fat-diet are significantly different, P<0.05. EVOO, extra virgin olive oil; MD+EVOO, Mediterranean diet supplemented with extra virgin olive oil; MD+Nuts, Mediterranean diet supplemented with nuts. BMI, body mass index.

### Adhesion Molecule and CD40 Expression in PBMC

As shown in **Table 3**, after 12 months of intervention the MD+EVOO group showed a decrease in CD11a (P<0.001), CD49d (P<0.004) and CD40 (P<0.001) in peripheral T-lymphocytes. In addition, MD+EVOO showed decreased CD11a, CD11b, CD49d and CD40 (P<0.001; all) in circulating monocytes.

**Table 3:** Changes in adhesion molecule expression in circulating T- lymphocytes and monocytes.

		MD+EVOO (n = 55)		MD+Nuts (n = 55)		Low-fat diet (n = 54)		
		Mean	P^3^	Mean	P^3^	Mean	P^3^	*Pint* 4
T-LYMPHOCYTES								
CD11^a^	Baseline^1^	132±4.7		137±5.2		121±5.2		0.26
	1y.^1^	107±5.3		107±6.0		103±6.0		
	Mean changes^2^	−24.4 (−36.0 to −13.0)	<0.001	−29.9 (−43.1 to −16.7)	<0.001	−18.3 (−31.2 to −5.2)	0.006	
CD49d	Baseline	48.3±1.1		39.0±1.1		34.8±1.1		0.33
	1y.	36.7±1.1		41.0±1.1		39.9±1.1		
	Mean changes	−11.7 (−16.1 to −7.2)	0.004	2.0 (−0.7 to 4.7)	0.54	5.1 (3.1 to 7.1)	0.14	
CD40	Baseline	47.5±1.1		55.3±1.1		44.2±1.1		0.20
	1y.	36.7±1.1		42.0±1.1		38.6±1.1		
	Mean changes	−11.0 (−12.6 to −9.3)	0.001	−13.5 (−15.5 to −11.4)	0.001	−5.6 (−6.5 to −4.8)	0.09	
MONOCYTES								
CD11^a^	Baseline	85.0±4.2		80.1±4.4		78.8±4.2		0.41
	1y.	57.3±2.0		56.8±2.1		53.9±2.1		
	Mean changes	−27.7 (−36.1 to −19.5)	<0.001	−23.3 (−32.0 to −14.6)	<0.001	−24.9 (−33.3 to −6.6)	<0.001	
CD11b	Baseline	44.7±2.1		47.3±2.2		43.9±2.2		0.38
	1y.	34.6±1.3		36.5±1.3		35.1±1.3		
	Mean changes	−10.1 (−14.6 to −5.5)	<0.001	−10.8 (−15.6 to −6.1)	<0.001	−8.8 (−13.6 to −4.1)	<0.001	
CD49d	Baseline	35.2±1.1		39.0±1.1		35.0±1.1		0.50
	1y.	27.2±1.1		29.2±1.1		30.7±1.1		
	Mean changes	−8.0 (−9.4 to −6.5)	<0.001	−9.8 (−11.6 to −8.1)	<0.001	−4.3 (−5.3 to −3.2)	0.06	
CD40	Baseline	36.1±2.9		46.5±2.9		35.2±2.9		0.03
	1y.	26.3±1.9		30.9±1.9		31.1±1.9		
	Mean changes	−9.8 (−15.0 to −4.6)^b^	<0.001	−15.6 (−20.9 to −10.2)^a^ ^,b^	<0.001	−4.1 (−9.4 to 1.2)	0.13	

Data analyzed by repeated-measures 2-factor ANOVA (simple-effect analysis by Bonferroni’s multiple contrast).

1 Values are mean ± SD.

2 Mean differences (95% CI).

3 P: Significant differences (P<0.05) between before and after the intervention.

4 Pint: comparison between measures obtained before and after intervention and among the 3 diet groups, P<0.05.

a MD+EVOO or MD+nuts vs. low fat-diet and ^b^MD+EVOO vs. MD+nuts are significantly different, P<0.05. EVOO, extra virgin olive oil; MD+EVOO, Mediterranean diet supplemented with extra virgin olive oil; MD+Nuts, Mediterranean diet supplemented with nuts.

On the other hand, the MD+Nuts group showed a significant decrease in CD11a and CD40 of (P<0.00; all) in T- lymphocytes. For monocytes, the MD+Nuts group showed a significant decrease in CD11a, CD11b, CD49d and CD40 (P<0.001; all).

Finally, the low-fat diet only showed a significant decrease in CD11a in T-lymphocytes and a decrease in CD11a and CD11b expression in circulating monocytes (P<0.001; all).

### Circulating Markers of Plaque Instability and other Inflammatory Biomarkers

Baseline plasma concentrations of inflammatory biomarkers were similar among groups. As can be observed in **Table 4**, after a 12-month intervention the participants allocated to the MD+EVOO showed a decrease in sVCAM-1 (P<0.02), sICAM-1 (P<0.001) and sP-selectin (P<0.001) concentrations. Furthermore, the MD+Nuts group showed a decrease in sVCAM-1 (P<0.001) and sE-selectin (P<0.002) and sP-selectin of (P<0.007). By contrast, the serum concentration of sICAM-1 was significantly increased (P<0.02) in the control group.

**Table 4:** Changes in the expression of circulating markers of plaque instability and other inflammatory biomarkers.

		MD+EVOO (n = 55)		MD+Nuts (n = 55)		Low-fat diet (n = 54)		
		Mean	P^3^	Mean	P^3^	Mean	P^3^	*Pint* 4
sVCAM (ng/mL)	Baseline^1^	872±47.0		935±49.2		776±48.6		0.30
	1y.^1^	734±44.9		727±47.1		720±46.5		
	Mean changes^2^	−138 (−251 to −25.2)	0.02	−208 (−327 to −89.6)	0.001	−55.6 (−173 to 61.5)	0.35	
sICAM (ng/mL)	Baseline	437±27.3		394±23.3		369±24.0		0.04
	1y.	217±22.0		364±18.8		431±19.2		
	Mean changes	−220 (−273 to −166)^b^	<0.001	−30.3 (−76.1 to 15.5)^b^	0.20	62.3 (15.5 to 109)^b^	0.01	
sE-SEL (ng/mL)	Baseline	28.6±2.5		33.0±2.6		32.3±2.6		0.55
	1y.	26.9±2.4		28.3±2.5		30.1±2.5		
	Mean changes	−1.7 (−4.5 to 1.2)	0.26	−4.7 (−7.7 to −1.7)	0.003	−2.2 (−5.3 to 0.9)	0.16	
sP-SEL (ng/mL)	Baseline	91.4±9.3		87.6±9.4		50.0±10.5		0.04
	1y.	66.5±8.3		70.8±8.4		51.1±9.3		
	Mean changes	−25.0 (−32.3 to −17.6)^a^	<0.001	−16.8 (−24.3 to −9.4)^a^	<0.001	1.1 (−7.1 to 9.4)	0.78	
IL-6 (pg/mL)	Baseline	0.7±0.1		0.9±0.1		0.7±0.1		0.04
	1y.	0.4±0.1		0.5±0.1		1.0±0.1		
	Mean changes	−0.3 (−0.9 to 0.3)^a^	<0.001	−0.4 (−1.0 to 0.2)^a^	<0.001	0.3 (−1.1 to 1.7)	<0.001	
CRP (mg/mL)	Baseline	3.8±1.1		3.5±1.1		3.6±1.1		0.04
	1y.	1.9±1.1		2.1±1.1		3.3±1.1		
	Mean changes	−1.9 (−2.4 to −1.6)^a^	<0.001	−1.4 (−2.1 to −0.7)^a^	<0.001	−0.3 (−1.3 to 0.8)	0.46	
IL-18 (pg/mL)	Baseline	139±14.3		131±14.5		103±14.6		0.18
	1y.	137±13.1		112±13.2		101±13.4		
	Mean changes	−1.8 (−13.8 to 10.2)	0.76	−18.6 (6.4 to 30.7)	0.003	−1.3 (−13.5 to 11.0)	0.84	
IL-10 (pg/mL)	Baseline	1.4±1.1		1.3±1.1		1.2±1.1		0.40
	1y.	1.5±1.1		1.4±1.1		1.3±1.1		
	Mean changes	0.05 (−0.2 to 0.3)	0.62	0.05 (−0.2 to 0.3)	0.60	0.1 (−0.1 to 0.3)	0.29	
IL-18/IL-10 ratio	Baseline	31.9±4.0		17.0±4.1		20.6±4.0		0.02
	1y.	17.2±3.4		7.9±3.5		19.0±3.4		
	Mean changes	−14.7 (−23.1 to −6.2)	0.001	−9.1 (−18.0 to −0.3)	0.04	−1.6 (−10.1 to 6.9)	0.71	
MMP-9 (ng/mL)	Baseline	7.7±1.2		7.9±1.2		6.2±1.2		0.78
	1y.	10.0±1.2		10.4±1.2		10.5±1.2		
	Mean changes	2.3 (0.9 to 3.8)	0.13	2.5 (1.1 to 3.8)	0.11	4.3 (1.2 to 7.3)	0.003	
TIMP-1 (ng/mL)	Baseline	143±6.7		146±7.3		144±7.2		0.94
	1y.	146±7.4		144±8.2		152±8.2		
	Mean changes	2.7 (−8.7 to 14.0)	0.64	−2.4 (−14.9 to 10.1)	0.71	7.5 (−4.7 to 19.8)	0.23	
MMP-9/TIMP-1 ratio	Baseline	0.06±1.2		0.06±1.2		0.04±1.2		0.60
	1y.	0.08±1.2		0.08±1.2		0.07±1.2		
	Mean changes	0.02 (0.01 to 0.03)	0.125	0.02 (0.01 to 0.03)	0.15	0.03 (0.01 to 0.06)	0.03	
TGF-β1 (pg/mL)	Baseline	40.2±2.3		46.7±2.4		43.0±2.5		0.11
	1y.	44.5±2.1		49.3±2.2		49.0±2.3		
	Mean changes	4.3 (−0.4 to 9.0)	0.08	2.6 (−2.2 to 7.5)	0.30	5.9 (0.9 to 11.0)	0.02	

Data analyzed by repeated-measures 2-factor ANOVA (simple-effect analysis by Bonferroni’s multiple contrast).

1 Values are mean ± SD.

2 Mean differences (95% CI).

3 P: Significant differences (P<0.05) between before and after the intervention.

4 Pint: comparison between measures obtained before and after intervention and among the 3 diet groups, P<0.05.

a MD+EVOO or MD+nuts vs. low fat-diet are significantly different, P<0.05.

b All the groups differed, P<0.05. EVOO, extra virgin olive oil; MD+EVOO, Mediterranean diet supplemented with extra virgin olive oil; MD+Nuts, Mediterranean diet supplemented with nuts.

Other molecules related to plaque instability **(** **Table 4** **)**, such as CRP and IL-6 (P<0.001; both) and the IL-18/IL-10 ratio (P≤0.04) decreased in the MD+EVOO and MD+nuts groups. Furthermore, in the MD+nuts group, IL-18 concentration also decreased (P<0.003). Finally, the control group showed a significant increase in IL-6 (P<0.001), MMP-9 (P<0.003) and TGF-β1 (P<0.02) levels. The same group, showed an increase, albeit no significant, in the MMP-9/TIMP-1 ratio (P<0.003) and TIMP-1, although the changes were not significant.

None of the groups showed significant Pearson correlation coefficients between MUFA intake and any of the inflammatory marker concentrations.

### Changes in Food and Nutrient Intake during Follow-up

The self-reported dietary habits of the participants prior to starting the study were similar among the three groups. Baseline diets were high in fiber, total fat and MUFA because of a high baseline consumption of olive oil and marine n3 fatty acids due to frequent fish intake **(Appendix S1 and S2)**. The saturated fatty acid (SFA) and ω3-polyunsaturated fatty acid (PUFA) content of the diets was relatively low.

Dietary intervention resulted in favorable changes in food consumption, mainly in the MD groups **(Appendix S1)**. Accordimgly, after 12 months these subjects showed a significant increase in adherence to the MD pattern (P<0.001).

**Appendix S2** shows changes in baseline energy and nutrient intake after the 12-month intervention. At 12 months, the participants in the MD+EVOO group increased the average urine concentration of the phenolic compound tyrosol 8.0 ng/mL (P<0.02) from a baseline value of 61.0 ng/mL, and hydroxytyrosol increased 39.0 ng/mL (P<0.04) from a baseline value of 205 ng/mL, while the plasma content of ALA increased 0.14 mol% (P<0.01) from a baseline value of 0.3 mol% in subjects assigned to the MD+Nuts group. These three parameters were used as an objective measure of compliance in the MD intervention diet groups.

## Discussion

Higher adherence to a MD intervention supplemented with EVOO or nuts, for at least 12 months, were associated with a significant decrease in inflammatory markers related to atheroma plaque formation and plaque instability, in addition to a reduction in BP and plasma LDL-cholesterol concentration [18]–[19]. All of these mechanisms may explain, at least in part, the lower incidence of CHD, stroke and mortality in subjects at high risk of CVD following a Mediterranean diet as has been recently demonstrated by the PREDIMED study [11].

In the long term, a decrease of 10 or 5 mmHg in systolic or diastolic BP, respectively, is associated with a 40% and 30% reduction in the risk of stroke or myocardial infarction, respectively [20]. Moreover, a 10% reduction in plasma cholesterol concentrations has been associated with a 20% reduction in CHD risk [21].

In the current paper, we observed a significant decrease of 6 mmHg in systolic BP and 3 mmHg in diastolic BP in both MD groups [10]. This same trend was observed in plasma total-cholesterol concentrations, with a decrease of 5% for the MD+EVOO and 8% for the MD+Nuts compared to the control group. These declines were also similar to those already observed at 3 months for both MDs [10]. Nonetheless, improvements in BP and the lipid profile can not explain the whole protective action of the MD against atherosclerosis, thereby suggesting the presence of alternative effects.

It is well known that atherosclerosis is a chronic low-grade inflammatory disease of the arterial wall [2]. Thus, modulation of this inflammatory reaction may be another potential way by which the MD protects against atherosclerosis. In the current study, we detected several antiinflammatory effects in the three diets studied, although they were more intense in subjects allocated to the two MD interventions. These subjects showed a higher down-regulation of adhesion molecules in T-lymphocytes and monocytes compared to those in the control group. Moreover, serum concentrations of endothelial soluble cell adhesion molecules, CRP and IL-6 were also lower in subjects following the two MDs compared to control subjects. These results agree with those previously published by Castaner et al [28] and Estruch et al [10], both based in data from PREDIMED trial, in which a sustained traditional MD supplemented with EVOO or nuts may exert health benefits through changes in the transcriptomic response of genes related to cardiovascular risk. Moreover, the IL-18 and IL-18/IL-10 ratio, which are related to ischemic events in the heart and brain [29]–, were decreased after the MD+nuts and both MD interventions, respectively, suggesting a greater stability of atheroma plaque in patients following a MD diet.

This antiinflammatory effect has already been associated with different interventions related to a lower incidence of cardiovascular disease such as a diet rich in fruit, vegetables and olive oil [22], statins [23], moderate alcohol intake [24] or physical activity [25]. In fact, previous studies have reported that some components of the MD such as EVOO or nuts may down-regulate inflammatory markers related to atherosclerosis such as VCAM-1, ICAM-1, E- and P-Selectin, CRP and IL-6 [26]–[27].

When we analyzed the possible mechanisms responsible for the antiinflammatory effect observed in both MD groups, the role of exercise on immunomodulation is probably residual since no changes in physical activity were observed in the three groups. In fact, we only observed a significant increase in physical activity in the low-fat diet group compared to both MD groups, but paradoxically this situation was not associated with a greater antiinflammatory effect as would be expected in this group [25]. Finally, the antiinflammatory effect may be due to a synergistic action among nutrients from key foods of the MD such as EVOO or nuts.

Interestingly, the antiinflammatory effect of the MD seems to be greater and more intense in the mid-term compared to the short-term [10], [14], while the effect on classical cardiovascular risk factors was similar, thereby suggesting that the MD exerts its effects on lipids and blood pressure relatively quickly (at 3 mo), with the maximum effect on systemic inflammatory biomarkers being achieved later (at 1 y). Thus, in the short-term the effect on BP and the lipid profile is higher, whereas in the mid-term the effect on chronic inflammatory response in the arterial wall is more pronounced.

The strengths of our study are its design as a randomized controlled clinical trial, excellent completion rates, good compliance, and the specific inflammatory biomarkers studied, which are involved in different phases of atheroma plaque formation. Finally, there is the question as to the clinical relevance of the antiinflammatory effects of MD. However, the recently published results of PREDIMED [11] showing lower cardiovascular morbility and mortality are sufficiently consistent to reject any doubt about the antiinflammatory effect of the MD. Regarding the limitations, this study was performed in subjects at high cardiovascular risk and therefore, the results may not be generalized to the overall population. Moreover, this study was limited to classical cardiovascular risk factors and inflammatory parameters. Thus, we can not exclude other protective effects on other clinical or biological parameters related to cardiovascular risk such as markers of arterial structure and function or oxidative stress.

In summary, the results of our study suggest that the Mediterranean diet supplemented with EVOO or nuts has a dual effect on the prevention of cardiovascular disease improving classical cardiovascular risk factors and also has an intense antiinflammatory effect. Both of these effects could partially explain the overall beneficial effect of the MD on the primary prevention of cardiovascular disease observed in high risk subjects.

## Supporting Information

Appendix S1 **Consumption of key food items, physical ctivity and 14-point Mediterranean diet score.**
(PDF)

Appendix S2 **Changes in baseline energy and nutrient intake.**
(PDF)

Protocol S1 **Predimed Study: Mediterranean diet in the primary prevention of cardiovascular disease.**
(PDF)

Checklist S1 **CONSORT checklist.**
(DOC)
