# Supplementary material for: The Effects of the Mediterranean Diet on Biomarkers of Vascular Wall Inflammation and Plaque Vulnerability in Subjects with High Risk for Cardiovascular Disease. A Randomized Trial
Source: PLoS One. 2014 Jun 12;9(6):e100084. doi: 10.1371/journal.pone.0100084 (PMC4055759; doi:10.1371/journal.pone.0100084)
Supplement: Appendix S1 — Consumption of key food items, physical ctivity and 14-point Mediterranean diet score. (PDF) [file pone.0100084.s001.pdf]

Table S1

Consumption of key food items, physical activity and 14-point Mediterranean diet score.

		MD + EVOO (n=55)		MD + Nuts (n=55)		Low-fat diet (n=54)		
		Mean	P[3]	Mean	P[3]	Mean	P[3]	*Pint*[4]
EVOO (g/d)	Baseline[1]	12.1 ± 2.0		11.6 ± 2.0		9.0 ± 2.1		<0.001
	1y.[1]	52.5 ± 2.0		10.9 ± 2.0		11.0 ± 2.0		
	Mean changes[2]	40.4 (35.9 to 45.0)[a,b]	<0.001	-0.7 (-5.3 to 3.8)[b]	0.76	2.0 (-3.5 to 7.1)	0.34	
Refined OO (g/d)	Baseline	20.5 ± 2.2		19.2 ± 2.2		25.0 ± 2.3		<0.001
	1y.	0.3 ± 1.9		26.9 ± 1.9		23.0 ± 2.0		
	Mean changes	-20.2 (-25.0 to -15.5)[a,b]	<0.001	7.7 (2.9 to 12.6)[b]	0.002	-2.0 (-7.0 to 3.0)	0.43	
Total nuts (g/d)	Baseline	14.6 ± 2.4		17.9 ± 2.4		13.5 ± 2.4		<0.001
	1y.	11.8 ± 2.2		55.9 ± 2.2[a,b]		13.8 ± 2.1		
	Mean changes	-2.8 (-7.6 to 1.8)[b]	0.44	38.0 (32.5 to 43.5)[a,b]	<0.001	0.3 (-5.1 to 5.6)	0.91	
Vegetables (g/d)	Baseline	401 ± 19.2		329 ± 19.3		355 ± 20.5		0.003
	1y.	483 ± 19.2		400 ± 19.2		363 ± 19.2		
	Mean changes	82.6 (33.4 to 132)[a]	0.001	71.5 (22.0 to 121)	0.005	8.3 (-44.4 to 60.9)	0.76	
Legumes (g/d)	Baseline	18.1 ± 1.2		18.0 ± 1.2		19.6 ± 1.3		0.17
	1y.	24.6 ± 1.5		26.3 ± 1.5		18.8 ± 1.6		
	Mean changes	6.5 (3.1 to 9.8)[a]	<0.001	8.3 (5.0 to 11.7)[a]	<0.001	-0.8 (-4.3 to 2.7)	0.66	
Fruits (g/d)	Baseline	455 ± 28.8		466 ± 26.3		398 ± 30.5		0.11
	1y.	567 ± 26.1		530 ± 28.8		486 ± 27.6		
	Mean changes	112 (56.6 to 168)	<0.001	64.8 (8.8 to 121)	0.02	88.0 (29.2 to 147)	0.004	
Cereals (g/d)	Baseline	299 ± 16.4		278 ± 16.5		271 ± 17.4		0.92
	1y.	233 ± 14.0		244 ± 14.1		245 ± 14.8		
	Mean changes	-66.0 (-95.9 to -36.3)	<0.001	-33.8 (-63.9 to -3.7)	0.03	-25.5 (-57.1 to 6.1)	0.11	
Fish or seafood (g/d)	Baseline	101 ± 5.6		116 ± 5.8		104 ± 6.0		0.22
	1y.	114 ± 5.7		117 ± 5.8		102 ± 6.0		
	Mean changes	12.7 (3.1 to 22.2)	0.10	0.4 (-9.2 to 10.1)	0.93	-1.4 (-11.4 to 8.7)	0.79	
Meat or meat products (g/d)	Baseline	156 ± 7.8		163 ± 7.9		155 ± 8.4		0.57
	1y.	146 ± 7.4		147 ± 7.5		134 ± 8.0		
	Mean changes	-10.0 (-24.7 to 4.7)	0.18	-16.0 (-31.1 to -1.0)	0.04	-21.2 (-37.0 to -5.4)	0.01	

Pastries, cakes or sweets (g/d)	Baseline	15.2 ± 2.4		15.8 ± 2.5		14.1 ± 2.6		0.71
	1y.	10.5 ± 2.0		12.7 ± 2.0		15.8 ± 2.0		
	Mean changes	-4.7 (-9.4 to 0.02)	0.04	-3.1 (-7.9 to 1.7)	0.21	1.7 (-3.3 to 6.7)	0.50	
Dairy products (g/d)	Baseline	387 ± 27.8		342 ± 28.3		375 ± 29.5		0.31
	1y.	355 ± 27.4		308 ± 27.9		369 ± 29.1		
	Mean changes	-32.1 (-88.0 to 23.7)	0.26	-33.7 (-90.6 to 23.2)	0.25	-6.2 (-64.7 to 52.5)	0.84	
Alcohol (g/d)	Baseline	11.1 ± 2.7		13.1 ± 2.7		10.3 ± 2.9		0.58
	1y.	9.3 ± 2.0		12.3 ± 2.7		9.4 ± 2.1		
	Mean changes	1.8 (-6.2 to 2.7)	0.44	-0.8 (-5.3 to 3.7)	0.72	-0.9 (-5.6 to 3.7)	0.69	
Wine (mL/d)	Baseline	70.3 ± 12.2		48.2 ± 12.5		58.9 ± 12.7		0.85
	1y.	66.7 ± 12.3		65.4 ± 12.6		62.2 ± 12.8		
	Mean changes	-3.6 (-21.8 to 14.6)	0.70	17.2 (-1.5 to 36.0)	0.07	3.3 (-14.8 to 21.8)	0.70	
Physical Activity (kcal/d)	Baseline	346 ± 30.5		286 ± 28.5		209 ± 28.8		0.01
	1y.	383 ± 37.6		306 ± 35.1		271 ± 35.6		
	Mean changes	36.7 (-34.5 to 108)[a]	0.31	19.6 (-47.3 to 87.5)[a]	0.56	61.9 (-5.5 to 129)	0.07	
MD Score	Baseline	8.3 ± 0.2		7.9 ± 0.2		8.5 ± 0.3		0.04
	1y.	9.6 ± 0.2		9.8 ± 0.2		8.1 ± 0.2		
	Mean changes	1.3 (-0.1 to 2.7)[a]	<0.001	1.9 (1.3 to 2.3)[a]	<0.001	-0.4 (-0.8 to 0.2)	0.15	

Data analyzed by repeated-measures 2-factor ANOVA (simple-effect analysis by Bonferroni's multiple contrast).[1]Values are mean ± SD. [2]Mean differences (95% CI). [3]P: Significant differences (P<0.05) between before and after the intervention. [4]Pint: comparison between measures obtained before and after intervention and among the 3 diet groups. [a]MD+EVOO or MD+nuts vs. low fat-diet and [b]MD+EVOO vs. MD+nuts are significantly different, P<0.05. EVOO, extra virgin olive oil; MD+EVOO, Mediterranean diet supplemented with extra virgin olive oil; MD+Nuts, Mediterranean diet supplemented with nuts; Refined OO, refined olive oil.
