# Supplementary material for: The Effects of the Mediterranean Diet on Biomarkers of Vascular Wall Inflammation and Plaque Vulnerability in Subjects with High Risk for Cardiovascular Disease. A Randomized Trial
Source: PLoS One. 2014 Jun 12;9(6):e100084. doi: 10.1371/journal.pone.0100084 (PMC4055759; doi:10.1371/journal.pone.0100084)
Supplement: Appendix S2 — Changes in baseline energy and nutrient intake. (PDF) [file pone.0100084.s002.pdf]

Table S2

Changes in baseline energy and nutrient intake.

		MD + EVOO (n=55)		MD + Nuts (n=55)		Low-fat diet (n=54)		
		Mean	P[3]	Mean	P[3]	Mean	P[3]	*Pint*[4]
Energy (kcal/d)	Baseline[1]	2461 ± 87.9		2561 ± 87.0		2338 ± 87.0		0.02
	1y.[1]	2465 ± 75.0		2641 ± 74.3		2248 ± 74.3		
	Mean changes[2]	3.5 (-129 to 136)[c]	0.96	79.7 (-51.0 to 211)[c]	0.23	-89.6 (-220 to 400)[c]	0.18	
Protein (% E)	Baseline	16.9 ± 0.4		16.8 ± 0.4		17.5 ± 0.4		0.34
	1y.	16.5 ± 0.3		16.0 ± 0.3		16.7 ± 0.3		
	Mean changes	-0.4 (-1.0 to 0.2)	0.18	-0.8 (-1.4 to -0.1)	0.02	-0.8 (-1.5 to -0.2)	0.007	
Carbohydrate (% E)	Baseline	43.3 ± 1.0		41.7 ± 1.0		43.1 ± 1.1		0.01
	1y.	39.7 ± 0.8		37.2 ± 0.8		42.7 ± 0.8		
	Mean changes	-3.6 (-5.4 to -1.7)	<0.001	-4.5 (-6.2 to -2.5)[a]	<0.001	-0.4 (-2.4 to 1.6)	0.69	
Fiber (g/d)	Baseline	29.1 ± 1.2		29.4 ± 1.2		26.6 ± 1.3		0.01
	1y.	31.4 ± 1.1		32.4 ± 1.2		27.1 ± 1.2		
	Mean changes	2.3 (-0.1 to 4.8)[a]	0.04	3.0 (0.5 to 5.5)[a]	0.02	0.5 (-2.1 to 3.1)	0.69	
Total fat (% E)	Baseline	36.8 ± 0.9		38.1 ± 0.9		36.3 ± 0.9		0.001
	1y.	41.0 ± 0.7		43.5 ± 0.8		37.4 ± 0.8		
	Mean changes	4.2 (2.4 to 6.0)[a]	<0.001	5.4 (3.6 to 7.3)[a]	<0.001	1.1 (-0.8 to 3.0)	0.24	
SFA (% E)	Baseline	10.1 ± 0.3		10.6 ± 0.3		9.7 ± 0.3		0.13
	1y.	10.0 ± 0.3		10.5 ± 0.3		9.8 ± 0.3		
	Mean changes	-0.1 (-0.8 to 0.5)	0.64	-0.1 (-0.8 to 0.5)	0.68	0.1 (-0.5 to 0.7)	0.99	
MUFA (% E)	Baseline	17.4 ± 0.5		17.6 ± 0.5		17.4 ± 0.5		0.01
	1y.	21.6 ± 0.5		20.5 ± 0.5		18.0 ± 0.5		
	Mean changes	4.2 (3.1 to 5.4)[a]	<0.001	2.9 (1.8 to 4.1)[a]	<0.001	0.6 (-0.6 to 1.8)	0.32	
ω3 PUFA (% E)	Baseline	6.3 ± 0.3		6.7 ± 0.3		6.0 ± 0.3		<0.001
	1y.	6.1 ± 0.2		9.4 ± 0.2		6.2 ± 0.2		
	Mean changes	-0.2 (-0.8 to 0.4)	0.60	2.7 (2.1 to 3.3)[a]	<0.001	0.2 (-0.5 to 0.8)	0.57	
α- linolenic acid (g/d)	Baseline	1.7 ± 0.1		2.0 ± 0.1		1.6 ± 0.1		<0.001
	1y.	1.5 ± 0.1		3.0 ± 0.1		1.5 ± 0.1		
	Mean changes	-0.2 (-0.5 to 0.03)	0.09	1.0 (0.7 to 1.2)[a]	<0.001	-0.1 (-0.3 to 0.2)	0.87	

Marine n3 fatty acids (g/d)	Baseline	0.7 ± 0.06		0.9 ± 0.06		0.7 ± 0.06		0.28
	1y.	0.9 ± 0.06		0.8 ± 0.06		0.6 ± 0.06		
	Mean changes	0.2 (0.1 to 0.3)	0.002	-0.05 (-0.2 to 0.1)	0.36	-0.05 (-0.2 to 0.1)	0.31	
Cholesterol (mg/d)	Baseline	393 ± 15.0		427 ± 15.1		390 ± 15.8		0.05
	1y.	376 ± 16.8		412 ± 17.0		355 ± 17.8		
	Mean changes	-17.4 (-48.5 to 13.6)	0.27	-14.6 (-45.9 to 16.8)	0.36	-34.8 (-66.7 to -2.6)	0.04	
β-Carotenoids (g/d)	Baseline	4.7 ± 0.4		4.0 ± 0.4		3.4 ± 0.4		0.06
	1y.	5.2 ± 0.5		4.3 ± 0.5		3.1 ± 0.5		
	Mean changes	0.5 (-0.5 to 1.5)[a]	0.31	0.3 (-0.7 to 1.3)	0.56	-0.3 (-1.3 to 0.8)	0.60	
Folic acid (mg/d)	Baseline	473 ± 15.3		461 ± 15.3		445 ± 16.1		0.01
	1y.	510 ± 15.4		496 ± 15.4		422 ± 16.1		
	Mean changes	37.0 (6.7 to 67.7)[a]	0.02	35.0 (4.0 to 65.0)[a]	0.03	-23.0 (-54.5 to 9.4)	0.17	
Vitamin B6 (mg/d)	Baseline	2.7 ± 0.07		2.6 ± 0.07		2.5 ±0.08		0.02
	1y.	2.9 ± 0.08		2.8 ± 0.08		2.4 ± 0.09		
	Mean changes	0.2 (0.02 to 0.3)[a]	0.03	0.2 (0.04 to 0.3)[a]	0.01	-0.1 (-0.2 to 0.06)	0.22	
Vitamin B12 (mg/d)	Baseline	10.1 ± 0.6		11.3 ± 0.6		9.8 ± 0.6		0.10
	1y.	11.7 ± 0.7		10.7 ± 0.7		9.2 ± 0.7		
	Mean changes	1.6 (0.4 to 2.8)	0.01	-0.6 (-1.8 to 0.6)	0.31	-0.6 (-2.0 to 0.6)	0.64	
Vitamin C (mg/d)	Baseline	249 ± 12.0		220 ± 12.0		219 ± 12.6		0.005
	1y.	283 ± 12.6		237 ± 12.6		220 ± 13.3		
		34.0 (7.9 to 60.3)[a,b]	0.01	17.0 (-8.8 to 43.6)[b]	0.20	0.5 (-27.0 to 28.0)	0.97	
Vitamin D (mg/d)	Baseline	5.5 ± 0.5		6.6 ± 0.5		6.0 ± 0.5		0.46
	1y.	6.9 ± 0.5		6.4 ± 0.5		5.7 ± 0.5		
	Mean changes	1.4 (0.6 to 2.2)	0.001	-0.2 (-1 to 0.6)	0.58	-0.2 (-1.1 to 0.6)	0.57	
Vitamin E (mg/d)	Baseline	10.3 ± 0.4		11.6 ± 0.4		9.5 ± 0.4		0.01
	1y.	11.3 ± 0.5		10.2 ± 0.5		9.3 ± 0.5		
	Mean changes	0.9 (0.02 to 1.9)[c]	0.04	-1.4 (-2.3 to -0.4)[c]	0.004	-0.2 (-1.2 to 0.8)[c]	0.69	
Non-heme iron (mg/d)	Baseline	15.0 ± 0.5		14.5 ± 0.5		13.8 ± 0.6		0.007
	1y.	16.0 ± 0.5		15.7 ± 0.5		13.2 ± 0.5		
	Mean changes	1.1 (0.03 to 2.2)[a]	0.04	1.2 (0.1 to 2.3)[a]	0.03	-0.6 (-1.7 to 0.6)	0.34	
Heme iron (mg/d)	Baseline	4.4 ± 0.2		4.7 ± 0.2		4.3 ± 0.2		0.26
	1y.	4.1 ± 0.2		4.1 ± 0.2		3.9 ± 0.2		
	Mean changes	-0.3 (-0.6 to 0.04)	0.03	-0.6 (-0.9 to -0.3)	<0.001	-0.4 (-0.8 to -0.1)	0.006	

Data analyzed by repeated-measures 2-factor ANOVA (simple-effect analysis by Bonferroni's multiple contrast).[1]Values are mean ± SD. [2]Mean differences (95% CI). [3]P: Significant differences (P<0.05) between before and after the intervention. [4]Pint: comparison between measures obtained before and after intervention and among the 3 diet groups. [a]MD+EVOO or MD+nuts vs. low fat-diet and [b]MD+EVOO vs. MD+nuts are significantly different, P<0.05. [c]All the groups differed, P<0.05. EVOO, extra virgin olive oil; MD+EVOO, Mediterranean diet supplemented with extra virgin olive oil; MD+Nuts, Mediterranean diet supplemented with nuts; MUFA, MonoUnsaturated Fatty Acid; PUFA, PolyUnsaturated Fatty Acid; Refined OO, refined olive oil; SFA, Saturated Fatty Acids.
