# Supplementary material for: The Effects of the Mediterranean Diet on Biomarkers of Vascular Wall Inflammation and Plaque Vulnerability in Subjects with High Risk for Cardiovascular Disease. A Randomized Trial
Source: PLoS One. 2014 Jun 12;9(6):e100084. doi: 10.1371/journal.pone.0100084 (PMC4055759; doi:10.1371/journal.pone.0100084)
Supplement: Protocol S1 — Predimed Study: Mediterranean diet in the primary prevention of cardiovascular disease. (PDF) [file pone.0100084.s003.pdf]

## SUPPLEMENTARY INFORMATION

## Table of Contents


| | Page |
|---|---|
| 1. ORIGINAL PROTOCOL | 2 |
| 2. SUMMARY OF CHANGES | 30 |
| 3. FINAL PROTOCOL | 33 |
| Statistical analysis plan | 55 |

# PREDIMED STUDY.

# MEDITERRANEAN DIET IN THE PRIMARY PREVENTION OF CARDIOVASCULAR DISEASE

**Research Protocol**

**October 2003**

## MEDITERRANEAN DIET IN PRIMARY CARDIOVASCULAR PREVENTION Research Plan

### A. SPECIFIC AIMS

A Mediterranean-type diet (MeDiet) has been widely considered as a model of healthy eating. The adherence to a MeDiet was associated with lower coronary mortality in a cohort study (**Trichopoulou, 2003**) and in a relatively small trial with patients after a myocardial infarction (**De Lorgeril, 1999**), but no large primary prevention trial with clinical events as end-point has been performed. Furthermore, there are discrepancies among nutrition experts because of the high-fat content of MeDiets (up to >40% of total energy intake), which is in conflict with the usual recommendation to follow a low-fat diet in order to avoid overweight/obesity and to prevent coronary heart disease (CHD) (**Ferro-Luzzi, 2003; Jequier, 2002; USDA, 2000; Connor, 1997; Bray, 1998**). The potential cardiovascular preventive effect of MeDiets in the face of the huge and increasing global burden of cardiovascular disease (**Reddy, 2004; WHO, 2003a**) makes the answer to this question a priority for public health. Other important reasons to thoroughly assess the effect of dietary guidelines based on the MeDiet, include: a) the long tradition of following MeDiets without any harm in Southern Europe, where life expectancy is extraordinarily high (**Willett, 1995**), b) the low incidence of CHD in these countries in spite of having similar or even higher levels of classical risk factors compared to the US population (**Tunstall-Pedoe, 1999**); c) the diversity of mechanistic and epidemiological observations of beneficial effects on cardiovascular health of the consumption of key components of the MeDiet, such as monounsaturated fatty acids (MUFA), extra-virgin olive oil (EVOO), or nuts; and d) the higher palatability, acceptance and compliance with MeDiets in comparison with low-fat diets (**McManus, 2001**).

In the era of evidence-based medicine, nutritional recommendations should be based in large scale randomized intervention studies in which clinically relevant ("hard") end-points are evaluated. However, no randomized controlled trial has ever been conducted to assess to what extent a MeDiet offers greater benefits than a usual diet in the primary prevention of cardiovascular events. This is the research plan of the first primary prevention trial, the PREDIMED Study **(PREDIMED** meaning ***PREvención con DIeta MEDiterránea*),** which plan to test the effects of a MeDiet intervention on prevention of cardiovascular disease. The results of this large scale randomized trial should provide the strong evidence required to support dietary guidelines for the general public. This study needs to be conducted in Spain for two reasons: a) It is more cost-effective to expand an ongoing trial that has developed all its infra-structure than to start a new one; b) the US population is not yet prepared to adopt a MeDiet in a short period of time.

#### A.1 Primary Specific Aims

Specific Aim 1 (**MeDiet+Olive oil**): to test the effect of a MeDiet enriched with supplemental extra-virgin olive oil on a composite endpoint of cardiovascular death, myocardial infarction, coronary revascularization procedures, and stroke.

Specific Aim 2 (**MeDiet+Nuts**): to test the effect of a MeDiet enriched with supplemental walnuts, almonds, and hazelnuts on a composite endpoint of cardiovascular death, myocardial infarction, coronary revascularization procedures, and stroke

**A.2 Other Specific Aims**

We will ascertain changes in blood lipids, blood pressure (BP), markers of inflammation and other intermediate markers of cardiovascular risk to better understand how dietary changes are able to modify the risk of clinical events.

**B. BACKGROUND AND SIGNIFICANCE**

Cardiovascular disease is the main cause of death worldwide at the turn of the XXI century. Western countries, including the US, currently continue to exhibit unacceptable high absolute rates of cardiovascular morbidity and mortality. Furthermore, these diseases constitute emerging and neglected epidemics in developing countries (**WHO, 2003a**, **WHO, 2003b**). Surprisingly, a low incidence of CHD is found in some developed countries such as France, Spain, Greece, Italy, and Portugal, leading to a higher life expectancy as compared with Northern European countries or the US (**WHO, 1993; Tunstall-Pedoe, 1999**). The Mediterranean food pattern has been the factor most frequently invoked to explain this health advantage.

**B.1 The Mediterranean diet (MeDiet)**

The MeDiet is identified as the traditional dietary pattern found in olive-growing areas of Crete, Greece and Southern Italy in the late 1950s and early 1960s. Its major characteristics are: a) a high consumption of non-refined grains, legumes, nuts, fruits and vegetables; b) a relatively high-fat consumption (even greater than 40 percent of total energy intake) mostly from MUFA, which accounts for 20 percent or more of the total energy intake; c) olive oil used to cook and for dressing salads is the principal source of fat; d) fish consumption is moderate to high; e) poultry and dairy products (usually as yogurt or cheese) are consumed in moderate to small amounts; f) a low consumption of red meats, processed meats or meat products; g) a moderate alcohol intake, usually in the form of red wine consumed with meals (**Trichopoulou, 1995**).

Replacing saturated fat with MUFA from olive oil produces a decline in total and LDL cholesterol comparable to that attained by low fat diets, but HDL cholesterol is maintained at higher levels, thus obtaining a net advantage on the overall lipid profile (**Perez-Jimenez, 2002; Willett, 2000**). In addition, LDL particles enriched with oleic acid and with a lesser PUFA content may be less easily converted to modified, proinflammatory LDL particles. High olive oil consumption, the hallmark of MeDiet (**Hu, 2003**), is partially responsible for these effects. Additional mechanisms have been suggested for the beneficial effect of the MUFA-rich MeDiet (**Perez-Jimenez, 2002)**. Among them (see below), it has been reported that incorporation of oleic acid into cultured endothelial cells decreases the expression of endothelial leukocyte adhesion molecules with reductions in VCAM-1 and inhibition of nuclear factor-kappaB activation (**Carluccio, 1999**). Postprandial factor VII is significantly lower after a MUFA-rich diet. Olive oil is also associated with a reduced DNA synthesis in human coronary smooth muscle cells (**Mata, 1997**).

The background of a long and ancient tradition with no evidence of harm makes the MeDiet very promising for public health. The high fruit and vegetable intake adds to its high antioxidant content and other mechanistic benefits provided by the polyphenols present in EVOO and red wine. Wide sectors of the scientific community and of consumers believe in a cardio-protective role for MeDiets. This hypothesis fits well into the current paradigm of studying **overall dietary patterns** instead of simply assessing isolated nutrients in

nutritional epidemiology. The rationale is that food items and nutrients may have synergistic or antagonistic effects when they are consumed in combination. Additionally, overall patterns better represent the dietary practices found in free-living populations and therefore provide more useful epidemiological information (**Jacques, 2001; Jacobs, 2003**). Consequently, they have a higher potential for acceptability, palatability and future compliance when they are recommended in behavior counseling. Therefore, in spite of its relatively high fat content, the theoretical advantages of the MeDiet are multiple. To increase vegetable consumption, the fat-free or low-fat dressings are less acceptable than the use of olive oil or other full-fat salad dressings. The sautéing or stir frying of vegetables with variable amounts of olive oil instead of using low-fat spreads or steaming increases taste and results in long-term maintenance of a vegetable-rich diet. These preparation and cooking techniques are typical of MeDiet, where the custom is to cook vegetables in olive oil to enhance flavor. Hence, in health promotion and nutritional education a better compliance with MeDiet will be expected. In fact, a recent trial of weight loss (**McManus, 2001**) reported a better adherence to a MeDiet than to a low-fat diet. Participants viewed this diet as more tasty than low-fat regimens, and this may have been the cause of their increased long-term compliance.

However, at present, there is evidence of an undesirable departure from traditional MeDiets in Southern European countries, especially among younger people (**Sanchez-Villegas, 2003; Costacou, 2003**), with increasing consumption of red meats, processed meats, and foods and beverages high in simple sugars. In addition, the traditional consumption of EVOO is being replaced by other refined vegetable oils of inferior quality. The presumable antiatherogenic properties of olive oil have been mainly attributed to its high content in MUFA oleic acid. However, in recent years converging evidence indicates that polyphenols only present in EVOO significantly contribute to these benefits. The concentration of phytochemicals in oils is influenced by the oil extraction procedures. EVOO is obtained from the first pressing of the ripe fruit and has a high content in antioxidants (tocopherols, polyphenols, flavonoids) and sterols. Lower quality olive oils (refined olive oils, ROO) lose nearly all of this antioxidant capacity because they are refined by physical and chemical procedures, they are mostly coloreless and aroma free, although their fatty acid composition is close to that of EVOO **(Ramirez-Tortosa, 1999; Lercker, 2000).** EVOO polyphenolic derivatives (hydroxityrosol, tyrosol and their secoiridoid derivatives) have shown strong antioxidant and anti-inflammatory activity *in vitro* (**De la Puerta, 1999; 2001**; **Manna, 2002; Visioli, 2002).** In randomized cross-over trials that compared EVOO with ROO (in doses similar to the usual MeDiet), EVOO increased plasma antioxidant capacity and decreased serum tromboxane $B_2$ production**.** It also increased LDL resistance to oxidation**.** EVOO affords better protection than dietary alpha-tocopherol against lipid peroxidation **(Mataix, 1998)** and shows a more comprehensive array of effects related to atherosclerosis reduction, such as reducing oxidative stress **(Visioli, 1998).** Consequently, ROOs are thought not to provide such high cardiovascular benefits as EVOO and It is important to distinguish between each type of oil when analyzing the effects of olive oil on cardiovascular risk. EVOO has been shown to induce a dramatic regression of atherosclerosis in a hamster model (**Mangiapane, 1999**). In a randomized trial including 22 hypercholesterolemic men, EVOO improved endothelial function (**Fuentes, 2001**), and in another trial with 23 hypertensive patients, it significantly reduced BP and the need for antihypertensive medications (**Ferrara, 2000)**. In any case,

we assume that the effect of the overall MeDiet pattern will be more important than the isolated action of a single component.

## B.2 Available evidence about MeDiet and cardiovascular prevention

A MeDiet was inversely associated with mortality from all-causes in several small observational cohort studies of elderly people (**Trichopoulou, 1995; Kouris-Blazos, 1999; Lasheras, 2000**). Recent findings from the Greek EPIC cohort, with more than 22,000 participants, suggested that a higher adherence to the MeDiet is associated with a reduction in total mortality and, more specifically, in coronary mortality (**Trichopoulou, 2003**). Two recent case-control studies also found an inverse association between adherence to the MeDiet and incidence of non-fatal coronary events (**Martinez-Gonzalez, 2002; Panagiotakos, 2002**). Two secondary prevention trials found a remarkable reduction in reinfarction or death when coronary patients were assigned to a so-called "Mediterranean diet" (**De Lorgeril, 1999; Singh, 2002**). The first of these trials, the Lyon Diet Heart Study (**De Lorgeril, 1999**), found a 50%-70% relative reduction in the risk of mortality or reinfarction when patients who had suffered a myocardial infarction were assigned to an experimental diet rich in bread, vegetables, fish, and fruit and low in red meat (replaced with poultry). Butter and cream were replaced with a special margarine rich in alpha-linolenic acid (ALA). The other trial (**Singh, 2002**) found a 52% relative reduction in total cardiac endpoints with an experimental diet rich in fruits, vegetables, nuts, whole grains and mustard seed or soy bean oil. The 50% to 70% observed reduction of cardiac events in the experimental group of the Lyon trial after 46 months leads us to think that if these results were generalized to non-Mediterranean populations, substantially enhanced and efficient methods to reduce CHD would be available. It would be shortsighted not to acknowledge the vast public health benefit that a MeDiet could provide with adoption by the healthy population-at-large if the findings of the secondary trials are also confirmed in primary prevention trials. The American Heart Association (AHA) has given attention to MeDiets as potentially useful for the prevention of CHD, but a cautious recommendation has been issued highlighting that more studies are needed before advising people to follow a MeDiet. These studies will disentangle whether the diet itself or other factors (such as more physical activity, a beneficial genetic background or stronger social support systems) account for the lower incidence of CHD in the Mediterranean countries (**Kris-Etherton, 2001a**).

## B.3 Limitations of published studies on MeDiet and cardiovascular prevention

The two published trials using so-called “MeDiets” have included patients who had already experienced a clinical event, i.e. they were **secondary** prevention trials (**De Lorgeril, 1999; Singh, 2002**), and their results may not be extrapolated to issue dietary guidelines for primary prevention. Additionally, major aspects of the design and methods of these two trials have been overtly criticized. Both trials were designed to provide the experimental group with a high amount of ALA. However, no special consideration was given to olive oil, which is the major source of dietary fat in Mediterranean countries or to the special relevance of EVOO as an important source of polyphenols. On the other hand, no protective effect for olive oil was observed in the Greek EPIC cohort (**Trichopoulou, 2003**), thus raising the issue of whether the large amount of fat provided by olive oil is in fact affording protection against CHD (**Hu, 2003**). In the Lyon Diet Heart Study, the

assessment of the true dietary patterns and nutrient intakes at baseline and at end of the study were reported for only a subset of participants (30% of the control group and 50% of the experimental group). In addition, no biochemical verification of dietary compliance was done. Thus, the diet followed by most participants completing the trial is not known (**Kris-Etherton, 2001a; Robertson, 2001**). A third major issue was that only 12.9 percent of energy intake was provided by MUFA in the group assigned to "MeDiet" in the Lyon Trial. In the Indo-Mediterranean Study, the percentage of MUFA intake was even lower (10% of total energy) and the diet of the intervention group (26% fat) could be actually interpreted as a low-fat diet. These values for fat and MUFA intake are far below the higher MUFA content (>=20%) of the traditional MeDiet (**Perez-Jimenez, 2002**). This is contrary to what should be expected with a MeDiet intervention and these two trials have actually applied interventions not fully in line with the traditional MeDiet. Additional concerns about the Lyon trial are related to the low number of observed end-points (44/14, in the control and intervention group respectively), the improbably large reduction in relative risk (RR) (in spite of not observing changes in some of the classical risk factors), and to the fact that the trial was stopped very early (after 27 months of follow-up)**. No available randomized trial has assessed an EVOO-rich MeDiet in primary cardiovascular prevention. The PREDIMED study will be the first large trial** to randomize high-risk patients to follow-up a **traditional MeDiet for primary cardiovascular prevention.** The PREDIMED trial is designed to overcome the previous limitations and to provide results with the best quality of evidence.

**B.4 Nuts and cardiovascular prevention**

Walnuts, almonds, hazelnuts, and other nuts, like pine nuts, are common staples in the traditional MeDiet, since they are locally produced throughout the Mediterranean basin. Nuts are very high in fat (73-90% of the total energy they provide, 48-63 g/100 g edible portion). Most nuts are rich in MUFA (mostly, oleic acid), whereas walnuts are high in PUFAs (linoleic and ALA). The dietary fiber content in nuts is high, ranging from 5 to 9% by weight. Nuts are good sources of arginine, potassium, vitamin E and other bioactive compounds, such as flavonoids, other polyphenols, and phytosterols. Several small randomized trials (<50 subjects) showed consistent decreases in total cholesterol and LDL-cholesterol after interventions with diets high in a variety of nuts, walnuts or almonds (**Kris-Etherton, 2001b**). These results were achieved with intakes that would amount to two or three servings per day. Effects on HDL-cholesterol were inconsistent. When evaluated, the ratios of total cholesterol to HDL cholesterol decreased (**Feldman EB, 2002**). These mechanistic results suggest that frequent nut consumption may decrease the risk of CHD.

A large Californian cohort, the Adventist Health Study, observed that the frequency of nut consumption was inversely associated with cardiovascular risk (**Fraser, 1992**). More recently, three other large cohorts, the Iowa Women's Health Study, the Nurses Health Study and the Physician's Health Study, have consistently shown that frequent nut consumption is associated with a lower CHD risk (**Ellsworth, 2001; Hu, 1998; Albert, 2002**). However, the results of the Iowa cohort did not reach statistical significance and the Physician’s Health Study cohort only found protection for sudden cardiac death, but not for non-sudden coronary death or nonfatal myocardial infarction.

**No available randomized trial has used nuts in primary cardiovascular prevention. The PREDIMED study will be the first large trial** to randomize high-risk patients to receive nuts for cardiovascular prevention.

## B.5 High-fat diets based on MUFA in diabetics, overweight subjects and high risk individuals

Traditionally, nutrition advice in diabetics, obese subjects, and those with cardiovascular risk factors emphasized avoiding animal fat and, preferably, all kinds of dietary fat, and replacing them with carbohydrate (CHO). The rationale was that fats provided excess energy, thought to promote obesity. However, scientific evidence has accumulated in the last two decades about the beneficial role of diets with a relatively high MUFA content on cardiovascular risk factors, obesity and diabetes. These beneficial MUFA are provided by the MeDiet and, specifically, by olive oil and most nuts (**Ros, 2003; Sabaté, 2003; Garcia-Lorda, 2003)**. In fact, the frequent intake of simple CHO in many otherwise low-fat foods is associated with weight gain. However, when nutrition advice is given to people with obesity or diabetes, a reluctance still exists to recommend high-fat, high-MUFA diets as an alternative to the traditional (and less palatable) low-fat diets. By the design of the trial, a sizeable number of the PREDIMED participants are either overweight/obese, or type 2 diabetics. It is thus important to recognize that there is no evidence that a higher percentage of fat in the diet in the form of MUFA results in increased body weight. The lack of a fattening effect of such MUFA-rich diets has been shown in the context of controlled diets **(Ros, 2003)**, weight-reduction programs **(McManus, 2001)**, and *ad libitum* diets **(Sabaté, 2003)**. Furthermore, the results of our pilot study (see below) are also sobering with respect to the lack of weight gain in the intervention groups.

## B.6. Significance of primary prevention randomized trials

Some contradictory results of observational studies and subsequent large randomized trials have raised major concerns. These conflicting results reinforce the need to obtain the highest available level of evidence before considering any global public health strategy to promote the MeDiet as a model of healthy eating. This evidence is only obtained by conducting large scale randomized trials with clinical cardiovascular events as the principal outcome. Dietary guidelines can be safely issued when consistency is found between observational and experimental studies.

No randomized controlled trial has ever been conducted to assess the MeDiet (or a MeDiet supplemented with nuts) in the primary prevention of cardiovascular disease. While there is a wide consensus that the best answer to the above mentioned questions ideally should be addressed by large randomized trials with “hard” end-points (i.e., clinical events)**,** the difficulties and cost of such trials have precluded their implementation and have led to the belief that they are unfeasible. However, secondary prevention trials using food patterns as main interventions have been conducted with more than 600 participants (**De Lorgeril, 1999**) or even with 1000 participants (**Singh, 2002**). We believe that a primary prevention trial is also feasible because we have already recruited (as of July 2004) 2,000 high-risk but disease-free participants for the PREDIMED trial and found evidence after a 3-month follow-up that our behavioral intervention does modify the participants’ dietary pattern and cardiovascular risk profile in the expected direction. An experimental design in

the Mediterranean population conducting an intervention only on diet and not on other lifestyle factors will provide the answer to the question formulated by the AHA about the specific protection of the MeDiet on CHD.

## C. PRELIMINARY STUDIES

### C. 1 Preliminary trials of olive oil and nuts

Among other relevant published trials, we highlight two very recent trials performed by members of our research team.

#### C. 1.1 Phenolic content in dietary EVOO decreases in vivo LDL oxidation

A randomized double blinded, crossover feeding trial using three similar olive oils with increasing phenolic concentration was conducted in 30 healthy volunteers. Olive oils were administered over three periods of 3 weeks preceded by two-week washout periods. In vivo plasma oxidized LDL exhibited a 34 % reduction (p=0.01) with increasing phenolic content. In vitro resistance of LDL to oxidation also increased from 114 to 122 min (p=0.006). These results supported the hypothesis that EVOO consumption prevents LDL oxidation and could thus reduce the risk of CHD (**Marrugat, in press)**.

#### C. 1.2 A walnut diet improves endothelial function in hypercholesterolemic subjects

In a crossover design, 21 hypercholesterolemic men and women were randomized to a cholesterol-lowering MeDiet and a diet of similar energy and fat content in which walnuts replaced about 32% of the energy from MUFA. Participants followed each diet for 4 weeks. The walnut diet improved endothelium-dependent vasodilatation and reduced levels of VCAM-1 (p<0.05 for both). The walnut diet significantly reduced total and LDL cholesterol (p<0.05 for both). A cardioprotective effect of nut intake beyond cholesterol lowering was shown suggesting that a walnut-enriched MeDiet may provide even further benefits for cardiovascular prevention **(Ros, in press)**.

### C. 2 Preliminary case-control study of MeDiet and the risk of myocardial infarction

Using a case-control design (171 cases of myocardial infarction and 171 age-, gender- and hospital-matched controls) we assessed the protection against CHD afforded by a MeDiet. Six food items were considered protective: 1) olive oil, 2) fiber, 3) fruits, 4) vegetables, 5) fish and 6) alcohol. A positive score of 1 to 5 corresponding to the quintile of intake of each of these items was assigned to each participant, while a negative score was assigned for consumption of food groups assumed to be harmful: 7) meat/meat products, and 8) some carbohydrate-rich items with high glycemic index (white bread and other items). The 8 quintile values were summed up for each participant to build a MeDiet score. The higher was the MeDiet score, the lower the odds ratio (OR) of myocardial infarction. A significant linear trend was apparent after adjustment for the main cardiovascular risk factors. For each additional point in the score (observed range: 9–38) the OR (95% confidence interval, CI) was 0.92 (0.86–0.98). Our data supported the hypothesis that increasing compliance with a MeDiet can be an effective approach for reducing the risk of CHD (**Martinez-Gonzalez, 2002**).

## D. DESIGN AND METHODS

**D.1 Summary**

The proposed study is a parallel group, multi-center, randomized, single-blinded trial aimed at assessing the effects on the risk of major cardiovascular events of two intensive behavioral counseling and nutrition education interventions in comparison with a control group. Both intervention groups are assigned a traditional Mediterranean diet (MeDiet). In one of these two groups we are supplementing the traditional MeDiet with extra-virgin olive oil, providing 1 l/wk for free to participants, and in the second group we are supplementing the MeDiet with nuts and they receive a free supply of 30 g/d of nuts (15 g walnuts, 7.5 g hazelnuts, and 7.5 g almonds). The third arm of randomization is the control diet group, whose participants does not receive education on the MeDiet, but are given advice on how to follow a low-fat diet. We are recruiting men (age: 55 to 80 years) and women (age: 60 to 80 years) with either type-2 diabetes or three or more major cardiovascular risk factors (smoking, high blood pressure (HBP), high LDL cholesterol, low HDL cholesterol, overweight or obesity, and family history of premature CHD). All participants are free of cardiovascular disease at baseline. Study participants are randomized to three equally sized groups (3,500 in each of the three groups). They will be followed-up for clinical outcomes during a median time of four years by the primary care physicians who recruited them for the study. The primary endpoint will be a composite outcome of cardiovascular events, including any of the following: cardiovascular death, myocardial infarction, coronary revascularization procedures, and stroke.

**D. 2 Timeline, progress and need of additional funding for an expanded follow-up.**

The Spanish Ministry of Health has funded the current project for the period 2003-2005. The planning of the project took place after receiving the Spanish grant in March 2003 (PI: Ramon Estruch, Funding agency: Fondo de Investigaciones Sanitarias, FIS, Instituto de Salud Carlos III, G03/140). A thematic network of 16 Spanish research groups was then established.

D. 2.1 Planning

Our network hired, trained and certified the needed personnel (a dietitian and a nurse for each center) in all FC. From March to September 2003 we developed the logistics, protocols, operations’ manual, instruments, forms, and data entry/management systems. Each FC contacted approximately 20 Primary Health Care Centers to recruit participants. During this same period, menus and buying lists of Mediterranean products to be provided to participants were developed. Recruitment planning was also completed in eight of the eleven FC. From April to September 2003, dietitians and nurses were trained and certified in nutritional education and procedures for extraction and storage of blood and other biological samples, respectively.

D. 2.2. Implementation

After obtaining reassuring data confirming adequate changes in diet and risk factors during the pilot phase, the recruitment of participants has progressed and is currently ongoing at a good rate. The expertise of Primary Care physicians in clinical trials is enough because most of them had participated previously in other trials. Participants will be recruited in cohorts. Each FC will recruit a cohort of 900 participants, distributed in approximately 20 Primary Care Centers (PCC) for each FC (with an average of 53

participants recruited per PCC). In each PCC, 5 to 10 Primary Care physicians are recruiting and following participants. Our experience with other studies is that 5 new patients can be recruited daily in each FC. Taking into account holiday periods and failures of invited participants to attend the scheduled visits, we estimate that each FC can recruit 90 new participants per month, with this inclusion rate the targeted sample size of 9,000 participants will be reached in June 2005.

D 2.3 Follow-up

Participants recruited in late 2003 will be followed for up to 5+ years and will have the longest follow-up. But participants entering the study after December 2004 will be followed only for less than 4 years, thus having the shortest follow-up. Consequently, we expect a median follow-up of 4 years.

| | 2003 2004 2005 2006 2007 2008 2009 |
|---|---|
| Recruitment | Oct-------------------------Jun |
| Longest Follow-up | Oct---------------------------------------------------------------------------Dec (>5 yr) |
| Shortest Follow-up | Jun--------------------------------------------Dec (3.5 yr) |

D. 2.4 Analysis/closeout

Clinical closeout will take place by December 31, 2008. During January to March 2009, the ascertainment of outcomes, laboratory measurements, data entry, and respond-to-data edits will be completed, and data will be prepared for statistical analysis. Statistical analyses and writing of the reports for scientific publications will be done during the rest of the year 2009 and part of 2010.

**D. 3 Study population / eligibility criteria**

Trial participants will consist of 9,000 community-dwelling high-risk persons, with ages 55 to 80 years for men and 60 to 80 years for women. They should be free of cardiovascular disease and meet at least one of the two following criteria: **a)** or **b)** in D. 3 .1.

D. 3.1 Inclusion criteria: either **a)** or **b)** should be met.

**a) Type-2 diabetes**. Diagnosis of type-2 diabetes is based on at least one of the following criteria:

-Current treatment with insulin or oral hypoglycemic drugs.

-Fasting glucose > 126 mg/dl (fasting is defined as no caloric intake at least for 8 hours).

-Casual glucose > 200 mg/dl with polyuria, polydipsia, or unexplained weight loss.

-Glucose > 200 mg/dl in two measurements after an oral glucose tolerance test

**OR**

**b) Three or more** of the following **risk factors**:-current smoker (>1 cig/day during the last month)

-HBP (systolic BP>=140 or diastolic BP>=90 mmHg or under antihypertensive medication)

-LDL-cholesterol >= 160 mg/dl

-HDL-cholesterol <= 40 mg/dl independently of lipid-lowering therapy
-body mass index >=25 kg/$m^2$
-family history of premature CHD (definite myocardial infarction or sudden death before 55 years in father or male 1st-degree relative, or before 65 years in mother or female 1st-degree relative)
-If the HDL-cholesterol level is >=60 mg/dL, one risk factor should be subtracted.

D. 3.2 Exclusion criteria. Major exclusion criteria are:

- Documented history of previous cardiovascular disease, including CHD (angina, myocardial infarction, coronary revascularization procedures or existence of abnormal Q waves in the electrocardiogram (EKG)), stroke (either ischemic or hemorrhagic, including transient ischemic attacks), and clinical peripheral artery disease with symptoms of intermittent claudication.
- Severe medical condition that may impair the ability of the person to participate in a nutrition intervention study (e.g. digestive disease with fat intolerance, advanced malignancy, or major neurological, psychiatric or endocrine disease)
- Any other medical condition thought to limit survival to less than 1 year.
- Immunodeficiency or HIV-positive status.
- Illegal drug use or chronic alcoholism or total daily alcohol intake >80 g/d.
- Body mass index > 40 kg/$m^2$.
- Difficulties or major inconvenience to change dietary habits
- Impossibility to follow a Mediterranean-type diet, for religious reasons or due to the presence of disorders of chewing or swallowing (e.g., difficulties to consume nuts)
- A low predicted likelihood to change dietary habits according to the Prochaska and DiClemente stages of change model (**Nigg, 1999**).
- History of food allergy with hypersensitivity to any of the components of olive oil or nuts.
- Participation in any drug trial or use of any investigational drug within the last year.
- Institutionalized patients for chronic care, those who lack autonomy, are unable to walk, lack a stable address, or are unable to attend visits in the PCC every 3 months.
- Illiteracy.
- Patients with an acute infection or inflammation (e.g., pneumonia) are allowed to participate in the study 3 months after the resolution of their condition.

**D. 4 Recruitment**

Most FC have considerable experience and a successful track record of recruiting participants for both epidemiological studies and clinical trials. In addition, the participation of Primary Care Physicians will ensure a high enrollment rate. Because these physicians are responsible for the usual medical care of potential participants and they are aware of their risk factors, no potential ethical conflict regarding confidentiality exists at the stage of identification of suitable participants for the trial. This process starts with extracting their names from the PCC records. Most PCC (over 70 %) participating in the study have computer-based records of patients, making the selection relatively simple. Our experience during the pilot study indicates that the easiest way to select high-risk subjects is to obtain a complete database of persons with either diabetes or HBP fulfilling also the eligible gender-specific age ranges. When computer-based records are not available, Primary Care physicians develop a list of suitable candidates. The clinical records of these persons

are then individually reviewed to exclude those who do not meet eligibility criteria. Candidates are contacted by telephone and are invited to attend a visit in the PCC, where the purpose and characteristics of the study are explained and an informed consent is requested (see below). Alternatively, the Primary Care physician may personally invite candidates to participate on the occasion of scheduled visits for any medical reason. A brief general explanation of the study, including the possibility that they may receive free allowances of olive oil or nuts for the duration of the trial is given in this first visit. Our experience has been that >70 % of candidates approached in this way agree to return for the screening visit.

**D. 5 Participants' visits**

Participants' eligibility for the trial is determined by review of their clinical record and by two formal screening visits. In these visits, questionnaires are filled in, a clinical history is taken, anthropometric and BP measurements are made, and fasting blood for biochemical analyses is obtained. Information collected in the screening visits will also provide baseline data for subsequent analyses of the effects of the interventions on intermediary biomarkers and risk factors.

- **Pre-Screening Evaluation**: After review of clinical records, eligible candidates are contacted by telephone to know if they are both capable and willing to participate in the study. Those giving a positive response are scheduled for the first visit. Data on participation proportion are collected.

- **Screening visit 1**: The visit, performed by the Primary Care physician, serves to identify inclusion/exclusion criteria in a more comprehensive manner. This 15-30 minute visit includes:

**a)** A face-to-face administration of a 26-item questionnaire to inquire about the medical conditions and risk factors related to eligibility, including assessment of the willingness to make diet changes (Prochaska model). **b)** A review of the last EKG if available in the clinical record. If no EKG has been performed within the last year, an EKG is done during this visit. **c)** If the candidate meets all the requirements (including EKG data), an informed consent form is given to him/her for signing after detailed explanation of all procedures and of the anticipated time commitment. The informed consent comprises two parts, one for trial participation and biochemical analyses and another for DNA collection for genetic analyses of stored samples. **d)** The following forms and questionnaires are provided to be read and completed at home:

-a detailed written explanation of the study

-a food frequency questionnaire (FFQ) with 137 items plus vitamin/minerals supplements (adapted from the Willett questionnaire and validated in Spain, see **Martin-Moreno, 1993**), in addition to specific questions about patterns of alcohol consumption.

-The Minnesota physical activity questionnaire (validated Spanish version, **Elosua, 1994 & 2000**).

**e)** The participant is instructed to collect toe-nail specimens and bring them in the next visit.

**f)** The next visit (screening visit 2) is scheduled and the candidate is told to attend it after an overnight fast for blood extraction.

After the first screening visit, the participant is randomized to one of three diet groups.

- **Randomization**: The study nurse randomly assigns each participant to the corresponding intervention group following tables of random allocation according to the recruitment order in blocks of 50 participants, balanced by sex and age group (< 70 years and ≥ 70 years). These tables have been centrally elaborated by the Coordinating Unit and provide a stratified random sequence of allocation for each FC using closed envelopes. The four strata for stratified randomization are built according to gender and age (cut-off point: 70 years). The Primary Care physicians do not participate in the process of randomization. The study nurses are independent of the nurse staff of the PCC. Therefore, they are not involved in the usual clinical care of participants, their first and exclusive role being to collect the data for the PREDIMED trial.

- **Screening visit 2**: This 1 hour visit includes the following:

**a)** A simplified assessment of adherence to the MeDiet (14-item questionnaire). **b)** In a face-to-face interview with the candidate, the dietitian explains again in detail the purpose and development of the study. **c)** The dietitian reviews (and completes with the candidate if needed) the FFQ and physical activity questionnaires. Alternatively, she helps the candidate who had difficulties at home to fill in the questionnaires during the visit. **d)** The nurse measures the weight, height, waist circumference, and BP, and performs a measurement of the ankle-brachial blood pressure index. **e)** The nurse performs a venipuncture, obtains and handles blood samples, and proceeds to prepare the specified serum, plasma, and buffy-coat aliquots. **f)** A urine sample and toe nail specimens are collected by the nurse. **g)** A 47-item general questionnaire collecting information about current medication and risk factors is filled-in. Information to fill this questionnaire is also abstracted from clinical records by the research nurse.

## D.6 Intervention

The PREDIMED dietitians are directly responsible for the dietary intervention. All PREDIMED dietitians are registered dietitians, trained and certified to deliver the PREDIMED intervention protocol. Before the implementation of the protocol, training consisted of approximately 24 hours of initial theoretical and practical group discussion with experts in nutrition education and discussion in between 3 to 5 conference calls to review and improve the protocol. These calls are continued bimonthly throughout the study. During the calls each dietitian discusses her practice sessions with the team, and together the group identifies problems and solutions in protocol implementation. Feedback and discussion also occur among the dietitians and the center coordinators, and between center coordinators and the Data Manager, especially after data from FFQ and objective biochemical measurements (in a random sample of 10% participants) of compliance are analyzed.

D. 6. 1 Control diet group: After screening visit 2, participants randomized to the Control diet group have an interview with a PREDIMED dietitian. This interview includes:
**a)** A simplified assessment of adherence to the MeDiet (14-item questionnaire). **b)** Brief personal recommendations to follow a low-fat diet (AHA guidelines). For total fat intake these recommendations are in some way opposite to those given to the participants in the 2 MeDiet intervention groups). **c)** A leaflet with written recommendations to follow a low-fat diet is given). No further visits are scheduled for the participants allocated to this group until the 1-year follow-up evaluation.

D. 6. 2 Intervention groups

D. 6.2.1 Individual motivational interview with a PREDIMED dietitian
After screening visit 2, participants randomized to either of the two MeDiet intervention groups have a face-to-face interview with the dietitian. This interview includes:
**a)** The 14-item questionnaire of adherence to the MeDiet (See Appendix).
**b)** Personal individual recommendations for changes to be introduced in the participant's diet in order to achieve a personalized goal. The dietitian provides a comprehensive number of reasons to adopt a MeDiet, highlighting the advantages of following this diet, rather than the risks of not adhering to it, and transmitting a positive message with stress on the particular benefits for diabetic patients and for those at high cardiovascular risk. Our previous experience with diabetic patients using this approach for a behavioral intervention to quit smoking in Primary Care has been successful (**Canga, 2000**). We also have experience in using the stages of change model for dietary change (**Lopez-Azpiazu, 2000**). The dietitian personalizes the message by adapting it to the patient's clinical condition, preferences, and beliefs. The training of the PREDIMED dietitians emphasized the holistic approach to lifestyle change in order to tailor the intervention to nutritional assessment and individual needs, encourage adherence to the MeDiet, transmit a sense of empowerment, and, importantly, feel a self-reward for each upward step in the 14-point MedDiet score. A contracting procedure is used and a negotiated change in diet is the targeted goal, working with the subject to determine what he or she considers an attainable goal. The focus can be shifted from changing portion sizes to changing frequency of intake or to changes in cooking methods. Accomplishments in the previous months, even if minor, are considered as support to provide further empowerment and self-reward. The usefulness and effectiveness of this approach has been shown in an even larger randomized trial currently conducted in the US that is aimed instead to reduce fat intake (**Mossavar-Rahmani, in press; Patterson, 2003**). Importantly, caution is taken to make sure that participants with diabetes, overweight/obesity, and/or hyperlipidemia may not receive contradictory dietary advice from other health professionals external to the PREDIMED trial. The PCC physicians, who are responsible for the health care of participants, are aware of this caution. Because unsaturated fats like those contained in olive oil and nuts are still perceived as fattening by some nutrition experts, it is particularly important to allay the fear of an eventual weight gain that might have both the person who is on a weight-management program and his/her nutritionist. This is done by tactful exposition of recent scientific evidences (**McManus, 2001; Ros, 2003; Sabaté, 2003; Garcia-Lorda, 2003**) and, on from June 2004, by explaining that body weight did not change after 3 months of MeDiet intervention in the pilot phase of the PREDIMED study.
**c)** A leaflet with written information about the main food components and cooking habits of the MeDiet, together with recommendations on the desired frequency of intake of specific foods, is given to the each participant. Participants assigned to the MeDiet group receive an additional leaflet on health benefits, use, and conservation of olive oil, while those in the MeDiet+Nuts are given a leaflet with similar information regarding nuts, with emphasis on the three nut types used in the trial (walnuts, hazelnuts, and almonds).
**d)** The participant is scheduled for a group session in the next 1-2 weeks. The visit ends with an agreement to participate in the group session.

D. 6.2.2. Group sessions. The PREDIMED dietitian runs the sessions. No more than 20 participants may attend. Separated sessions are organized for each intervention group (MeDiet or MeDiet+Nuts). The group session includes:

**a)** An introductory talk by the dietitian to remind the 14-item score.

**b)** Clarification of possible doubts about face-to-face counseling or written material.

**c)** The following written material is given to each participant and discussed with them:

-Description of 4-5 food items typical of the MeDiet and adapted to the season of the year.

-A quantitative 1-week buying list of food items, according to the season of the year.

-A weekly plan of meals (with detailed menus) adapted to the buying list.

-The cooking recipes for cuisine practices according to the suggested menus.

**d)** The needed amount of either olive oil (15 liters = 1 liter/week for 15 weeks) or sachets of walnuts, hazelnuts, and almonds (1,350 g walnuts = 15 g/d; 675 g hazelnuts = 7.5 g/d, and 675 g almonds = 7.5 g/d, allotment for 90 days) are provided to participants, together with instructions about their use and conservation. To improve compliance, a 500-g pack of walnuts is given to each family unit.

**e)** The dietitian facilitates the exchange of messages and ideas included in section D. 6.2.1 b) among the members of the group.

**f)** The contact ends with an agreement to participate in the next visit (in the next 3 months).

In the MeDiet+Nuts group we offer participants three kinds of nuts: walnuts, hazelnuts, and almonds, instead of providing only one type of nut, because we have received funding from the nut industry to provide the three of them. As stronger evidence may support that ALA-rich walnuts can offer special advantages in cardiovascular prevention, we are supplying a higher amount of walnuts.

D. 6.2.3. Follow-up visits and reiteration of individual and group sessions

The individual and group visits are repeated every 3 months with the same contents (D.6.2.1 and D.6.2.2). Each visit includes three steps: assessment, intervention, and future directions.

## D. 7 Description of intervention diets

Our main focus is in changing the **dietary pattern** instead of focusing in changes in macronutrients. Total fat intake for the 2 intervention groups is *ad libitum* (a high fat intake is allowed, as long as most fat is derived from fatty fish and vegetable sources, particularly olive oil or nuts). There is also no specific energy restriction. The two intervention diets will strongly differ from the diet recommended to the Control group in relevant micronutrients, mainly ALA present in walnuts and polyphenols in EVOO, together with flavonoids, other polyphenols, and sterols present in nuts and EVOO.

The common general guidelines to follow the MeDiet that PREDIMED dietitians provide to participants in the two intervention groups include the following positive recommendations: a) abundant use of olive oil for cooking and dressing dishes; b) consumption of >=2 daily servings of vegetables (at least one of them as fresh vegetables in a salad), without counting garnishing c) >=2-3 daily serving of fresh fruits (including natural juices); d) >=3 weekly servings of legumes; e) >=3 weekly servings of fish or seafood (at least one serving of fatty fish); f) >=3 weekly servings of nuts or seeds; g) select white meats (poultry without skin or rabbit) instead of red meats or processed meats

(burgers, sausages); h) cook regularly (at least twice a week) with tomato, garlic and onion adding or not other aromatic herbs, and dress vegetables, pasta, rice and other dishes with tomato, garlic and onion adding or not other aromatic herbs. This sauce is made by slowly simmering the minced ingredients with abundant olive oil. Negative recommendations are also given to eliminate or drastically limit the consumption of the following foods: cream, butter, margarine, cold meat, paté, duck, carbonated and/ or sugared beverages, pastries, industrial bakery products (such as cakes, donuts or cookies), industrial desserts (puddings, custard), French fries or potato chips, and out-of-home pre cooked cakes and sweets.

The dietitians insist that 2 main meals per day should be eaten (seated at a table, lasting more than 20 minutes); for usual drinkers, the main source of alcohol should be wine (maximum 300 ml, 1-3 glasses of wine per day). If wine intake is usual, a recommendation to drink a glass of wine per day (bigger for men, 150 cc, than for women, 100 cc) during meals is given. *Ad libitum* consumption is allowed for the following food items: nuts (raw and unsalted), eggs, fish (recommended for daily intake), seafood, low fat cheese and whole-grain cereals. Limited consumption (=<1 serving per week) is advised for cured ham, red meat (after removing all the visible fat), chocolate (only black chocolate, with more than 50% cocoa), cured or fatty types of cheese.

D. 8.1 Menu development

Most of the studies that have examined the Mediterranean diet have been conducted under relatively controlled conditions, with most foods and dishes given to a reduced sample of participants by the research team. The PREDIMED trial represents a further step to obtain more relevant information for public health use, because the nutritional intervention is undertaken in free-living persons, who receive information, motivation, support and empowerment to modify their food habits in a real-life context, i.e. they continue to buy their foods and cook their meals. Such an intervention provides a realistic scenario that may be easily applied to public health policies. However, since palatability of meals is extremely important to ensure compliance, menus and recipes with these characteristics for the two intervention diets have been developed. Menus are designed to meet the nutrient targets. They are provided to the participants and they may learn to prepare the menus using the recipes and the information given by the dietitian.

D. 8.2. Food supply and distribution

A 15-liter supply of EVOO rich in polyphenols (®Hojiblanca, Spain) is provided every 3 months to each participant in the MeDiet group. Similarly, every 3 months a supply of 1,350-g walnuts (®California Walnut Commission, Sacramento, Cal), 675-g almonds (®Borges SA, Reus, Spain), and 675 h hazelnuts (®La Morella Nuts, Reus, Spain) is provided to each participant assigned to the MeDiet+Nuts group. Depending on personal preference and convenience, the participants collect the free foods at the PCC at the time of the 3-monthly group session or have the supplies shipped to their homes. The three nut industry companies are committed to supply for free the nuts used in the study until its termination. The Hojiblanca Company has agreed to supply the olive oil for free during the year 2004, and our agreement with the company has to be renewed every year. We also have approached the International Olive Oil Council as a second option for additional

supply of olive oil. None of the investigators has any conflict of interest with these food companies, and there is a complete freedom to publish the findings of the study.

D. 8.3. Adherence promotion

Efforts to promote adherence began at the earliest stages of the study. During screening and orientation, participants are repeatedly provided with information about key features of the study and with the concept of MeDiets. At the first screening visit, the attitude towards dietary change is assessed in the eligibility questionnaire. Individuals must be willing to change their diets, otherwise they are excluded. The dietitian-led motivational and education intervention includes both individual and group sessions every 3 months, totaling more than 32 intervention visits during the trial. Additional written material is provided. Furthermore, the free distribution and supply of key food items ensures a high adherence to the intended diets during the trial. Acceptance of the intervention protocol is increased because no specific caloric restriction is imposed and participants are allowed an *ad libitum* fat intake, if it comes from olive oil, nuts, other plant-sources or fatty fish. However, after randomization, every effort is made to promote adherence. In many instances, these efforts are tailored to the specific needs of the participant (e.g. food items delivered to home or work).

D. 8.4. Compliance assessment

The yearly administered FFQ will provide information about compliance and attainment by participants of the nutrients targets. Although the FFQ that we are using has been previously validated in Spain (**Martin-Moreno, 1993**), we will conduct a sub-study during 2004-05 to validate and calibrate it again. In this substudy we will include 150 additional participants, not participating in the major trial, but recruited in the same way and with a similar age and gender distribution, who will complete 2 FFQ, one at baseline and another at the end of 1 year, together with four 3-day food records separated by 3 months. This process will ensure a better quality in the measurement of actual diets and will allow corrections for measurement errors in the FFQ.

At any rate, the information extracted from the FFQ will only provide a subjective assessment of compliance. To obtain also an objective evaluation, we will measure biological markers of compliance in a random subset of participants from the three arms of trial. In a random sample of 10% of participants, a blood sample and urine aliquots will be used to blindly ascertain the following markers of compliance: a) plasma fatty acid composition (specially oleic and ALA, which are reliable indicators of MUFA and walnut consumption, respectively); b) urinary tyrosol and hydroxytyrosol (EVOO); c) urinary resveratrol and ethanol (wine and other alcoholic beverages). To relate these measurements to the time of intake, participants are asked the time spent since they last consumed the specific foods when blood and urine samples are taken.

## D. 8 Measurements

Table 1 displays major measurements and data collection activities by visit.

**Table 1. Overview of measurement scheduled in the PREDIMED trial.**

| | BASELINE | YEAR-1 | YEAR-2 | YEAR-3 | YEAR-4 |
|---|---|---|---|---|---|

| | | | | | |
|---|---|---|---|---|---|
| **1. ELIGIBILITY QUESTIONNAIRE** | **X** | | | | |
| **2. GENERAL QUESTIONNAIRE*** | **X** | | | | |
| **3. FOOD FREQ. QUESTIONNAIRE** | **X** | **X** | **X** | **X** | **X** |
| **4. PHYS. ACTIV. QUESTIONNAIRE** | **X** | **X** | **X** | **X** | **X** |
| **5. 14-ITEMS SCORE OF MEDIET** | **X** | **X** | **X** | **X** | **X** |
| **6. FOLLOW-UP QUESTIONNAIRE*** | | **X** | **X** | **X** | **X** |
| **7. EKG** | **X** | **X** | **X** | **X** | **X** |
| **8. BLOOD SAMPLE** | **X** | **X** | **X** | **X** | **X** |
| **9. URINE SAMPLE** | **X** | **X** | **X** | **X** | **X** |
| **10. TOE NAIL SAMPLE** | **X** | | | | |

* Includes measurements of weight, height, waist circumference, BP, and ankle-brachial blood pressure index.

D. 8. 1 Questionnaires

Five questionnaires (#1 to 5 in table 1) have already been described in this proposal. The PREDIMED dietitians are responsible for the accurate filling of the questionnaires.

The follow-up questionnaire (#6 in table 1) collects information about the following issues:

-Socio-demographic variables (changes since baseline): 7 items.
-Changes in smoking habits: 3 items.
-New medical diagnoses of diabetes, hyperlipidemia or hypertension: 3 items.
-New medical diagnoses of cardiovascular events: 10 items.
-Inquiries about non-cardiovascular complications of diabetes: 3 items.
-Other medical conditions: 3 items.
-Current use of medication (including doses): 20 items.
-Time since the last intake of EVOO.
-Time since the last intake of wine or other alcoholic beverages.

D. 8. 2 Blood pressure (BP) and anthropometric measurements

BP and body weight and height are measured by PREDIMED nurses, who are trained and certified for these measurements. We use numerous quality control procedures to promote accurate measurements. We are tracking the performance of nurses in collecting this information (e.g. checking for digit preference and excessive variation on replication measurements). We are training, certifying and recertifying nurses every 6 months. For BP measurement, participants rest quietly for five minutes in the seated position. A validated semi-automatic sphygmomanometer (Omron HEM-705CP) is used for the PREDIMED trial. An appropriate sized cuff is applied after measurement of arm circumference. A pulse obliteration pressure is obtained. At each visit, 3 measurements will be obtained, separated by 2 minutes. The average of second and third measurement is written in the data collection form. If both measurements differ more than 5 mmHg, the whole procedure is repeated and additional BP readings are averaged. Weight is measured using a calibrated balance beam scale with the subject barefoot and wearing light clothes. Height is measured by the nurse using a wall-mounted calibrated stadiometer. Waist circumference is measured using an anthropometric measuring tape, at a horizontal plane midway between the lowest rib and the iliac crest.

D. 8. 3 Electrocardiograms (EKG)

At each yearly visit, the nurse obtains from the clinical record the last available EKG, and collects 2 copies. If no EKG has been taken during the last year, she communicates with the PCC physician to perform a new EKG and 2 copies are included in the PREDIMED file of the participant.

D. 8. 4 Extraction, processing and storage of biological samples

The PREDIMED nurses are directly responsible for collection, processing and storage of biological specimens. All PREDIMED nurses are experienced and registered nurses trained and certified to perform the specimen collection protocol. Training, before starting the trial, consisted of approximately 4 hours of theoretical information and 4 hours of practical instruction. Blood samples are collected at baseline and in yearly follow-up visits according to the protocol depicted in table 2.

**Table 2. Blood samples taken each year.**

| | Number of tubes | Volume (ml) | |
|---|---|---|---|
| **Glass tube K3E EDTA** | **4** | **4.5** | **EDTA Plasma + buffy coat** |
| **Plastic tube K3E EDTA (cold)** | **1** | **3** | **Cold EDTA plasma (pre-refrigerated tube)** |
| **Glass tube 9NC Citrate** | **1** | **4.5** | **Citrate Plasma + buffy coat** |
| **Gel-Glass tube SST** | **2** | **4** | **Serum (one of them light-protected for Hcy)** |
| **Glass tube K3E EDTA*** | **1** | **5** | |
| **TOTAL** | **9** | **38.5** | |

*Only a random sample (10%) is analyzed for biomarkers of compliance: the remaining is stored for future analyses.

The plastic tube K3E EDTA (cold) and 1 gel-glass tube SST (for homocysteine) will be refrigerated in ice prior to blood collection; after blood is collected, the tubes will be kept cold in the ice container. Serum, citrate plasma and EDTA plasma samples will be distributed in aliquots of 650 microliters and stored at -80℃ for later analyses in the central laboratory. Biochemical measurements will be performed blindly and in the same batch for consecutive samples of each participant. Every FC has acquired a refrigerator with enough capacity to store these specimens. A urine sample will be taken yearly from each participant, and 16 aliquots (500 microliters) will be stored at -80ºC. Depending on the available funding for the PREDIMED trial, some of the blood samples could be omitted on years 1-4. All biological samples will be processed at each FC not later than 2 hours after collection. During transport from the PCC to the FC laboratory, they will be stored in a portable cooler (-4ºC). A clip of each toenail of each participant is stored at room temperature.

In addition, a complete blood count and routine biochemical measurements will be performed yearly in the PCC (fasting blood glucose, uric acid, ALT, AST, gamma-glutamyl transpeptidase, alkaline phosphatases, bilirubin, creatinine, BUN; total, HDL-, and LDL-cholesterol; triglycerides, total protein, and albumin) together with a routine urine exam including the albumin/creatinine ratio in a recent sample. Serum biomarkers of inflammatory status (VCAM-1, ICAM-1 and IL-6) and oxidative stress (MDA) will be determined in a subsample of participants. In addition, in a random sample of participants,

genomic DNA has been isolated from leucocytes and some polymorphism in candidate genes that may modulate the cardiovascular response to diet (hepatic lipase, lipoproteinlipase, PPARG, APOE, paraoxonase, MTHFR, and others) have been analyzed by PCR and further allelic discrimination. Some members of our team have played a relevant role in identifying gene-diet interactions which can be of interest in our project (**Ordovas, 2001; Corella, 2002**).

D. 8.5 Outcome ascertainment

Outcomes will be determined by review by the **Clinical End Point Committee**. This panel will be blinded to the intervention group. The primary outcome is a composite of major cardiovascular events that includes cardiovascular death, definite non-fatal myocardial infarction (MI), coronary revascularization procedures, and ischemic stroke. Cardiovascular deaths will be ascertained by the Clinical Event Subcommittee from clinical registers on the basis of the clinical record and a death certificate listing an International Classification of Diseases code corresponding to any cardiovascular death (CHD or stroke). A definite non-fatal MI refers to a report of a clinical MI by a clinical center that meets the criteria for MI described in the Manual of Operations. Myocardial infarction is defined by the presence of symptoms suggestive of ischemia or infarction, with either electrocardiographic evidence (new Q waves in two or more leads) or cardiac-marker evidence of infarction, according to the standard American College of Cardiology definition **(Cannon, 2001).**

-Revascularization procedures include coronary by-pass, angioplasty, and thrombolytic procedures.

-Stroke diagnosis is based on rapid onset of a neurological deficit lasting more than 24 hours, supported by imaging studies (CT or MR scans).

Secondary analyses will be done for death by any cause, heart failure with pulmonary edema, new-onset diabetes mellitus, dementia, and cancer incidence other than non-melanoma skin cancer. The Clinical End Point Committee will follow standard procedures for attribution of these outcomes, as specified in the Manual of Operations.

**D. 9 Statistical Analysis**

There will be two primary analyses. The first will compare the incidence of major cardiovascular events in the MeDiet+EVOO group with the control diet group. The second analysis will compare the same outcome between the MeDiet+Nuts group and the control diet group. We do not expect to have enough statistical power for the comparison of one intervention (MeDiet+EVOO) with the other (MeDiet+nuts). Therefore, the aforementioned two analyses will be the primary measures of efficacy. The implications of multiple comparison issues are minimal with this planned analysis. P-values will be always two-sided. Primary analyses will be based in the intention-to-treat principle. If the results are as expected (see estimates for RR in C.3.4), a useful potential application for public health will be obtained because several alternatives can be offered to the population. For subjects who do not follow a MeDiet, this pattern can be recommended. For subjects who already follow a MeDiet, adding nuts can be recommended. For those who do not feel able to follow a MeDiet, they can be recommended to supplement their diet with nuts. Secondary analyses will be done for death by any cause, heart failure with pulmonary edema, new-onset diabetes mellitus, new-onset dementia, and cancer incidence other than non-

melanoma skin cancer. We will analyze also changes in blood lipids, BP, markers of inflammation and other intermediate markers of cardiovascular risk in a subsample of participants, selecting a random sample of at least 300 participants from each group. These comparisons will allow us to better understand the mechanisms by which the experimental diet acts on cardiovascular risk. Depending on the available funding at the closure of the trial we will expand the number of determinations and the number of subjects. Analysis of variance with Dunnet post-hoc contrasts versus the control group will be the primary comparison in these analyses. In addition, gene-diet interaction in determining intermediate and final phenotypes will be examined in a sub-sample of participants.

The Data and Knowledge Management Center (DKMC) will be responsible for preparing reports to monitor the progress of the study for the Steering Committee and the Data and Safety Monitoring Board (DSMB). The main variable for analysis will be time from randomization to event. For a given outcome, the time of the event will be defined as the number of days from randomization to the first post-randomization diagnosis, as determined by the adjudicator. For silent MI, the date of the follow-up EKG will be applied. Participants without a diagnosis will be censored at the time of last follow-up contact. The generally accepted method for analyzing this type of data is the logrank statistic. It has the advantage of requiring no assumptions other than the random assignment of the intervention. This analysis will be the primary measure of the success or failure of the trial. However, if baseline imbalances between groups are observed for any of the main CHD risk factors, a Cox proportional hazard analysis will be conducted, stratified by FC, age, gender and major risk factors. Primary outcome comparisons will be estimated as hazard ratios. Adjustment for other relevant variables (body mass index, physical activity, education level, marital status) will be also applied.

To assess the progress of the daily operation of the study, the DKMC will prepare routine reports for the Steering Committee. These reports will focus on the general status of a) participant recruitment; b) participant adherence; c) quality control; d) clinical outcome data at each center. No endpoint will be included in the Steering Committee Reports.

The DSMB reports will be prepared for each DSMB review and will be tailored to meet the needs of the Committee, including: a) general progress of study and recruitment (person-years of follow-up in comparison to targets stated in advance); b) endpoints (group comparisons with respect to both the primary outcome, total mortality and the other secondary outcomes); c) adherence (biochemical markers and FFQ data of compliance and comparison with targets for nutrients and groups of foods); d) data quality (digit preference, variability, outliers). In these sections, data will be provided for the study as a whole and, where appropriate, separately for each FC. Reports will be mailed to members of the DSMB two weeks prior to the meeting or to the date of review. Steps will be taken to insure security and confidentiality, including distribution by certified mail and enactment of a return policy of all reports. Comparison of groups with respect to major outcomes will be updated two days before the meeting so that the DSMB will have the most up-to-date data possible.

Data will be analyzed at the statistical unit of the DKMC with the support of the statistical units of IMIM (Barcelona) and University of Navarra (Pamplona), which will reassure the quality of the results by following well-established analytical quality assessment procedures. Subgroups specified prospectively for analysis of intervention effects are: a) gender; b) age; c) diabetes status at baseline; d) plasma LDL and HDL; e)

hypertension; f) smoking; g) pre-trial level of adherence to MeDiets; h) center (FC). The data from the PREDIMED trial will be analyzed at 5 intervals (4 interim analyses and the final analysis). After interim analyses the study will be terminated or extended if warranted. There are 3 potential reasons for early ending of the trial: a) efficacy of MeDiet may be demonstrated; b) harmful effects of the intervention may be discovered; c) there may be no hope for a reasonable evaluation of the proposed hypothesis (i.e., if a small intervention differential exists, power may be seriously compromised and early termination of the trial may be considered). In the case of a greater than expected observed benefit, early termination should be considered only if the intervention effect is great, and a conservative rule to stop the trial has been adopted. We have selected the O'Brien-Fleming boundaries (**O'Brien, 1979**), using asymmetric lower and upper boundaries (see below). The 4 interim analyses to ascertain the continuation of the PREDIMED trial will be conducted after 1, 2, 3 and 3.5 years of median follow-up. The 2-sided p-values for stopping the trial at each interim analysis ($1^{st}$ to $4^{th}$) are respectively $5*10^{-6}$, 0.001, 0.009 and 0.02 for benefit and $9*10^{-5}$, 0.005, 0.02 and 0.05 for adverse effects (each p value corresponding to the a normal distribution value $z_i=(P*(5/i))^{0.5}$, where i is the $i^{th}$ comparison and P is taken from **O'Brien, 1979** for alpha=0.05 and alpha=0.10). The actual recommendation regarding stopping or continuing the trial will be made by the DSMB. The result of the statistical test is important but it will be one of many considerations in making these decisions.

**D.10 Sample size**

A sample size of 9,000 participants will provide sufficient statistical power to investigate the effect of the MeDiet+EVOO and MeDiet+Nuts on cardiovascular events. The sample size estimates were computed by comparing two binomial proportions representing the event rates in one of the treatment groups and in the control group, using the following equation:

$$N = 2\ (z_{(1\text{-beta})}+z_{(1\text{-alpha/2})})^2[P_1\ (1-P_1)+ P_2\ (1-P_2)]/(\ P_1 - P_2)^2.$$

where $P_1$ and $P_2$ are the proportion of participants that have events after 4 years in the control and intervention groups, respectively. We assumed a 80% power (1-beta=0.8) and (1-alpha/2=0.975) for a 2-sided p-value of 0.05. To estimate the projected cumulative incidence in the control group, we used the Framingham tables that predict 10-year absolute risks for CHD in the next ten years. For a 69-year man and a 69-year woman with 3 risk factors (e.g. smoker, stage I hypertensive, total cholesterol=260 mg/dl) the 10-year risks are 21% and 15%, respectively: Averaging and adapting these figures to a 4-year follow-up, a 7% average CHD absolute risk can be assumed. The higher sensitivity in the definition of myocardial infarction (i.e., including troponin) may compensate for the lower risk that is expected in participants in a trial. Ischemic stroke is also included in the composite primary outcome, this adds a further 5% absolute risk, predicting a 12% overall event rate after 4 years in the control group. The minimum decrease in number of events in the intervention group that is desired to be detected is 20%. Then $p_1$ = 0.12 and $p_2$= (0.12)(0.8) = 0.096. The total required number of participants with these assumptions would be 2,625 in each group. We are including 3,000 subjects in each group to allow for 10% losses to follow-up (Table 3).

**Table 3. Sample size considerations.**

| **Expected scenario at the end of trial** |
|---|

| Group | n recruited | (90% follow-up) n (followed-up) | Expected Events | Minimum detectable RR (expected 95% CI) | Statistical Power |
|---|---|---|---|---|---|
| MeDiet | 3,000 | 2,700 | 302 | 0.80 (0.69-0.92) | 86.7% |
| MeDiet+Nuts | 3,000 | 2,700 | 302 | 0.80 (0.69-0.92) | 86.7% |
| Control group | 3,000 | 2,700 | 378 | 1 (ref.) | |

**Power calculations: sensitivity analysis** **(AR=absolute risk in control group)**

| | 95% follow-up | | | 90% follow-up | | | 85% follow-up | | |
|---|---|---|---|---|---|---|---|---|---|
| AR= | (0.14) | (0.12) | (0.1) | (0.14) | (0.12) | (0.1) | (0.14) | (0.12) | (0.1) |
| **RR= 0.75** | 99% | 98% | 95% | 99% | 97% | 94% | 98% | 97% | 93% |
| **RR= 0.80** | 93% | 88% | 81% | 92% | 87% | 79% | 90% | 85% | 77% |

**RR= relative risk. CI= confidence interval. AR=absolute risk (control group).**

In the sensitivity analysis for power calculations (Table 3) we present several assumptions regarding follow-up rate (95%, 90% and 85%), absolute risk after 4-year follow-up in the control group (0.14, 0.12 and 0.1) and relative risks (0.75, 0.80). For relative risks around 0.8 we will have enough power in the different potential scenarios. We believe that our assumptions are realistic, because previous trials and observational studies of MeDiets have found a stronger reduction in risk than what we are assuming here. The only arguable assumption might be an artificially high absolute risk for a Mediterranean population. However, the incidence of stroke in the Spanish population of these ages is substantial, and almost approximates the incidence of CHD. Unfortunately, there are no population registries to document the incidence of non-fatal cardiovascular disease, but according to the last available mortality data from Spain, CHD caused 38,688 deaths and stroke caused 36,420 deaths in year 2000 (http://cne.isciii.es). Therefore including stroke in the composite outcome of cardiovascular disease may almost double the expected number of events, estimated with the Framingham table of CHD risk. Furthermore, the use of survival analysis methods (log-rank test) will improve the statistical efficiency of the analyses and will substantially increase the power.

## D. 11 Organizational structure

The project will be supervised by the DSMB. The Project Leader, Dr. Ramon Estruch (Hospital Clinico, Barcelona), is assisted by the Steering Committee to take the strategic decisions along the project life. The coordination, research and internal supervision activities of the project will be performed by the following three categories of units: coordination units, research and operative units, and subcommittees. Table 4 shows data of each unit. In summary, the research team includes 11 Field Centers (FC), two Data Management and Statistical Units (DMSU); a **Data and Knowledge Management Center** (DKMC); and 5 specialized laboratory units (SLU). The **Steering Committee,** chaired by Dr. Estruch, includes the coordinators of each of the 11 FC, the investigator responsible of the each SLU and the Data Manager. There are also the following organizational structures: a **Diet Subcommittee**, co-chaired by Dr. Martinez-Gonzalez (Navarra) and Dr. Ros (Barcelona), a **Measurement Subcommittee**, chaired by Dr. Covas IMIM-Barcelona), a **Recruitment Subcommittee** (chaired by Dr. Salas), a **Clinical End Point Committee**, chaired by Dr. Aros (Cardiologist, Txagorritxu Hospital, Vitoria) and will include a clinician from each FC. The **Data and Safety Monitoring Board** (DSMB) has been established and it includes the following external advisors: Dr. F. Xavier Pi-Sunyer, Columbia

University College of Physicians and Surgeons, New York; Dr. Frank B. Hu, Harvard School of Public Health; Dr. Joan Sabaté, Loma Linda University, CA; Dr. Carlos A. González, Principal Investigator of the EPIC cohort in Barcelona, Spain. They will be convened to review the implementation of the protocol and to monitor trial progress on an annual basis. In addition, as stated, a mailed report will be sent them periodically with the pertinent analyses to ascertain the continuation of the PREDIMED trial. Most of the Committees will convene by conference calls and at in-person meetings (twice per year during years 1-4, once during year 5). The frequency of contacts will vary during the trial.

**Table 4. Description of Project Organic Units.**

| Categ. | Unit name | Responsibilities |
|---|---|---|
| Coordina-tion | Administration | General management, accounting, task schedule management. |
| | Technological Support | Technical and technological assistance in computing services, instrument installation and maintenance. |
| | Data and Knowledge Mana-gement Center (DKMC) | Statistical analysis, data management, report elaboration, database management, software support tools development, security of information and computer systems. |
| | Medical Support | Medical assistance in clinical tasks |
| Research and Operative | Barcelona University (Hospital Clinico) | **FIELD CENTER** (**FC**)-1: Patient recruitment, dietary behavioral intervention<br>**Specialized Laboratory Unit** (**SLU**)-1 |
| | Barcelona, IMIM, Primary Health Care | **FC-2**: Patient recruitment, dietary behavioral intervention<br>**Data Management and Statistical Unit** (**DMSU**)-1, **SLU-2** |
| | Navarra University | **FC-3** Patient recruitment, dietary behavioral intervention, **DMSU-2,** FFQ processing |
| | Valencia University | **FC-4**: Patient recruitment, dietary behavioral intervention, genetic analysis, **SLU-3** |
| | Malaga University | **FC-5**: Patient recruitment, dietary behavioral intervention, laboratory work |
| | Seville University | **FC-6**: Patient recruitment, dietary behavioral intervention |
| | Seville Primary Health Care | **FC-7**: Patient recruitment, dietary behavioral intervention |
| | Seville-CSIC* | **SLU-4** |
| | Mallorca, I. Balears University | **FC-8**:Patient recruitment, dietary behavioral intervention |
| | Tarragona, Rovira Virgili Univ. | **FC-9**: Patient recruitment, dietary behavioral intervention, laboratory work |
| | Vitoria, Txagorritxu Hospital | **FC-10**: Patient recruitment, dietary behavioral intervention |
| | Madrid, Hospital Carlos III | **FC-11**: Patient recruitment, dietary behavioral intervention |
| | Zaragoza University | **SLU-5** |

*CSIC: Superior Council for Scientific Research. IMIM: Municipal Institute for Medical Research.

## D. 12 Data management

The DKMC will be co-directed by the Data Manager, Dr. Covas, and Dr. Martinez-Gonzalez. With the support of the DMSUs (IMIM and Navarra University), they will develop and maintain the data base; and will provide statistical and trial monitoring support throughout the field work. Data from screening, intervention and follow-up visits will be entered on specific forms at the FC, and sent monthly to the Data Manager in the DKMC, who will send reports of missing or inappropriate entries, for clarification and resolution, to the FC coordinators every month. The Data Manager will also provide monthly reports to Dr. Estruch on the quality and completeness of the data, organized by type of visit (screening visit 1, 1-year follow-up visit, etc.) and by specific data form. At the end of each yearly visit in each FC, the Data Manager will verify the completeness of data for each individual.

A web based system of data access has been created (www.predimed.uji.es/drupal) where all the forms and datasets and published papers can be downloaded by authorized investigators. For privacy and security, an ID and password are required to access the data and the forms. This web-based system can be also used to send data to the Data Manager. Quality control reports will be generated for key aspects of the trial, e.g., digit preference and variability. This web-based system can be also used to send data to the

Data Manager. Quality control reports will be generated for key aspects of the trial, e.g., digit preference and variability. To reduce data entry expenses and speed processing, the questionnaires and data forms can be optically scanned. The data forms will be entered in duplicate and missing data checks will be performed. After data entry, cross-form edit checks will also be performed. Data inconsistencies will be checked. Audits will be rerun periodically to detect unresolved problems. Standardized edit reports that summarize problems in the database provide an additional method of assuring data quality. To minimize the potential for error, we have developed a detailed Manual of Operations. In addition we will conduct annual training meetings for staff. The DKMC will monitor the performance of each FC and will recommend new or corrective procedures in case deficiencies are noted. Until the end of the trial, all FC will be masked to trial outcome data, with the exception of the statisticians, the Data Manager and the external DSMB. Due to the nature of the intervention, however, dietitians and nurses at each FC need to be unmasked to diet assignment. The Clinical Event Subcommittee will be blinded to participant allocation.

**D. 13. Design considerations**

In our deliberations, we considered alternative designs. One set of considerations related to the type of study (behavioral intervention versus feeding study). Controlled feeding studies are the best procedure to assess the biological effect of nutrients on intermediary markers of cardiovascular risk. However, the existence of multiple pathways for the effects of dietary exposures on CHD, some of them newly identified and still some of them that will be very likely identified in the next years may render intermediate surrogate markers of CHD risk as misleading. These considerations strengthen the need to study clinical outcomes. If clinical outcomes are to be observed, a controlled feeding design is completely unfeasible. Second, from a public health perspective, a behavioral intervention coupled with an easy (free) access to the supposedly healthy food represents a more realistic test of the effectiveness to be attained with public policies and health promotion activities in nutrition and public health. The purported benefits of diets rich in ALA led us to adopt the three group design, because we would assess the effect of two interventions, one with MeDiet and another with an ALA-rich MeDiet (walnuts are rich in ALA), both compared with a control group. We acknowledge this design has problems of feasibility. These problems would substantially increase should we adopt a factorial (2x2) design. Therefore we opted for a simpler, three-group randomization.

**D.14 Limitations of the study**

The control group is not receiving a special behavioral intervention and controls continued in fact following a diet rich in fat and not very different in its macronutrient composition from the diet of the intervention groups (although a sizeable amount of MUFA was derived from meat instead of olive oil or nuts). Therefore, the macronutrient composition of the compared diets did not provide a strong contrast. We acknowledge this limitation because controls belong to a Mediterranean culture and their food habits are not very far from the MeDiet. But we think that it would be unethical to strongly advice controls to get apart from this supposedly healthy diet. Instead, we are requesting additional funding to increase differences by using a comprehensive and expanded intervention in both experimental groups to attain further changes in their diet, thus maximizing the contrast.

The joint effect of minor changes in classical risk factors with dramatic changes in markers of inflammation can be associated with a substantial reduction in risk.

The main differential point in our design is that we are evaluating two MeDiets which are higher in fat than all diets assessed by previous trials, but in both of them the sources of fat are mainly MUFA. In addition, the effect of diets rich in polyphenols and phytosterols is also assessed. The rigorous methodology of the PREDIMED trial should convincingly answer what is certainly one of the major sources of controversy and unresolved issues regarding diet and cardiovascular risk, namely, the impact of replacing saturated fat with MUFA or with carbohydrate.

## References


Albert CM, et al (2002). Arch Intern Med 162:1382-7.
Anderson KJ, et al (2001). J Nutr 131:2837-42.
Ascherio A, et al. BMJ 313:84-90.
Baylin A, et al. (2003). Circulation 107:1586-91.
Bazzano LA, et al (2001). Arch Intern Med 161:2573-8.
Becker, 2002. Eur J Clin Invest 32:1-8.
Bray GA, Popkin BM (1998). Am J Clin Nutr 68:1157–73.
Canga N, et al (2000). Diabetes Care 23:1455-60
Cannon CP, et al (2001). J Am Coll Cardiol 38:2114-30.
Carluccio MA, et al (1999). Arterioscler Thromb Vasc Biol 19::220-8.
Carluccio MA, et al (2003). Arterioscler Thromb Vasc Biol 23:622-9.
Connor WE, Connor SL (1997). N Engl J Med 337:562-3.
Corella D, et al (2002). J Lipid Res 43:416-27.
Costacou T, et al (2003). Eur J Clin Nutr 57:1378-85.
De la Puerta, et al (1999). Biochem Pharmacol 57:445–9.
De la Puerta, et al (2001). Life Sci 69;1213–22.
De Lorgeril M, et al (1999). Circulation 99:779-85.
Desideri , et al (2002). Clin Chim Acta 320:5-9.
Djousse L, et al (2003). Am J Clin Nutr 77:819-25.
Ellsworth JL, et al (2001). Nutr Metab Cardiovasc Dis 11:372-7.
Elosua R, et al (2000). Med Sci Sports Exerc 32:1431-7.
Elosua R, et al (1994). Am J Epidemiol 139:1197-209.
Feldman EB (2002). J Nutr 132:1062S–1101S.
Ferrara LA, et al (2000). Arch Intern Med 160:837-42.
Ferro-Luzzi A, et al (2002). Eur J Clin Nutr 56:796-809.
Fraser GE, et al (1992). Arch Intern Med 152:1416-24.
Fuentes F, et al (2001). Ann Intern Med 134:1115-9.
Garcia-Lorda P, et al (2003). Eur J Clin Nutr 57:S8-11.
Guallar E, et al (1999). Arterioscler Thromb Vasc Biol 19:1111-8.
Haim , et al (2002). J Am Coll Cardiol 39:1133-8.
Hu FB, et al (1998). BMJ 317:1341-5.
Hu FB, et al (1999). Am J Clin Nutr 69:890-7.
Hu FB (2003). N Engl J Med 348:2595-6.
Hwang et al (1997). Circulation 96:4219-25.
Jacobs DR Jr, Steffen LM (2003). Am J Clin Nutr 78(Suppl):508-13S.
Jacques PF, Tucker KL (2001). Am J Clin Nutr 73:1-2.
Jequier E, Bray GA (2002). Am J Med 113(9B):41S–46S.
Joint WHO/FAO Expert Consultation on Diet, Nutrition and the Prevention of Chronic Diseases (2002: Geneva, Switzerland). WHO technical report series; 916. Geneva: WHO, 2003.
Kouris-Blazos A, et al (1999). Br J Nutr 82:57-61.
Kris-Etherton PM, et al (2001a). Circulation 103:1823-5.
Kris-Etherton PM, et al. (2001b). Nutr Rev 59:103-11.
Lasheras C, et al (2000). Am J Clin Nutr 71:987-92.
Lercker G, Rodriguez MT (2000). J Chromatogr A 881:105-29.
Lopez-Azpiazu I, et al (2000). Public Health 114;183-9.
Luc, et al (2003). Atherosclerosis 170:169-76.Malik, et al (2001). Lancet 358:971-6.
Manna C, et al (2002). J Agric Food Chem 50:6521-6.
Martinez-Gonzalez MA, Estruch R (2004a). Eur J Cancer Prev (in press).
Martin-Moreno JM, et al (1993). Int J Epidemiol 22: 512-9.
Marrugat J, et al. Eur J Nutr (in press).
Mata P, et al (1997). Arterioscler Thromb Vasc Biol 1::2088-95.
Mataix J, et al (1998). Free Radic Biol Med 24: 511-21.
McManus K, et al (2001). Int J Obes 25:1503-11.
Miles, et al (2001). Clin Sci (Lond) 100:91-100.
Mossavar-Rahmani Y, et al. J Am Diet Assoc (in press).
Nigg CR, et al (1999). Gerontologist 39:473-82.
O'Brien PC, Fleming RT (1979). Biometrics 35:549-56.
Oomen CM, et al (2001). Am J Clin Nutr 74:457-63.
Ordovas JM, et al (2002). Circulation 106:2315-21.
Panagiotakos DB, et al (2002). Prev Med 35:548-56.
Patterson RE, et al (2003). J Am Diet Assoc 103:454-60.
Perez-Jimenez F, et al (2002). Atherosclerosis 163:385-98.
Pradhan, et al (2002). JAMA 288:980-7.
Puerta-Vazquez R, et al. Metabolism (in press).
Ramirez-Tortosa, et al (1999). J Nutr 129:2177-83.
Ridker, et al (2000a). N Engl J Med 342:836-43.
Ridker et al (2000b). Circulation 101:1767–72.
Ros E (2003). Am J Clin Nutr 78(suppl):617S-25S.
Sabaté J (2003). Am J Clin Nutr 78(suppl):647S-50S.
Sanchez-Villegas A, et al (2003). Eur J Clin Nutr 57:285-92.
Singh RB, et al (2002). Lancet 360:1455-61.
Tanne, et al (2002). Stroke 33:2182-6.
Trichopoulou A, et al (1995). BMJ 311:1457-60.
Trichopoulou A, et al (2003). N Engl J Med 348:2599-608.
Tunstall-Pedoe H, et al (1999). Lancet 353:1547-57.
USDA (2000). Nutrition and Your Health: Dietary Guidelines for Americans. Washington: US Department of Agriculture. http://www.health.gov/ dietaryguidelines/ dga2000/ document/ contents.htm.
Visioli F, Galli C (1998). Nutr Rev 56:142-7.
Visioli F, et al (2000). Circulation 102:2169-71.
Visioli F, Galli C (2002). Crit Rev Food Sci Nutr 42:209-21.
Willett WC, et al (1995). Am J Clin Nutr 61(Suppl):1402-6S.
Willett WC (2000). Proc Soc Exp Biol Med 225:187-90.
World Health Organization (1993). Health for All: statistical database. Geneva: WHO, Regional Office for Europe.
World Health Organization (2003a). Neglected Global Epidemics: three growing threats. In: WHO: The world health report 2003 - shaping the future. Geneve: WHO.
World Health Organization (2003b). Joint WHO/FAO Expert Consultation on Diet, Nutrition and the Prevention of Chronic Diseases. Geneva. WHO technical report series; 916.
Yaqoob, et al (1998). Am J Clin Nutr 67:129-35.

Mangiapane EH, et al (1999). Br J Nutr 82:401-9.
Martinez-Gonzalez MA, et al (2002). Eur J Nutr 41:153-60.

Appendix. **Quantitative Score of Compliance with the Mediterranean Diet**

| | **Foods and frequency of consumption** | **Criteria fo** |
|---|---|---|
| 1 | Do you use olive oil as main culinary fat? | Yes |
| 2 | How much olive oil do you consume in a given day (including oil used for frying, salads, out of house meals, etc.)? | 4 or more |
| 3 | How many vegetable servings do you consume per day? *(1 serving = 200g - consider side dishes as 1/2 serving)* | 2 or more *or as sala* |
| 4 | How many fruit units (including natural fruit juices) do you consume per day? | 3 or more |
| 5 | How many servings of red meat, hamburger, or meat products (ham, sausage, etc.) do you consume per day? *(1 serving = 100-150 g)* | Less than |
| 6 | How many servings of butter, margarine, or cream do you consume per day? *(1 serving = 12 g)* | Less than |
| 7 | How many sweet/carbonated beverages do you drink per day? | Less than |
| 8 | How much wine do you drink per week? | 7 or more |
| 9 | How many servings of legumes do you consume per week? *(1 serving = 150 g)* | 3 or more |
| 10 | How many servings of fish or shellfish do you consume per week? (1 serving: 100-150 g fish, or 4-5 units or 200 g shellfish) | 3 or more |
| 11 | How many times per week do you consume commercial sweets or pastries (not homemade), such as cakes, cookies, biscuits, or custard? | Less than |
| 12 | How many servings of nuts (including peanuts) do you consume per week? *(1 serving = 30 g)* | 3 or more |
| 13 | Do you preferentially consume chicken, turkey o rabbit meat instead of veal, pork, hamburger or sausage? | Yes |
| 14 | How many times per week do you consume vegetables, pasta, rice, or other dishes seasoned with *sofrito* (sauce made with tomato and onion, leek, or garlic, simmered with olive oil)? | 2 or more |

* 0 points if these criteria are not met.

# PREDIMED STUDY

# MEDITERRANEAN DIET IN THE PRIMARY PREVENTION OF CARDIOVASCULAR DISEASE

**Amendments to the Research Protocol**

## Amendments to the Research Protocol

After reviewing the results of the pilot study based on the analysis of the short-term effects of 2 Mediterranean diets versus those of a low-fat control diet on intermediate markers of cardiovascular risk (Ann Intern Med 2006;145:1-11), the Steering Committee decided to perform the following modifications:

1. Oct 2005-One of the initial recruiting centers (Hospital Carlos III, Madrid) abandoned the study after completion of the pilot study. The PI of this center claimed insurmountable logistic difficulties with recruitment to justify his decision.

2. Jan-2006- Another recruiting center (Sevilla University) also abandoned the study in early 2006 after the completion of the pilot study. The PI of this center also claimed insurmountable logistic difficulties with recruitment to justify his decision.

3. Jul 2006- To compensate for the loss of recruiting power, we included two new recruiting centers, thus the number of centers was kept at 11.

4. Oct-2006. Include dietary intervention in the low-fat (control) group.

   The PREDIMED group sessions were organized separately for each of the 3 intervention groups. Participants were provided with written material (see: http://www.predimed.org and http://www.predimed.es) including descriptions of seasonal foods, shopping lists, weekly meal plans and cooking recipes. The control group received the same type and intensity of dietary intervention than the two Mediterranean diet groups, although the recommendations for total fat intake were opposite those given to participants in the two Mediterranean diet groups. Advice on vegetables, meats and processed meats, high-fat dairy products, and sweets concurred with the Mediterranean diet, but use of olive oil for cooking and dressing and consumption of nuts and fatty fish were discouraged. A 9-item quantitative score of compliance with the low-fat control diet was constructed (**Table 1**) as an instrument for dietitians to assess and modify the participant's dietary pattern but, unlike the 14-item Mediterranean diet score, it was not an intervention outcome. The last assessment of the 9-item score helped dietitians to give personalized advice in order to upgrade it in a similar way than the 14-item Mediterranean diet score was instrumental to enhance the Mediterranean diet in the corresponding intervention groups. Similarly, accomplishments in the previous months were used as support to provide further empowerment and self-reward.

5. March 2007-Inclusion Criteria

In the initial protocol we considered as one risk factor criterion for inclusion a plasma HDL-cholesterol concentration ≤40 mg/dl without gender specification. Based on newly published definition of gender-specific HDL-cholesterol values for cardiovascular risk assessment, we changed this criterion to HDL-cholesterol ≤40 mg/dl in men and ≤50 mg/dl in women.

6. May 2007- Validation of the dietary assessment tools (questionnaires).

   The 137-item Food Frequency Questionnaire (FFQ) used in the Predimed trial included a previously validated FFQ (Martin-Moreno, et al. Int J Epidemiol 1993; 22: 512-9.) plus some additional items which were more specific for our trial. During the course of the study we had the opportunity to repeat the validation study of this FFQ. The validation field work was done during 2005-2007 and the results were eventually published (Fernández-Ballart JD. et al. Br J Nutr. 2010 Jun;103(12):1808-16 and de la Fuente-Arrillaga C, et al. Public Health Nutr. 2010 Sep;13(9):1364-72).
   In addition, we have also published the validation study for the brief 14-item questionnaire of adherence to the Mediterranean Diet used in the trial (Schröder H, et al. J Nutr. 2011 Jun;141(6):1140-5).

7. Apr-2008: Change of Sample Size

   The sample size was recalculated after the DSMB meeting in April 2008 on the basis of the observed rates of cardiovascular events during the first 2 years of follow-up. The ALLHAT trial included similar participants and observed an 8.9% cumulative rate for the primary outcome (fatal CHD + non-fatal myocardial infarction) after 4.9 years of follow-up (***JAMA* 2002; 288: 2998-3007)**. Adapting this figure to a 6-year follow-up and including also stroke in the end-point definition, an 11% absolute risk in the control group could be conservatively assumed in our study. We expected a 25% relative risk reduction in both MeDiet groups. Under these assumptions, the total number of participants required was 5631 (1877 per group) for beta=0.2 and 2-tailed alpha=0.05. We needed to include more than 7400 subjects to allow for both 10% losses during follow-up and a lower incidence than expected.

8. Apr-2008. Completion date of the trial

   According to the new sample size calculation, the anticipated completion date of the trial was changed to December, 2011.

# PREDIMED STUDY

# MEDITERRANEAN DIET IN THE PRIMARY PREVENTION OF CARDIOVASCULAR DISEASE

**Research Protocol**

**May 2009**

# PREDIMED STUDY. MEDITERRANEAN DIET IN THE PRIMARY PREVENTION OF CARDIOVASCULAR DISEASE

## Research Protocol

### A. BACKGROUND. SPECIFIC AIMS

We propose to conduct a large controlled, randomized clinical trial in a high-risk population aimed to assess whether a Mediterranean diet (MeDiet) enriched with extra-virgin olive oil (EVOO) or mixed nuts prevents cardiovascular disease (cardiovascular death, non-fatal myocardial infarction, or non-fatal stroke) in comparison with a Low-fat Control diet. As secondary outcomes, we will also assess the effects of the MeDiet on all-cause mortality and the incidence of heart failure, diabetes, cancer, cognitive decline, and other neurodegenerative disorders.

Food habits are critical determinants of health at both individual and population level (**Hu, 2002**). The MeDiet has been widely considered as a model of healthy eating. Findings from two large European cohort studies, the follow-up of the Greek EPIC cohort (**Trichopoulou, 2003**) and the HALE study in the elderly (**Knoops, 2004**), as well as from two large US cohorts, the NIH-AARP Diet and Health study (**Mitrou, 2007**) and the Nurses' Health study (**Fung, 2009**) suggest that a high degree of adherence to the MeDiet is associated with a reduction in both total mortality and coronary heart disease (CHD) mortality. In a randomized trial with a modified MeDiet (enriched with alpha-linolenic acid but not with little olive oil) versus a control diet, the Lyon Diet Heart Study (**De Lorgeril, 1999**), concluded that the MeDiet was associated with a remarkable reduction in CHD event rates and cardiovascular mortality. However, no randomized controlled trial has ever been conducted to assess whether a MeDiet is superior to the usually recommended low-fat diet in the primary prevention of CHD.

The potential cardiovascular preventive effect of MeDiets in the face of the increasing global burden of CHD (**Reddy, 2004; WHO, 2003a**) makes the answer to this question a public health priority. Other reasons to perform a large study on the cardioprotective effects of the MeDiet include: a) the long tradition of following MeDiets without any harm in Southern Europe, where life expectancy is quite high (**Willett, 1995**), b) the low incidence of CHD in these countries (**Tunstall-Pedoe, 1999),**, in spite of having similar or even higher levels of classical risk factors compared to the US population (**Gabriel, 2008**); c) the diversity of mechanistic and epidemiological observations of beneficial effects on cardiovascular health of the consumption of distinctive components of the MeDiet, such as monounsaturated fatty acids (MUFA) from olive oil (**Pérez-Jiménez, 2005**) or nuts (**Kris-Etherton, 2001)**; and d) the higher palatability, acceptance and compliance of MeDiets in comparison with low-fat diets (**McManus, 2001**).

In the era of evidence-based medicine, sound nutritional recommendations for the general public should be based on the results of large clinical trials with "hard" end-points, but this level of evidence is not yet available for the Med-Diet or its main components (i.e., olive oil). In October 2003 we started the recruitment of participants for such a primary prevention trial, the **PREDIMED (PREvención con DIeta MEDiterránea) Study**. This parallel group, multi-center, randomized study is funded in part by a grant from the official biomedical research agency of the Spanish government, the Instituto de Salud Carlos III (ISCIII), as a trial aimed at assessing the effects on the risk of major cardiovascular events

of two intensive behavioral counseling and nutrition education interventions supplemented with extra-virgin olive oil (EVOO) or nuts in comparison with a group encouraged to follow a low-fat diet

**A.1 Primary Aims**

- To assess the effects of the MeDiet on a composite endpoint of cardiovascular death, myocardial infarction, and stroke in comparison with a Low-fat, Control diet.
- To assess separately the effects of a MeDiet supplemented with EVOO and a MeDiet supplemented with mixed nuts, both in comparison with a control low-fat diet on a composite on a composite endpoint of cardiovascular death, myocardial infarction, and stroke.

**A.2 Other Aims**

We will also ascertain changes in secondary outcomes: death of any cause and incidence of heart failure, diabetes mellitus, dementia, and cancer; and intermediate outcomes, such as blood pressure (BP), fasting blood glucose, lipid profile, markers of inflammation, and other intermediate markers of cardiovascular risk to better understand how dietary changes are able to modify the risk of clinical events.

**B. BACKGROUND AND SIGNIFICANCE**

Cardiovascular disease is the main cause of death worldwide at the turn of the XXI century. Western countries, including the US, currently continue to exhibit unacceptably high absolute rates of cardiovascular morbidity and mortality. Furthermore, these diseases constitute emerging and neglected epidemics in developing countries (**Reddy, 2004; WHO, 2003a**, **WHO, 2003b**). Surprisingly, a low incidence of CHD is found in some developed countries such as France, Spain, Greece, Italy, and Portugal, leading to a higher life expectancy as compared with Northern European countries or the US (**WHO, 1993; Tunstall-Pedoe, 1999**). The Mediterranean food pattern has been the factor most frequently invoked to explain this health advantage.

**B.1 The Mediterranean diet (MeDiet)**

The MeDiet is identified as the traditional dietary pattern found in olive-growing areas of Crete, Greece, and Southern Italy in the late 1950s and early 1960s. Its major characteristics are: a) a high consumption of grains, legumes, nuts, vegetables, and fruits; b) a relatively high-fat consumption (up to 40% of total energy intake), mostly from MUFA, which accounts for 20 percent or more of the total energy intake; c) olive oil for culinary use and dressing of vegetables as the principal source of fat; d) moderate to high fish consumption; e) poultry and dairy products (usually as yogurt or cheese) consumed in moderate to small amounts; f) low consumption of red meats, processed meats, and meat products; g) moderate alcohol intake, usually in the form of red wine consumed with meals (**Trichopoulou, 1995; Martinez-Gonzalez, 2004a**).

The background of a long and ancient tradition with no evidence of harm makes the MeDiet a very promising tool for public health. Wide sectors of the scientific community and consumers believe in the cardioprotective role of the MeDiet. This hypothesis fits well into the current paradigm of studying dietary patterns instead of isolated foods or nutrients in nutritional epidemiology (**Hu, 2002**). The rationale is that foods and nutrients may have synergistic or antagonistic effects when they are consumed in combination. Further, overall

patterns better represent dietary practices found in free-living populations, therefore providing useful epidemiological information (**Jacques, 2001; Jacobs, 2003**). Dietary patterns also have a higher potential for acceptability, palatability and future compliance when they are recommended in behavior counseling. In spite of its relatively high fat content, the theoretical advantages of the MeDiet pattern are multiple. To increase vegetable consumption, the fact that fat-free or low-fat dressings are less acceptable than the use of olive oil or other full-fat salad dressings. The sautéing or stir-frying of vegetables with variable amounts of olive oil instead of using low fat spreads or steaming increases taste and results in long-term maintenance of a vegetable-rich diet. These preparation and cooking techniques are typical of Mediterranean countries, where cooking of vegetables in olive oil to enhance flavor is customary. Hence, in health promotion and nutritional education, a better compliance with MeDiet could be expected. In fact, a trial of weight loss with hypocaloric diets (**McManus, 2001**) reported a better adherence to a MeDiet than to a low-fat diet. Participants viewed this diet as more tasty than low-fat regimens, and this led to an increased long-term compliance. Nevertheless, an undesirable departure from the traditional MeDiet has occurred in Southern European countries, especially among younger people (**Sanchez-Villegas, 2003; Costacou, 2003**), with increasing consumption of red meats, processed meats, and sugar-containing foods and drinks. In addition, the traditional consumption of EVOO is being increasingly replaced by refined vegetable oils of inferior quality.

## B.2. Available evidence on the MeDiet and cardiovascular prevention

A MeDiet was inversely associated with mortality from all-causes in several small observational cohort studies of elderly people (**Trichopoulou, 1995**; **Kouris-Blazos, 1999**; **Lasheras, 2000**). Findings from the Greek EPIC cohort, with more than 22,000 participants, suggested that a higher adherence to the MeDiet is associated with a reduction in total mortality and, more specifically, in coronary mortality (**Trichopoulou, 2003**). Similar findings have been reported from the follow-up of the HALE study in healthy persons aged 70 to 90 years (**Knoops, 2004**), the NIH-AARP Diet and Health study (**Mitrou, 2007**) and the Nurse's Health study (**Fung, 2009**). Two case-control studies also found an inverse association between adherence to the MeDiet and incidence of non-fatal coronary events (**Martinez-Gonzalez, 2002**; **Panagiotakos, 2002**). A secondary prevention trial found a remarkable reduction in reinfarction or death when coronary patients were assigned to an experimental MeDiet (**De Lorgeril, 1999**) and with dairies replaced by a special margarine rich in alpha-linolenic acid (ALA). The American Heart Association has given attention to MeDiets as potentially useful for the prevention of CHD, but it also warns that more studies are needed before the population can be advised to follow a MeDiet. These studies should disentangle whether the diet itself plays a major role in protection from CHD, together with other factors (such as more physical activity or stronger social support systems) characteristic of Mediterranean countries (**Kris-Etherton, 2001a**). A recent meta-analysis underlined the benefits of adherence to a MedDiet on overall and cardiovascular mortality (**Sofi, 2008**).

There are also many epidemiological evidences suggesting that consumption of either olive oil or nuts, both integral components of the MeDiet, provides cardiovascular protection. The frequent intake of both foods showed inverse associations with CHD incidence in observational studies and improved several cardiovascular risk markers in

small, short-term clinical studies (**Fuentes, 2001**), and in a recent 2-year trial in 180 patients with the metabolic syndrome (**Esposito, 2004**). Again, no randomized clinical trials have been conducted to assess the effects of olive oil or nuts on cardiovascular prevention.

## B.3 Limitations of published studies on the MeDiet and cardiovascular prevention

The only published trial using a so-called "MeDiet", the Lyon Diet Heart Study (**De Lorgeril, 1999**), included patients who had already experienced a clinical event, i.e. it was a secondary prevention trial, and its results may not be extrapolated to primary prevention. Besides, the results of this trial, showing a 50-70% reduction in reinfarction and CHD mortality in the group assigned the MeDiet rich in alpha-linolenic acid, seem to be too good to be true, and major aspects of the design and methods have been criticized. However, no special consideration was given to olive oil, which is the major source of dietary fat in Mediterranean countries (**Martinez-Gonzalez, 2004b**). On the other hand, no clear protective effect of olive oil was observed in the Greek EPIC cohort (**Trichopoulou, 2003**), thus raising the issue of whether the large amount of fat provided by olive oil is in fact affording protection against CHD (**Hu, 2003**). In the Lyon Diet Heart Study, dietary assessments at baseline and at the end of the study were reported for only a subset of participants (30% of the control group and 50% of the experimental group). In addition, no biochemical markers of adherence were obtained. Thus, the diet followed by most participants completing the trial is not known (**Kris-Etherton, 2001a**; **Robertson, 2001**). A third major issue was that only 13% of energy intake was provided by MUFA in the group assigned to the so-called "MeDiet" in the Lyon trial. This value for MUFA intake is far below the ≥20% content of the traditional MeDiet (**Perez-Jimenez, 2002**). Additional concerns about the Lyon Diet Heart study are related to the low number of observed endpoints (44/14, in the control and intervention group respectively), the improbably large reduction in relative risk (RR) (in spite of the lack of changes in most classical risk factors), and the fact that the trial was stopped early (after 27 months of follow-up). No study has assessed a true MeDiet rich in olive oil for cardiovascular endpoints. The PREDIMED study is the first large trial to randomize high-risk patients to a traditional MeDiet and a control, low-fat diet for primary cardiovascular prevention, thus overcoming previous limitations and aiming to provide the best quality of evidence

## B.4. Olive oil and cardiovascular prevention

A characteristic feature of the typical MeDiet is the elevated consumption of olive oil as the main source of added fat. The presumed antiatherogenic properties of olive oil have been mainly attributed to its high oleic acid content. However, in recent years converging evidence indicates that polyphenols,present in EVOO, but not in common refined olive oils (ROO), may contribute to the benefits of its consumption (**Pérez-Jiménez, 2005**). The concentration of phytochemicals in oils is influenced by the oil extraction procedures. EVOO is obtained from the first pressing of the ripe fruit and has a high content in antioxidants (tocopherols, polyphenols, flavonoids) and phytosterols. Lower quality ROO lose antioxidant capacity because polyphenols are lost in the refining process, although fatty acid composition is similar to that of EVOO (**Ramirez-Tortosa, 1999**; **Lercker, 2000**). EVOO phenolics (mainly, hydroxityrosol and tyrosol) have shown strong antioxidant and antiinflammatory activity *in vitro* (**De la Puerta, 1999**, **2001**;

**Manna**, **2002**; **Visioli**, **2002**). In crossover studies that compared EVOO with ROO at similar doses to those of the usual MeDiet, EVOO increased total plasma antioxidant capacity (**Visioli, 2004**) and LDL resistance to oxidation (**Marrugat**, **2004**), In a large crossover randomized study in Europe, in vivo markers of lipid and LDL oxidation were decreased in a dose dependent manner with the phenolic content of the olive oil (**Covas, 2006**).

There are, however, discrepancies regarding the potential of EVOO to influence LDL oxidizability (**Vissers, 2004**). EVOO has also been shown to afford better protection than dietary alpha-tocopherol against lipid peroxidation (**Mataix, 1998**) and to have antiinflammatory properties (**Puerta-Vazquez, 2004**). Consequently, ROOs are thought to provide less cardiovascular benefit than EVOO, and it is important to distinguish between each type of oil when analyzing the effects of olive oil on cardiovascular risk. In a small feeding trial in hypercholesterolemic men, a MeDiet with EVOO improved endothelial function compared to a low-fat diet (**Fuentes, 2001**). In another study in patients with hypertension, EVOO significantly reduced the need for antihypertensive medication (**Ferrara, 2000)**.

The isocaloric replacement of saturated fat with MUFA from olive oil causes a reduction of total and LDL cholesterol comparable to that attained by low fat diets, but HDL cholesterol is maintained at higher levels, thus obtaining a net advantage on the lipid profile (**Perez-Jimenez, 2002; Willett, 2000**). In addition, LDL particles enriched with oleic acid are more resistant to oxidation. High olive oil consumption, the hallmark of the MeDiet (**Hu, 2003**), is partially responsible for these effects. Additional mechanisms have been suggested for the beneficial effect of the MUFA-rich MeDiet (**Perez-Jimenez, 2002)**. Among them (see below), it has been reported that incorporation of oleic acid into cultured endothelial cells decreases the expression of endothelial leukocyte adhesion molecules with reductions in vascular cell adhesion molecule-1 (VCAM-1) and inhibition of nuclear factor-kappa B activation (**Carluccio, 1999**). Postprandial factor VII activation is attenuated by a MUFA-rich diet. Olive oil is also associated with a reduced DNA synthesis in human coronary smooth muscle cells (**Mata, 1997**). In view of the healthy properties of olive oil, the US Food and Drug Administration recently approved a health claim for it to be labeled as a putative cardioprotective food. However, no clinical studies with cardiovascular outcomes have used olive oil.

**B.5. Nuts and cardiovascular prevention**

There are consistent epidemiological evidences to support a cardioprotective effect of nut consumption. In a large Californian cohort, the Adventist Health Study, the frequency of nut intake was inversely associated with the cardiovascular risk (**Fraser, 1992**). More recently, the results of three other large cohorts, the Iowa Women's Health Study, the Nurses' Health Study, and the Physician's Health Study, have confirmed that frequent nut consumption is associated with a lower CHD risk (**Ellsworth, 2001**; **Hu, 1998**; **Albert, 2002**). However, according to the results of the Iowa cohort the relationship between nut consumption and CHD risk did not reach statistical significance. Also, the Physician's Health Study cohort only found protection for sudden cardiac death, but not for non-sudden coronary death or nonfatal myocardial infarction associated with high nuts consumption.

Several small randomized trials (<50 subjects) have shown consistent decreases in total cholesterol and LDL-cholesterol with diets enriched with a variety of nuts, although most studies have investigated almonds or walnuts (**Kris-Etherton, 2001b**). The

hypocholesterolemic effect is achieved with intakes amounting to two or three servings per day. Effects on HDL-cholesterol have been inconsistent. When evaluated, the ratios of total cholesterol to HDL cholesterol decreased (**Feldman EB, 2002**). A walnut-rich diet has been reported to improve endothelium-dependent vasodilatation and to reduce levels of VCAM-1 (**Ros, 2004**). These mechanistic results suggest that frequent nut consumption may decrease the risk of CHD.

Walnuts, almonds, hazelnuts, and other nuts, like pine nuts, are common staples in the traditional MeDiet, since they are locally produced throughout the Mediterranean basin. Nuts are very high in fat (48-63 g/100 g of edible portion). Most nuts are rich in MUFA (mostly, oleic acid), whereas walnuts are high in PUFAs (linoleic and alpha-linolenic acids). The dietary fiber content in nuts is high, ranging from 5 to 9% by weight. Nuts are good sources of arginine, potassium, vitamin E, and other bioactive compounds. Thus, the unique composition of nuts may help explain the beneficial effects observed in prospective cohorts and in short-term feeding trials. No clinical studies with cardiovascular outcomes have used nuts. The PREDIMED study will be the first large trial to randomize high-risk patients to receive nuts for cardiovascular prevention.

**B. 6. High-fat diets based on MUFA in diabetics, overweight subjects and high risk individuals**

Traditionally, nutrition advice in diabetics, obese subjects, and those with cardiovascular risk factors emphasized avoiding animal fat and, preferably, all kinds of dietary fat, and replacing them with carbohydrate (CHO). The rationale was that fats provided an excess of energy, thus, they are thought to promote obesity. However, scientific evidence has been accumulated in the last two decades about the beneficial role of diets with a relatively high MUFA content on cardiovascular risk factors, obesity, and diabetes. These beneficial MUFA are provided by the MeDiet and, specifically, by olive oil (**Ros, 2003**; **Bondia-Pons**, **2007**) and most nuts (**Ros, 2003; Sabaté, 2003; Garcia-Lorda, 2003)**. In fact, the frequent intake of simple CHO in many otherwise low-fat foods is associated with weight gain. However, when nutrition advice is given to people with obesity or diabetes, reluctance still exists to recommend high-fat, high-MUFA diets as an alternative to the traditional (and less palatable) low-fat diets. By the design of the trial, a sizeable number of the PREDIMED participants are either overweight/obese or type 2 diabetics. It is thus important to recognize that there is no evidence that a higher percentage of fat in the diet in the form of MUFA results in increased body weight. The lack of a fattening effect of such MUFA-rich diets has been shown in the context of controlled diets (**Ros, 2003**), weight-reduction programs (**McManus, 2001; Shai, 2008**), and *ad libitum* diets (**Sabaté, 2003**). Furthermore, the results of our pilot study (see below) are also sobering with respect to the lack of weight gain in the intervention groups.

**B. 7. Significance of primary prevention randomized trials**

The fact that conclusions based on the results of observational studies have been subsequently refuted by evidence from clinical trials (ie, the presumptive cardioprotective effects of estrogen replacement therapy or vitamin E supplementation) has raised major concerns. This reinforces the necessity of obtaining first level evidence before considering any global public health strategy to promote the MeDiet as a model of healthy eating. This level of evidence in primary prevention is only obtained by conducting large-scale

randomized trials with cardiovascular events as the principal outcome. Dietary guidelines can be safely issued when consistency is found between observational and experimental studies.

Because of the limitations in feasibility for randomized trials to assess the long-term effects of dietary interventions on clinical outcomes, the scientific community has relied on the combination of findings of epidemiological investigations and short-term studies with intermediate endpoints. However, a secondary prevention trial using food patterns as main interventions has been conducted with more than 600 participants (**De Lorgeril, 1999**). We believe that a primary prevention trial is also feasible because analysis of the first 1700 participants in the PREDIMED study after follow-up for 1 year showed that the behavioral intervention plus delivery of specific foods modified the participants' dietary habits and cardiovascular risk profile in the expected direction (**Zazpe, 2008**). The PREDIMED intervention focuses on diet, not on other lifestyle components; hence the results should answer the question on whether the MeDiet is indeed cardioprotective.

## C. PRELIMINARY STUDIES

### C. 1. Preliminary trials of olive oil and nuts

Among other relevant published trials, we highlight two recent studies from members of our research team.

#### C. 1.1. Phenolic content in dietary EVOO decreases in vivo LDL oxidation

A randomized, double-blind, crossover feeding trial using three similar olive oils but with increasing phenolic content (from 2.7 mg/kg to 366 mg/kg) was conducted in 200 European healthy volunteers. Olive oils were administered over three periods of 3 weeks preceded by two-week washout periods. All olive oils increased HDL cholesterol and the ratio between reduced and oxidized glutathione and decreased triglycerides and DNA oxidation. Olive oils with medium and high phenolic content decreased in vivo lipid and LDL oxidation. The increase in HDL cholesterol and the decrease in the in vivo lipid oxidative damage were observed in a dose-dependent manner with the phenolic content of the olive oil administered (**Covas**,**2006; Machowetz**, **2007**).

#### C. 1.2 A walnut diet improves endothelial function in hypercholesterolemic subjects

In a crossover design, 21 hypercholesterolemic men and women were randomized to a cholesterol-lowering MeDiet and a diet of similar energy and fat content in which walnuts replaced about 32% of the energy from MUFA (**Ros, 2004**). Participants followed each diet for 4 weeks. The walnut diet improved endothelium-dependent vasodilatation and reduced levels of VCAM-1 ($P<0.05$ for both). The walnut diet significantly reduced total and LDL cholesterol ($P<0.05$ for both). In a complementary study, walnuts added to a test meal rich in saturated fatty acids reduced postprandial endothelial dysfunction in both healthy and hypercholesterolemic volunteers (**Cortés**, **2006**). A cardioprotective effect of nut intake beyond cholesterol lowering was shown by the results of both studies, suggesting that a walnut-enriched MeDiet may provide even further benefits for cardiovascular prevention.

### C. 2 Preliminary case-control study of MeDiet and the risk of myocardial infarction

The protection against CHD afforded by the MeDiet was assessed in a case-control study of myocardial infarction cases and healthy subjects (**Martinez-Gonzalez, 2002)**. Six

food items were considered protective: 1) olive oil, 2) fiber, 3) fruits, 4) vegetables, 5) fish and 6) alcohol. A score of 1 to 5 corresponding to his/her quintile of intake of each of these items was assigned to each participant. The quintile score was opposite for two other elements assumed to be harmful: 7) meat/meat products and 8) some carbohydrate-rich items with high glycemic load (white bread and other items). The eight quintile values were summed for each participant to build a MeDiet score. The higher the MeDiet score, the lower was the odds ratio (OR) of myocardial infarction. A significant linear trend was apparent after adjustment for the main cardiovascular risk factors. For each additional point in the score (observed range: 9–38) the OR (95% confidence interval, CI) was 0.92 (0.86–0.98). Our data supported the hypothesis that increasing compliance with a MeDiet can be an effective approach for reducing the risk of CHD.

**C. 3. Pilot study of the PREDIMED trial**

Our pilot study included the first 772 participants of the PREDIMED trial (339 men and 433 women, mean age 67 ± 6 years) completing intervention for 3 months. They were asymptomatic patients, half of them with diabetes and the remaining with at least three risk factors. We implemented the first steps of an intensive behavioral counseling and nutrition education intervention in two groups to recommend participants to follow a MeDiet. In the control group we gave a simple advice on the prudent, low-fat diet. In the first intervention group (MeDiet+EVOO), participants received a free supply of EVOO (1 liter/week). In the second intervention group (MeDiet+Nuts), participants received instead a free supply of 30 g/d of nuts (15 g walnuts, 7.5 g hazelnuts and 7.5 g almonds). Three months after randomization, we assessed changes in diet, BP, fasting blood glucose, insulin sensitivity (in nondiabetic participants), lipid profiles, and circulating inflammatory molecules. The changes of foods and nutrients in the 3 intervention groups were in the expected direction. Compared with the low-fat diet, the 2 MeDiets produced beneficial changes in most markers, while participants' weight remained stable in spite of increased fat intake in the two MeDiet groups (**Estruch, 2006**). In a substudy of 372 participants in the 3 groups, oxidative stress markers (circulating oxidized LDL) were reduced at 3 months in the two MeDiet groups compared with the Low-fat, Control group (**Fitó, 2007**). We expect these beneficial effects to be enhanced as the intervention progresses. The lack of weight changes with the two high-fat foods should allay fears that the promotion of the high-fat MeDiet may lead to increased adiposity.

In the pilot study (**Estruch, 2006**), there was a 76.5% increment in alpha-linolenic acid intake in the MeDiet+Nuts group, especially important, given the abundant epidemiological evidences on the cardiovascular benefits of intake of this n-3 PUFA (**Dolecek, 1991; Ascherio, 1996; Guallar, 1999; Hu, 1999**; **Baylin, 2003**; **Djoussé, 2003**; **Brouwer, 2004**), although not all studies are concordant (**Oomen, 2001**). The success of the secondary prevention trial with an experimental MeDiet (**De Lorgeril 1999**) was also attributed to a high intake of alpha-linolenic acid. Our approach compares favorably with this trial because a) we are using a typical Mediterranean food (nuts) to increase the intake of alpha-linolenic acid; b) our trial has a much larger sample size; and c) we are conducting a primary prevention trial.

Statistically significant reductions with respect to control were observed for soluble intercellular adhesion molecule-1 (ICAM-1), VCAM-1,.and interleukin-6 (**Estruch, 2006)**. A strong biological plausibility exists to support a causal association between these molecules and the risk of atherosclerosis, and a substantial body of published

epidemiologic studies is consistent in reporting that levels of ICAM-1 are significant predictors of the risk of future cardiovascular events (**Hwang, 1997; Ridker, 2000a; Malik, 2001; Tanne, 2002; Haim, 2002; Becker, 2002; Luc, 2003**) and clinical diabetes (**Meigs, 2004**). If we take into account our observed changes in ICAM-1, we can conservatively assume that changes in ICAM-1 in the MeDiet groups represent a 1 to 2-quartile changes. The corresponding published RR for CHD (**Ridker, 2000a**) are 0.77 (2:2.6) and 0.58 (1.5:2.6). Combining these effects with those attributed to the observed changes in BP and HDL cholesterol, we may expect an overall RR of 0.53 to 0.71 for the MeDiet groups. Interestingly, the level of high-sensitivity C-reactive protein (hs-PCR) only decreased in the MeDiet + EVOO group. Hs-PCR has been also reported to predict future cardiovascular events (**Ridker, 2000a; 2000b; Pradhan, 2002)** Our findings compare favorably with previous observational studies assessing differences in ICAM-1 and VCAM-1 according to n-3 fatty acid intake (7-8% reduction, **Lopez-Garcia, 2004**), and also with previous trials using either long-chain n-3 fatty acids (no effect, **Kew, 2004**), fish oils (-12% for ICAM-1 and -20% for VCAM-1, but only among older subjects, **Miles, 2001**), linseed oil (-18.7% in VCAM-1 but no change in ICAM-1, **Rallidis, 2004**), or alpha-tocopherol (-11% in ICAM-1, **Desideri, 2002**).Our results are therefore important to support the effectiveness of our dietary intervention and the rationale of the PREDIMED trial. In addition they are consistent with prior in-vitro studies suggesting that both high oleic acid intake (**Carluccio, 1999**) and EVOO polyphenols (**Carluccio, 2003**) are associated with a reduced expression of adhesion molecules. Our results are also consistent with a previous trial showing that olive oil induced a reduction in the proportion of peripheral mononuclear cells expressing ICAM-1 (**Yaqoob, 1998**) and that a nut-enriched diet favorably influenced endothelium-mediated vasodilatation and reduced VCAM-1 (**Ros, 2004**).

## D. DESIGN AND METHODS

### D.1 Summary

This clinical trial aims to assess the effects on the risk of major cardiovascular events of two intensive behavioral counseling and nutrition education interventions in comparison with a low-fat control group for a median duration of 6 years. Both intervention groups are assigned a traditional MeDiet. In one of these two groups we supplement the diet with EVOO (1 liter/week) and the other with 30 g/d of mixed nuts (15 g walnuts, 7.5 g hazelnuts, and 7.5 g almonds). The third arm of randomization is the control group, where participants do not receive education on the MeDiet, but are given advice on how to follow a low-fat diet. We are recruiting men (age: 55 to 80 years) and women (age: 60 to 80 years) with either diabetes or three or more major cardiovascular risk factors. All participants are free of cardiovascular disease at baseline. Study participants are randomized to three equally sized groups (2,350 in each). They will be followed-up for clinical outcomes during a median time longer than 5 years by the primary care physicians who recruited them for the study. The primary endpoint will be a composite outcome of cardiovascular events (cardiovascular death, myocardial infarction, and stroke).

### D.2 Design

Parallel group, multi-center, single-blind, randomized trial aimed at assessing the effects on the incidence of major cardiovascular events of intensive behavioral counseling and nutrition education interventions based on the MeDiet in comparison with a similar

intervention aimed at decreasing the total fat content of the diet. The Institutional Review Board (IRB) of the Hospital Clinic (Barcelona, Spain) accredited by the US Department of Health and Human Services (DHHS) update for Federalwide Assurance for the Protection of Human Subjects for International (Non-US) Institutions # 00000738 approved the study protocol on July, 16, 2002. This trial has been registered in the Current Controlled Trials register at London with the ISRCTN number 35739639 (http://www.controlled-trials.com/ISRCTN35739639)

Controlled feeding studies are the best procedure to assess the biological effect of nutrients on intermediate markers of cardiovascular risk. However, the existence of multiple pathways for the effects of dietary exposures on CHD, some of them newly identified and others likely to be identified in the next years, reinforces the need to study clinical outcomes. If clinical outcomes are to be observed, a controlled feeding design is completely unfeasible. From a public health perspective, a behavioral intervention coupled with an easy (free) access to representative healthy foods is a more realistic test of the effectiveness to be attained with official policies and health promotion activities in public health.

The principal aim of the study is to compare the effects of two food patterns, the MeDiet and a Low-fat Control diet, in the primary prevention of cardiovascular diseases. The rationale for using 2 MeDiet groups (one with supplemental EVOO and one with supplemental nuts) instead of one is as follows. Besides being a rich source of MUFA, the EVOO used in one arm of the study is a good source of phenolic antioxidants. One-half the dose of the nuts used in another arm of the study is made up of walnuts, thus containing sizeable amounts of PUFA, especially alpha-linolenic acid, the plant-derived omega-3 fatty acid. Thus, one MeDiet is enriched in MUFA and phenolic antioxidants and the other MeDiet is supplemented with both n-6 and n-3 PUFA. Although having the same general food pattern of the MeDiet, the two arms of the study differ in the intake of two foods (EVOO and nuts) and two nutrients (phenolics and PUFA) that are all felt to be important in cardiovascular prevention and may have differential beneficial effects. Therefore we chose a 3-group randomization design with the general aim of comparing two food patterns, the MeDiet and a low-fat diet, for cardiovascular outcomes, considering that the single foods supplemented to each MeDiet may individually contribute to the effects of the overall MeDiet food pattern. The rationale for the free provision of individual food items (olive oil or tree nuts) is that they may contribute to a higher compliance with the overall MeDiet food pattern. However, because of the high fat load of both EVOO and nuts, we felt that it was unfeasible to supplement the two foods together in a single MeDiet group of participants.

## D.3 Outcomes

- Primary outcome: composite endpoint of cardiovascular death, non-fatal myocardial infarction, and non-fatal stroke.
- Secondary outcomes: death of any cause and incidence of angina leading to a revascularization procedure, heart failure, diabetes mellitus, cancer, dementia, and other degenerative disorders.
- Other outcomes: changes in blood pressure, body weight, adiposity measures, blood sugar, lipid profile, markers of inflammation, and other intermediate markers of cardiovascular risk (see also section D.11.5)

**D.4 Sample size**

A sample size of n=6,999 with randomization to 3 equally sized groups (two intervention groups and one control group, 2,333 patients each) will provide sufficient statistical power to evaluate the effect of the Mediterranean food pattern on the primary outcome (fatal or non-fatal myocardial infarction and fatal or non-fatal stroke). The sample size estimates were computed by comparing two binomial proportions representing the event rates in one of the treatment groups and in the control group. We assumed an 80% power (1-beta=0.8) and (1-alpha/2=0.975) for a 2-sided p-value of 0.05. To estimate the projected cumulative incidence in the control group, we used the number of events observed in the first year follow-up (rate of 1.3%). Adapting this figure to a 6-year follow-up and taking into account the aging of our study population during follow-up, an 11% average absolute risk can be assumed for the control group. The minimum relative decrease in number of events in the intervention groups should be 25%. Then, $p_1$ = 0.11 and $p_2$= (0.117) (0.75) = 0.0825. With these assumptions, the total number of participants required is 1,877 per group. We plan to include 2,333 subjects in each group to allow for 10% losses during follow-up and also for a lower incidence than expected.

**D. 5 Timeline, progress and funding.**

The Spanish Ministry of Health - Instituto de Salud Carlos III (ISCIII) funded the current project for the period 2003-2005 (RTIC G03/140) and a thematic network of 16 Spanish research groups was established. In 2006 a new funding modality was established by ISCIII through the CIBER_Fisiopatología de la Obesidad y Nutrición (CiberOBN), which provided reasonable funding for one-half of the original research groups, while the other half were funded by a new research network (RTIC RD 06/0045). Other official funds from Spanish government agencies have been obtained for funding subprojects related with intermediate outcomes (lipoproteins, inflammatory markers, genomic and proteomic studies, etc.). Additional funds are obtained from other sources for the overall project, limited to small donations from Food Companies in cash and other commodities, i.e., printing of intervention material, such as recipes. Obviously, the donation by Food Companies of all the EVOO and mixed nuts needed throughout the duration of the study is a substantial contribution,

D. 5.1. Planning

The needed personnel (a minimum of a dietitian and a nurse for each of the 11 field centers - FC) were trained and certified in nutritional education and procedures for extraction and storage of blood and other biological samples, respectively, at the beginning of the study. From March to September 2003 we developed the logistics, protocols, operations' manual, and instruments, forms, and data entry/management systems. Each FC contacted approximately 20 Primary Care Centers (PCC) to recruit participants. During this same period, menus and buying lists of Mediterranean products to be provided to participants were developed.

D. 5.2. Pilot study

The recruitment for the pilot phase of the study started between October and November 2003. After 3 months, 772 participants were assessed for compliance with

dietary advice and changes in intermediate biological markers of cardiovascular risk (see section C.3).

D. 5.3. Implementation

After obtaining reassuring data confirming adequate changes in diet and risk factors during the pilot phase, recruitment went on and was finished on 31 March 2009 with a total of 7350 participants. The FC have recruited from 350 to 1000 participants, referred by approximately 20 primary care physicians for each FC.

D. 5.4. Follow-up

Participants recruited in 2003 will be followed for up to 8+ years and will have the longest follow-up. Participants entering the study during the year 2008 will be followed only for 3-4 years, thus having the shortest follow-up. Consequently, we expect a median follow-up of 6 years.

D 5.5. Analysis/closeout

Trial closeout will take place by December 31, 2011. During early 2012, the ascertainment of outcomes, laboratory measurements, data entry, and respond-to-data edits will be completed, and data will be prepared for statistical analysis. Writing of the reports for scientific publications will be done during late 2012.

**D. 6. Study population / eligibility criteria**

Trial participants consist of nearly 7,000 community-dwelling high-risk persons (≈700 in each FC), with ages 55 to 80 years for men and 60 to 80 years for women. They should be free of cardiovascular disease and meet at least one of two criteria.

D. 6.1 Inclusion criteria: either **a)** or **b)** should be met.

**a) Type 2 diabetes**. Diagnosis of diabetes is based on at least one of the following criteria: i) Current treatment with insulin or oral hypoglycemic drugs; ii) Fasting blood glucose > 126 mg/dl (fasting is defined as no caloric intake for at least 8 hours); iii) Casual blood glucose > 200 mg/dl with polyuria, polydipsia, or unexplained weight loss; iv) Blood glucose > 200 mg/dl in two measurements after an oral glucose tolerance test. **OR**

**b) Three or more** of the following **risk factors**: I) Current smoker (>1 cig/day during the last month); ii) Hypertension (systolic BP ≥ 140 mm Hg or diastolic BP ≥ 90 mm Hg or antihypertensive medication); iii) LDL-cholesterol ≥ 160 mg/dl; iv) HDL-cholesterol ≤ 40 mg/dl in men or ≤ 50 mg/dl in women, independently of lipid-lowering therapy; v) Body mass index ≥ 25 $kg/m^2$; vi) Family history of premature CHD (definite myocardial infarction or sudden death before 55 years in male $1^{st}$-degree relatives or before 65 years in female $1^{st}$-degree relatives).

D. 6.2. Exclusion criteria. Major exclusion criteria are: i) Documented history of previous cardio-vascular disease, including CHD (angina, myocardial infarction, coronary revascularization procedures or existence of abnormal Q waves in the electrocardiogram (EKG)), stroke (either ischemic or hemorrhagic, including transient ischemic attacks), and symptomatic peripheral artery disease diagnosed with vascular imaging techniques; ii) Severe medical condition that may impair the ability of the person to participate in a nutrition intervention study; iii) Any other medical condition thought to limit survival to less

than 1 year; iv) Immunodeficiency or HIV-positive status; v) Illegal drug use or chronic alcoholism or total daily alcohol intake >50 g/d; vi) Body mass index > 40 kg/m$^2$; vii) Difficulties or major inconvenience to change dietary habits; viii) Impossibility to follow a Mediterranean-type diet for religious reasons or due to the presence of disorders of chewing or swallowing; ix) A low predicted likelihood to change dietary habits according to the Prochaska and DiClemente stages of change model (**Nigg, 1999**); x) History of food allergy with hypersensitivity to any of the components of olive oil or nuts; xi) Participation in any drug trial or use of any investigational drug within the last year; xii) Institutionalized patients for chronic care, those who lack autonomy, are unable to walk, lack a stable address, or are unable to attend 3-monthly visits at their FC; xiii) Illiteracy; and xiv) Patients with an acute infection or inflammation (i.e., pneumonia) are allowed to participate in the study 3 months after resolution of their condition.

**D. 7. Recruitment**

Most FC has considerable experience and a successful track record of recruiting participants for both epidemiological studies and clinical trials. The participation of primary care physicians has ensured a high enrollment rate. Because these physicians are responsible for the usual medical care of participants and they are aware of their risk factors, no potential ethical conflict regarding confidentiality existed at the stage of identification of suitable participants for the trial. This process started by extracting their names from the records of the Primary Care Center (PCC). Most PCC (over 70 %) participating in the study have computer-based records of patients, making the selection relatively simple. The clinical records of these persons were then individually reviewed to exclude those who did not meet eligibility criteria. Candidates were contacted by telephone and invited to attend a visit in the PCC, where the purpose and characteristics of the study were explained and a signed informed consent was obtained (see below). A brief general explanation of the study, including the possibility that they might receive free allowances of olive oil or nuts for the duration of the trial, was given in this first visit. Our experience has been that >70 % of candidates approached in this way agreed to return for the screening visit.

**D. 8 Participants' visits**

Participants' eligibility for the trial was determined by review of clinical records and by two formal screening visits. In these visits, questionnaires were filled in, a clinical history was taken, anthropometric and BP measurements were made, and fasting blood for biochemical analyses was obtained. Information collected in the screening visits will also provide baseline data for subsequent analyses of the effects of the interventions on intermediary biomarkers and risk factors.

- **Pre-Screening Evaluation**: After review of clinical records, eligible candidates were contacted by telephone to know if they were both capable and willing to participate in the study. Those giving a positive response were scheduled for the first visit. Data on participation proportion (recruited:eligible ratio), collected in all field centers has been between 0.75 and 0.90.
- **Screening visit 1**: The visit, performed by the PCP, served to identify inclusion/exclusion criteria in a more comprehensive manner. This 15-30 minute visit included: a) A face-to-face administration of a 26-item questionnaire to inquire about the medical conditions and risk factors related to eligibility, including assessment of the willingness to make diet

changes (Prochaska model). b) A review of the last EKG if available in the clinical record. If no ECG had been performed within the last year, an EKG was performed during this visit. c) If the candidate met all the requirements (including EKG data), an informed consent form was given to him/her to be signed after a detailed explanation of all procedures and of the anticipated time commitment (see http://www.predimed.org). The informed consent comprised two parts, one for trial participation and biochemical analyses and another for DNA collection for genetic analyses. **d)** The following forms and questionnaires to be completed at home were provided (see http://www.predimed.org):

- A detailed written explanation of the study.
- A food frequency questionnaire (FFQ) with 137 items plus vitamin/minerals supplements (adapted from the Willett questionnaire and validated in Spain, see **Martin-Moreno, 1993**) plus specific questions for patterns of alcohol consumption.
- The Minnesota physical activity questionnaire (validated Spanish version, **Elosua, 1994 & 2000**).

**e)** The participant was instructed to collect toenail specimens and bring them in the next visit.

**f)** The next screening visit (2) was scheduled and the candidate was told to attend it after an overnight fast for blood extraction.

After the first screening visit, the participant was randomized to one of three diet groups.

- **Randomization**: The study nurse randomly assigned each participant to the corresponding intervention group following tables of random allocation according to the recruitment order. These tables were centrally elaborated by the Coordinating Unit and provided a stratified random sequence of allocation for each FC. The four strata for stratified randomization were built according to gender and age (cut-off point: 70 years). The study nurses were independent of the nurse staff of the PCC. Therefore, they were not involved in the usual clinical care of participants and their exclusive role was to collect data for the PREDIMED trial.

**- Screening visit 2**: This 1-hour visit included the following: **a)** A simplified 14-item questionnaire for assessment of adherence to the MeDiet (see http://www.predimed.org and **Martinez-Gonzalez, 2004c**). **b)** In a face-to-face interview with the candidate, the dietitian explained again in detail the purpose and development of the study. **c)** The dietitian reviewed (and completed with the candidate if needed) the FFQ and physical activity questionnaires. Alternatively, she helped the candidate who had difficulties at home to fill in the questionnaires during the visit. **d)** The nurse measured the weight, height, waist circumference, and BP. **e)** The nurse performed a venipuncture, obtained and handled blood samples, and proceeded to prepare the specified serum, plasma, and buffy-coat aliquots. **f)** A urine sample and toenail specimens were also collected by the nurse. **g)** A 47-item general questionnaire collecting information about current medication and risk factors was filled-in. Information to fill this questionnaire was also abstracted from clinical records by the research nurse.

**- Follow-up visits:** Further clinical evaluations (outside of the intervention) are limited to yearly follow-up visits**,** which include the same examinations performed at the baseline visit, with exception of the general questionnaire, which is substituted by a follow-up questionnaire, and a tolerance / adverse events questionnaire (see http://www.predimed.org ). Blood and urine samples are collected at baseline and years 1, 3, 5 and 6 (or final visit). Primary and secondary outcomes are evaluated at each follow-up visit.

**D.9. Intervention**

The PREDIMED dietitians are directly responsible for the dietary intervention. All PREDIMED dietitians are registered dietitians, trained and certified to deliver the PREDIMED intervention protocol. Before the implementation of the protocol, training consisted of approximately 24 hours of initial theoretical and practical group discussion with experts in nutrition education and discussion in between 3 to 5 conference calls to review and improve the protocol. These calls are continued bimonthly throughout the study. During the calls each dietitian discusses her practice sessions with the team, and together the group identifies problems and solutions in protocol implementation. Feedback and discussion also occur among the dietitians and the center coordinators, and between center coordinators and the Data Manager, especially after data from FFQ and objective biochemical measurements (in a random sample of 10% participants) of compliance are analyzed.

D. 9. 1. Delivery of intervention to the 3 groups

D. 9.1.1 Individual motivational interview with a PREDIMED dietitian. After screening visit 2, participants randomized to each one of the 3 treatment arms have a face-to-face interview with the dietitian. This interview includes:

**d)** The 14-item questionnaire of adherence to the MeDiet.

**e)** Personal individual recommendations for changes to be introduced in the participant's diet in order to achieve a personalized goal. Depending on treatment group, the dietitian provides a comprehensive number of reasons to either adopt a MeDiet or a low-fat diet, highlighting the advantages of the particular diet, rather than the risks of not adhering to it, and transmitting a positive message with stress on the particular benefits for diabetic patients and for those at high cardiovascular risk. For total fat intake the recommendations given to participants in the Low-fat diet group are in some way opposite those given to participants in the 2 MeDiet groups. Our previous experience with diabetic patients using this approach for a behavioral intervention to quit smoking in Primary Care has been successful (**Canga, 2000**). We also have experience in using the stages of change model for dietary change (**Lopez-Azpiazu, 2000**). The dietitian personalizes the message by adapting it to the patient's clinical condition, preferences, and beliefs. The training of the PREDIMED dietitians emphasized the holistic approach to lifestyle change in order to tailor the intervention to nutritional assessment and individual needs, and encourage adherence to the prescribed diet. A contracting procedure is used and a negotiated change in diet is the targeted goal, working with the subject to determine what he or she considers an attainable goal. The focus can be shifted from changing portion sizes to changing frequency of intake or to changes in cooking methods. Accomplishments in the previous months, even if minor, are considered as support to provide further empowerment and self-reward. The usefulness and effectiveness of this approach was shown in an even larger randomized trial in the US aimed to reduce fat intake, the Women's Health Initiative Study (**Mossavar-Rahmani, 2004; Patterson, 2003**). Importantly, caution is taken to make sure that participants with diabetes, overweight/obesity, and/or hyperlipidemia may not receive contradictory dietary advice from other health professionals external to the PREDIMED trial. Because unsaturated fats like those contained in olive oil and nuts are still perceived as fattening by some nutrition experts, it is particularly important for participants in the 2 MeDiet groups to allay the fear of an eventual weight gain that might have both the person

who is on a weight-management program and his/her nutritionist. This is done by tactful exposition of recent scientific evidences (**McManus, 2001; Ros, 2003; Sabaté, 2003; Garcia-Lorda, 2003**) and by explaining that body weight did not change after 3 months of MeDiet intervention in the pilot phase of the PREDIMED study.
**f)** Depending on group assignment, a leaflet with written information about the main food components and cooking habits of the MeDiet or the low-fat diet, together with recommendations on the desired frequency of intake of specific foods, is given. Participants assigned to the MeDiet group receive an additional leaflet on health benefits, use, and conservation of olive oil, while those in the MeDiet+Nuts are given a leaflet with similar information regarding nuts, with emphasis on the three nut types used in the trial (see: http://www.predimed.org).
**d)** The participant is scheduled for a group session in the next 1-2 weeks. The visit ends with an agreement to participate in the group session.

D. 9.2.2. Group sessions. The PREDIMED dietitian runs the sessions. No more than 20 participants may attend. Separated sessions are organized for each intervention group (MeDiet+VOO, MeDiet+Nuts, Low-fat diet). The group session includes:
**a)** An introductory talk by the dietitian to remind the 14-item MeDiet score to participants in the two MeDiet groups and a 9-item score of low-fat foods for those in the Low-fat diet group (see: http://www.predimed.org). **b)** Clarification of possible doubts about face-to-face counseling or written material.
**c)** The following written material (see: http://www.predimed.org) is given to each participant and discussed with them:

-Description of 4-5 foods typical of the MeDiet or the Low-fat diet and adapted to the season of the year.
-A quantitative 1-week shopping list of food items, according to the season of the year.
-A weekly plan of meals (with detailed menus) adapted to the buying list.
-The cooking recipes for cuisine practices according to the suggested menus.

**d)** The needed amount of either olive oil (15 liters = 1 liter/week for 15 weeks) for the MeDiet+VOO participants, sachets of walnuts, hazelnuts, and almonds (1,350 g walnuts = 15 g/d; 675 g hazelnuts = 7.5 g/d, and 675 g almonds = 7.5 g/d, allotment for 90 days) for those allocated the MeDiet+Nuts, or gifts of canned low-fat foods, books, dispensers, etc, for participants in the Low-fat diet group are provided.
**e)** The contact ends with an agreement to participate in the next visit (in the next 3 months).
In the MeDiet+Nuts group we offer participants three kinds of nuts: walnuts, almonds, and hazelnuts, instead of providing only one type of nut, because we have received funding from the nut industry to provide the three of them. As increasing evidences support that alpha-linolenic in walnuts can offer special advantages in cardiovascular prevention, we are supplying a higher amount of walnuts.

D. 9.2.3. Follow-up visits and reiteration of individual and group sessions

The individual and group visits are repeated every 3 months with the same contents, except that shopping lists and recipes vary with the season of the year. Each visit includes three steps: assessment, intervention, and future directions.

**D. 10 Description of intervention diets**

Our main focus is to change the **dietary pattern** instead of focusing on changes in single foods or macronutrients. Total fat intake for the 2 MeDiet groups is *ad libitum* (a high fat intake is allowed, as long as most fat is derived from fatty fish and vegetable sources, particularly olive oil or nuts), while it is curtailed in the Low-fat diet group. There is also no specific energy restriction. Total energy intake is adapted to participant's weight, age and requirements, and dietitians tailor advice to individual needs.

The guidelines that PREDIMED dietitians provide to participants in the MeDiet groups are based on the 14-point MeDiet score (**Table 4**), and those for participants in the Low-fat Control diet are based on the 9-point low-fat diet score (**Table 5**).

D. 10.1 Menu development

Most of the studies that have examined the MeDiet have been conducted under relatively controlled conditions, with most foods and dishes given to a reduced sample of participants by the research team. The PREDIMED trial represents a further step to obtain more relevant information for public health use, because the nutritional intervention is undertaken in free-living persons, who receive information, motivation, support and empowerment to modify their food habits in a real-life context, i.e., they continue to buy their foods and cook their meals. Such an intervention provides a realistic scenario that may be easily applied to public health policies. However, since palatability of meals is extremely important to ensure compliance, menus and recipes with these characteristics for the two intervention diets have been developed. Menus are designed to meet the nutrient targets. They are provided to the participants and they may learn to prepare the menus using the recipes and the information given by dietitians.

D. 10.2. Food supply and distribution

A 15-liter supply of EVOO rich in polyphenols (Fundación Patrimonio Comunal Olivarero, Spain) is provided every 3 months to each participant in the MeDiet group. Similarly, every 3 months a supply of 1,350 g walnuts (California Walnut Commission, Sacramento, CA), 675 g almonds (Borges SA, Reus, Spain), and 675 g hazelnuts (La Morella Nuts, Reus, Spain) is provided to each participant assigned to the MeDiet+Nuts group. Participants at each site pick up olive oil and nut allowances at the time of the 3-month group visit. Individualized methods of food delivery have been devised for occasions in which participants need to have their 3-month session rescheduled. Provisions have been made to improve participants' compliance in the 2 MeDiet groups. The olive oil allowance (1 liter/week) takes into account the needs of the whole family. Also, additional 1000 g packs of mixed nuts are provided for each family unit every 3 months. The three nut industry companies are committed to supply for free the nuts used in the study until its termination. None of the investigators has any conflict of interest with these food companies.

D. 10.3. Promotion of adherence

Efforts to promote adherence began at the earliest stages of the study. During screening and orientation, participants are repeatedly provided with information about key features of the study and with the concept of MeDiet and Low-fat diet. At the first screening visit, the attitude towards dietary change is assessed in the eligibility questionnaire. Individuals must be willing to change their dietary habits, otherwise they are excluded. The

dietitian-led motivational and education intervention includes both individual and group sessions every 3 months, totaling 32 intervention visits during the trial. Additional written material is provided. Furthermore, the free distribution and supply of key food items ensures a high adherence to the intended diets during the trial. Acceptance of the intervention protocol is increased because no specific caloric restriction is imposed and participants in the two MeDiets are allowed *ad libitum* fat intake, if it comes from olive oil, nuts, other plant-sources or fatty fish. However, after randomization, every effort is made to promote adherence. In many instances, these efforts are tailored to the specific needs of the participant (e.g. food items delivered to home or work). These procedures are very effective as evidenced by the high rates of adherence and follow-up in the pilot study.

D. 10.4. Assessment of compliance

The yearly-administered FFQ will provide information about compliance and attainment by participants of the nutrients targets. Although the FFQ that we are using has been previously validated in Spain (**Martin-Moreno, 1993**), we have performed a sub-study with 150 participants completing 2 FFQ, one at baseline and another at the end of 1 year, together with four 3-day food records separated by 3 months, to validate and calibrate it again. The validity indexes of the FFQ in relation to the food records for food groups and energy and nutrient intake had intra-class correlation coefficients between 0.40 and 0.84. Regarding food groups, between 68 and 83% of individuals were in the same or adjacent quintile using both methods. The FFQ measurements had therefore good reproducibility and a level of validity similar to those of FFQs used in other prospective studies. The validation of the FFQ ensures a better quality in the measurement of actual diets and will allow corrections for measurement errors.

At any rate, the information extracted from the FFQ will only provide a subjective assessment of compliance. To obtain also an objective evaluation, we measure biological markers of compliance in a random subset of participants from the three arms of trial. In a random sample of 10% of participants, a blood sample and urine aliquots are used to blindly ascertain the following markers of compliance: a) plasma fatty acid composition (specially oleic and alpha-linolenic acid, which are reliable indicators of MUFA and walnut consumption, respectively); b) urinary tyrosol and hydroxytyrosol (EVOO); c) urinary resveratrol and ethanol (wine and other alcoholic beverages). To relate these measurements to the time of intake, participants are asked the time spent since they last consumed the specific foods when blood and urine samples are taken.

## D. 11 Measurements

Table 1 shows major measurements and data collection activities by visit.

**Table 1. Overview of measurement scheduled in the PREDIMED trial.**

| | BASELINE | YEAR-1 | YEAR-2 | YEAR-3 | YEAR-4 | YEAR-5 | YEAR-6 |
|---|---|---|---|---|---|---|---|
| 1. ELIGIBILITY QUESTIONNAIRE | X | | | | | | |
| 2. GENERAL QUESTIONNAIRE | X | | | | | | |
| 3. FOOD FREQ. QUESTIONNAIRE | X | X | X | X | X | X | X |
| 4. PHYS. ACTIV. QUESTIONNAIRE | X | X | X | X | X | X | X |

| | | | | | | | |
|---|---|---|---|---|---|---|---|
| **5. FOLLOW-UP QUESTIONNAIRE*** | | X | X | X | X | X | X |
| **6. TOLERANCE QUESTIONNAIRE** | | X | X | X | X | X | X |
| **7. ABANDONMENT QUESTIONNAIRE**** | | X | X | X | X | X | X |
| **8. EKG** | X | X | X | X | X | X | X |
| **9. BLOOD SAMPLE** | X | X | | X | | X | X |
| **10. URINE SAMPLE** | X | X | | X | | X | X |
| **11. TOE NAIL SAMPLE** | X | | | | | | |

*Includes measurements of weight, height, waist circumference, BP, and ankle-brachial blood pressure index.
**Only if applicable.

D. 11. 1 Questionnaires

Nine questionnaires (#1 to 7 in Table 1) are shown in http://www.predimed.org. The PREDIMED dietitians are responsible for the accurate filling of the questionnaires. The follow-up questionnaire (#7 in Table 1) collects information about the following issues:

-Socio-demographic variables (changes since baseline): 7 items.
-Changes in smoking habits: 3 items.
-New medical diagnoses of diabetes, hyperlipidemia or hypertension: 3 items.
-New medical diagnoses of cardiovascular events: 10 items.
-Inquiries about non-cardiovascular complications of diabetes: 3 items.
-Other medical conditions: 3 items.
-Current use of medication (including doses): 20 items.
-Time since the last intake of EVOO.
-Time since the last intake of wine or other alcoholic beverages.

D. 11. 2 Blood pressure (BP) and anthropometric measurements

PREDIMED nurses, who are trained and certified for these measurements, measure BP, body weight and height. Several_quality control procedures are used to promote measurement accuracy. For BP measurements, participants rest quietly for five minutes in the seated position. A validated semi-automatic sphygmomanometer (Omron HEM-705CP) is used for the PREDIMED trial. An appropriate sized cuff is applied after measurement of arm circumference. A pulse obliteration pressure is obtained. At each visit, 3 measurements will be obtained, separated by 2 minutes. The average of second and third measurement is written in the data collection form. If both measurements differ more than 5 mmHg, the whole procedure is repeated and additional BP readings are averaged. Weight is measured using a calibrated balance beam scale with the subject barefoot and wearing light clothes. The nurse measures height using a wall-mounted calibrated stadiometer. Waist circumference is measured using an anthropometric measuring tape, at a horizontal plane midway between the lowest rib and the iliac crest.

D. 11. 3 Electrocardiograms (EKG)

At each yearly visit, the nurse obtains from the clinical record the last available EKG, and collects 2 copies. If no EKG has been taken during the last year, she communicates with the Primary Care physician to perform a new EKG and 2 copies are included in the PREDIMED file of the participant.

D. 11. 4 Extraction, processing and storage of biological samples

The PREDIMED nurses are directly responsible for collection, processing and storage of biological specimens. All PREDIMED nurses are experienced and registered nurses trained and certified to perform the specimen collection protocol. Training, before starting the trial, consisted of approximately 4 hours of theoretical information and 4 hours of practical instruction. Blood samples are collected at baseline and years 1, 3, 5 and 6 (or final visit) of follow-up according to the protocol shown in Table 2.

**Table 2. Blood samples taken at specified intervals.**

| | Number of tubes | Volume (ml) | |
|---|---|---|---|
| **Glass tube K3E EDTA** | **4** | **4.5** | **EDTA Plasma + buffy coat** |
| **Plastic tube K3E EDTA (cold)** | **1** | **3** | **Cold EDTA plasma (pre-refrigerated tube)** |
| **Glass tube 9NC Citrate** | **1** | **4.5** | **Citrate Plasma + buffy coat** |
| **Gel-Glass tube SST** | **2** | **4** | **Serum (one of them light-protected for Hcy)** |
| **Glass tube K3E EDTA*** | **1** | **5** | |
| **TOTAL** | **9** | **38.5** | |

*Only a random sample (10%) is analyzed for biomarkers of compliance: the remaining is stored for future analyses.

The plastic tube K3E EDTA (cold) and 1 gel-glass tube SST (for homocysteine) are refrigerated in ice prior to blood collection; after blood is collected, the tubes are kept cold in the ice container. Serum, citrate plasma and EDTA plasma samples are distributed in aliquots of 200 and 500 microliters, and stored at -80℃ for later analyses in the central laboratory. Biochemical measurements will be performed blindly and in the same batch for consecutive samples of each participant. Every FC has acquired a freezer with enough capacity to store these specimens. A urine sample is taken at the time of blood extraction, and 16 aliquots (650 microliters) are stored at -80ºC. Depending on the available funding for the PREDIMED trial, some of the blood samples could be omitted on years 2-4. All biological samples are processed at each FC not later than 2 hours after collection. During transport from the PCC to the FC laboratory, they are stored in a portable cooler (-4ºC). A clip of each toenail of each participant is stored at room temperature.

In addition, a complete blood count and routine biochemical measurements are performed yearly in the PCC (fasting blood glucose, uric acid, ALT, AST, gamma-glutamyl transpeptidase, alkaline phosphatases, bilirubin, creatinine, BUN; total, HDL-, and LDL-cholesterol; triglycerides, total protein, and albumin) together with a routine urine exam including the albumin/creatinine ratio in a recent sample. Genomic DNA has been isolated from leukocytes of all participants and some polymorphism in candidate genes that may modulate the cardiovascular response to diet (LIPC, LPL, PPARG, APOE, PON1, MTHFR, and others) are analyzed by PCR and further allelic discrimination. Some members of our team have played a relevant role in identifying gene-diet interactions, which can be of interest in our project (**Ordovas, 2001; Corella, 2002**).

D. 11.5 Outcome ascertainment

Outcomes will be determined by review by the **Clinical End Point Committee**. This panel will be blinded to the intervention group. The primary outcome is reported in section D3. The Clinical End Point Committee will ascertain cardiovascular deaths from clinical registers on the basis of the clinical records and a death certificates listing an International Classification of Diseases code corresponding to any cardiovascular death (CHD or

stroke). A definite non-fatal MI refers to a report of a clinical MI by a clinical center that meets the criteria for MI described in the Manual of Operations. Myocardial infarction is defined by the presence of symptoms suggestive of ischemia or infarction, with either EKG evidence (new Q waves in two or more leads) or cardiac-marker evidence of infarction, according to the standard American College of Cardiology definition **(Cannon, 2001).** Stroke diagnosis is based on rapid onset of a neurological deficit lasting more than 24 hours, supported by imaging studies (CT or MR scans). Secondary analyses will also be done.

**D. 12. Statistical Analysis**

The analyses will be based on the intention-to treat principle. With the actual sample size, it is assumed that we will have enough statistical power to evaluate the effects of the MeDiet (the two MeDiet groups considered as a single one) compared to the Low-fat Control diet on the primary prevention of cardiovascular diseases**.** We also might be able to assess differences in outcomes for the two MeDiets if the specific supplements of EVOO and mixed nuts prove to be differentially cardioprotective. The main variable for analysis will be time from randomization to event. For a given outcome, the time of the event will be defined as the number of days from randomization to the first post-randomization diagnosis, as determined by the adjudicator. For silent MI, the date of the follow-up EKG will be applied. Participants without a diagnosis will be censored at the time of last follow-up contact. The generally accepted method for analyzing this type of data is the log rank statistic. It has the advantage of requiring no assumptions other than the random assignment of the intervention. This analysis will be the primary measure of the success or failure of the trial. However, if baseline imbalances between groups are observed for any of the main CHD risk factors, a Cox proportional hazard analysis will be conducted stratified by FC, age, sex and adjusted for major risk factors. Primary outcome comparisons will be estimated as hazard ratios. Adjustment for other relevant variables (body mass index, physical activity, education level, marital status) also will be applied.

**D. 13 Monitoring Plan**

The Data and Knowledge Management Center (DKMC) will be responsible for preparing reports to monitor the progress of the study for the Steering Committee and the Data and Safety Monitoring Board (DSMB). These reports will focus on a) general progress of candidate recruitment and person-year of follow-up in comparison to pre-established targets; b) participant adherence (biochemical markers and FFQ data of compliance and comparison with targets for nutrients and food groups); c) quality control (digit preference, variability, outliers); d) clinical outcome data at each center and primary and secondary endpoints (group comparisons with respect to both the primary outcome, total mortality, and other secondary outcomes). In these sections, data will be provided for the study as a whole and, where appropriate, separately for each FC. Reports will be mailed to members of the DSMB two weeks prior to the meeting or to the date of review. Steps will be taken to insure security and confidentiality, including distribution by certified mail and enactment of a return policy of all reports. Comparison of groups with respect to major outcomes will be updated two days before the meeting so that the DSMB will have the most up-to-date data possible.

D.13.1. Quality of Data

To assess the reliability of the data included in the questionnaires and databases, external personnel will review medical records of a random sample of the participants. Data of around 25% of the participants included in each FC will be evaluated. Then DKMC and Data and Knowledge Management Center (DKMC) members will confirm these data (See later).

D.13.2. Data analysis

The data from the PREDIMED trial will be analyzed at 5 intervals (4 interim analyses and the final analysis). The 4 interim analyses to decide on the continuation of the PREDIMED trial will be conducted after 2, 3, 4 and 5 years of median follow-up. The 2-sided p-values for stopping the trial at each interim analysis (1st to 4th) are respectively $5*10^{-6}$, 0.001, 0.009 and 0.02 for benefit, and $9*10^{-5}$, 0.005, 0.02 and 0.05 for adverse effects, according to asymmetric O'Brien-Fleming boundaries **(O'Brien, 1979)**. The actual recommendation regarding stopping or continuing the trial will be made by the DSMB. There are 3 potential reasons for early ending of the trial: a) efficacy of MeDiet may be demonstrated; b) harmful effects of the intervention may be discovered; c) there may be no hope for a reasonable evaluation of the proposed hypothesis (i.e., if a small intervention differential exists, power may be seriously compromised and early termination of the trial may be considered). Early termination should be considered only if the intervention effect is really great.

## D. 14 Organizational structure

An overview of the organizational structure is displayed in http://www.predimed.org. The main funding for the trial comes from ISCIII (Spanish Government). A **Data and Safety Monitoring Board** (DSMB) supervises the trial. The Project Leader, Dr. Ramon Estruch (Hospital Clinic, University of Barcelona), is assisted by the **Steering Committee** to make strategic decisions along the project's life. The coordination, research and internal supervision activities of the project will be performed by the following three categories of units: coordination units, research and operative units, and subcommittees. Table 3 shows data of each unit. In summary, the research team includes 10 **Field Centers** (FC), three **Data Management and Statistical Units** (DMSU); a **Data and Knowledge Management Center** (DKMC); and 6 **Specialized Laboratory Units** (SLU). The **Steering Committee,** chaired by Dr. Estruch, includes the coordinators of each of the 11 FC, the investigator responsible of the each SLU and the Data Manager. There are also the following organizational structures: a **Diet Subcommittee**, co-chaired by Dr. Martinez-Gonzalez (Navarra) and Dr. Ros (Barcelona), a **Measurement Subcommittee**, chaired by Dr. Covas IMIM-Barcelona), a **Recruitment Subcommittee** (chaired by Dr. Salas), a **Clinical End Point Committee**, chaired by Dr. Arós (Cardiologist, Txagorritxu Hospital, Vitoria) and will include a clinician from each FC. The DSMB includes the following external advisors: Dr. Frank Hu, Harvard School of Public Health, Boston; Dr. F. Xavier Pi-Sunyer, Columbia University College of Physicians and Surgeons, New York; Dr. Joan Sabaté, Loma Linda University, CA; Dr. Carlos González, Principal Investigator of the Spanish branch of the EPIC Study, Barcelona. They will convene to review the implementation of the protocol and to monitor the trial's progress on an annual basis. In addition, as stated, a mailed report will be sent them periodically with the pertinent analyses to ascertain the continuation of the PREDIMED trial. Most of the Committees will convene by conference

calls and at in-person meetings (twice per year during years 1-4, once during year 5). The frequency of contacts will vary during the trial.

## D. 15 Data management

The DKMC will be co-directed by the Data Manager, Dr. Covas, and Dr. Martinez-Gonzalez. With the support of the DMSUs (IMIM, Navarra University & Hospital Clinic-Barcelona), they will develop and maintain the data base; and will provide statistical and trial monitoring support throughout the fieldwork. Data from screening, intervention and follow-up visits will be entered on specific forms at the FC, and sent monthly to the Data Manager in the DKMC, who will send reports of missing or inappropriate entries, for clarification and resolution, to the FC coordinators every month. The Data Manager will also provide monthly reports to Dr. Estruch on the quality and completeness of the data, organized by type of visit (screening visit 1, 1-year follow-up visit, etc.) and by specific data form. At the end of each yearly visit in each FC, the Data Manager will verify the completeness of data for each individual.

**Table 3. Description of the Project's Organic Units.**

| Categ. | Unit name | Responsibilities |
|---|---|---|
| Coordina-tion | Administration | General management, accounting, task schedule management. |
| | Technological Support | Technical and technological assistance in computing services, instrument installation and maintenance. |
| | Data and Knowledge Mana-gement Center (DKMC) | Statistical analysis, data management, report elaboration, database management, software support tools development, security of information and computer systems. |
| | Medical Support | Medical assistance in clinical tasks |
| Research and Operative | Barcelona University (Hospital Clinic) | FIELD CENTER (**FC)-1**: Patient recruitment, dietary behavioral intervention<br>**Specialized Laboratory Unit** (**SLU**)-1 |
| | Barcelona, IMIM***/** ICS(Primary Health Care Centers) | **FC-2**: Patient recruitment, dietary behavioral intervention<br>**Data Management and Statistical Unit** (**DMSU**)-1, **SLU-2** |
| | Navarra University | **FC-3** Patient recruitment, dietary behavioral intervention, **DMSU-2,** FFQ processing |
| | Valencia University | **FC-4**: Patient recruitment, dietary behavioral intervention, genetic analysis, **SLU-3** |
| | Malaga University | **FC-5**: Patient recruitment, dietary behavioral intervention, laboratory work |
| | Seville Primary Health Care | **FC-6**: Patient recruitment, dietary behavioral intervention |
| | Seville-CSIC* | **SLU-4** |
| | Mallorca, I. Balears University | **FC-7**:Patient recruitment, dietary behavioral intervention |
| | Tarragona, Rovira Virgili Univ. | **FC-8**: Patient recruitment, dietary behavioral intervention, laboratory work |
| | Vitoria, Txagorritxu Hospital | **FC-9**: Patient recruitment, dietary behavioral intervention |
| | Las Palmas, Gran Canaria Univ.. | **FC-10:** Patient recruitment, dietary behavioral intervention |
| | Bellvitge Hospital, Barcelona | **FC-11:** Patient recruitment, dietary behavioral intervention |
| | Zaragoza University | **SLU-5** |
| | Barcelona University – Pharmacy School | **SLU-6** |

*CSIC: Superior Council for Scientific Research. IMIM: Municipal Institute for Medical Research.

A private web-based system of data access has been created (www.predimed.org), where authorized investigators can download all the forms, datasets and published papers. For privacy and security, an ID and password are required to access the data and the forms. This web-based system is used to send data to the Data Manager. Quality control reports will be generated for key aspects of the trial, i.e., digit preference and variability. To reduce data entry expenses and speed processing, the questionnaires and data forms are optically scannable. The data forms will be entered in duplicate and missing data checks will be performed. After data entry, cross-form edit checks will also be performed. Data

inconsistencies will be checked. Audits will be rerun periodically to detect unsolved problems. Standardized edit reports that summarize problems in the database provide an additional method of assuring data quality. To minimize the potential for error, we have developed a detailed Manual of Operations. In addition we will conduct annual training meetings for staff. The DKMC will monitor the performance of each FC and will recommend new or corrective procedures in case deficiencies are noted. Until the end of the trial, all FC will be masked to trial outcome data, with the exception of the statisticians, the Data Manager and the external DSMB. Due to the nature of the intervention, however, dietitians and nurses at each FC need to be unmasked to diet assignment. The Clinical Event Subcommittee will be blinded to participants' allocation.

**D.16 Limitations of the study**

Fat consumption is customarily high in Mediterranean countries. Due to this fact, some participants have difficulty in following a low-fat diet on the long-term. We acknowledge this limitation because participants in the low-fat diet Control group belong to a Mediterranean culture and their food habits are derived from the fat-rich traditional MeDiet (**Serra-Majem, 2007**). We think that it would be unethical to strongly advice controls to get apart from this supposedly healthy diet. Instead, we are attempting to increase differences by using a comprehensive and expanded intervention in both experimental groups to attain further changes in their diet, thus maximizing the contrast.

The main differential point in our design is that we are evaluating two MeDiets that are higher in fat than all diets assessed by previous trials, but in both of them the main sources of fat are unsaturated fatty acids In addition, the effect of diets rich in polyphenols and phytosterols is also assessed. The rigorous methodology of the PREDIMED trial should convincingly answer what is certainly one of the major sources of controversy and unresolved issues regarding diet and cardiovascular risk, namely, the impact of replacing saturated fat with MUFA or with carbohydrate.

**D. 17. Dissemination of the results**

D.17.1 General strategies:

Exploitation of the scientific data obtained from the PREDIMED Study will be performed through a Dissemination Plan, which involves: i) Common definitions and data elements in order to homogenize scientific and common languages among investigators and general public; and ii) Implementation strategy, defined as the process through which people become aware of, assimilate, accept, adopt and use disseminated information will be used. Dissemination must involve more than the mere distribution of diffusion of information. Information of how to consume healthy food based on the cost/benefit ratio of food components to protect the interest of low-income people will be taken into account.

The dissemination framework must consider the audience, the types of strategies used to foster assimilation, and adoption of information, and the various media involved. Potential audiences will be divided into six major categories: I) consumers; ii) nutritionists and clinical practitioners; iii) food industry; iv) policy makers; v) researchers; and vi) journalists. Strategies will be divided into two general categories: i) nutritionists, clinical practitioners and researchers (conferences, workshops, continuous professional education, institution web sites, and technical journals; ii) target consumers through the support of the food industry and international consumer organizations (i.e. possible contact

with the American Consumers Organization to be in charge of specific dissemination tools) to promote dissemination through the printed media, radio and TV.

D. 17.2. Specific strategies:

- Two Web pages for the trial:
    - http: www.predimed.org
    - http: www.predimed.es

  are available to obtain the details of the trial, including intervention tools. They will be of public use except for specific sections, such as database, until study completion.
- The final results of the study (primary and secondary end-points) and analysis of surrogate variables at different intervals of the study will be published in top medical journals.
- An International Meeting will be organized to present the final results of the study. They will include lectures on the main aspects of nutrition and cardiovascular disease.
- A monograph and a CD-rom covering the main aspects of the study will be edited and sent to each participant, to scientific societies related to Internal Medicine, Cardiology, Oncology, Cardiovascular Epidemiology and Nutrition, and to political authorities related to public health, nutrition, and agriculture.

## E. LITERATURE CITED

Albert CM, Gaziano JM, Willett WC, Manson JE. Nut consumption and decreased risk of sudden cardiac death in the Physicians' Health Study. Arch Intern Med. 2002;162:1382-7.

Ascherio A, Rimm EB, Giovannucci EL, Spiegelman D, Stampfer M, Willett WC. Dietary fat and risk of coronary heart disease in men: cohort follow up study in the United States. BMJ. 1996;313:84-90.

Baylin A, Kabagambe EK, Ascherio A, Spiegelman D, Campos H. Adipose tissue alpha-linolenic acid and nonfatal acute myocardial infarction in Costa Rica. Circulation. 2003;107:1586-91.

Becker A, van Hinsbergh VW, Jager A, Kostense PJ, Dekker JM, Nijpels G, Heine RJ, Bouter LM, Stehouwer CD. Why is soluble intercellular adhesion molecule-1 related to cardiovascular mortality? Eur J Clin Invest. 2002;32:1-8.

Bondia-Pons I, Serra-Majem L, Castellote AI, López-Sabater MC. Identification of foods contributing to the dietary lipid profile of a Mediterranean population. Br J Nutr. 2007;98:583-92.

Bray GA, Popkin BM. Dietary fat intake does affect obesity! Am J Clin Nutr. 1998;68:1157-73.

Brouwer IA, Katan MB, Zock PL. Dietary alpha-linolenic acid is associated with reduced risk of fatal coronary heart disease, but increased prostate cancer risk: a meta-analysis. J Nutr. 2004;134:919-22.

Canga N, De Irala J, Vara E, Duaso MJ, Ferrer A, Martinez-Gonzalez MA. Intervention study for smoking cessation in diabetic patients: a randomized controlled trial in both clinical and primary care settings. Diabetes Care. 2000;23:1455-60.

Cannon CP, Battler A, Brindis RG, Cox JL, Ellis SG, Every NR, Flaherty JT, Harrington RA, Krumholz HM, Simoons ML, Van De Werf FJ, Weintraub WS, Mitchell KR, Morrisson SL, Brindis RG, Anderson HV, Cannom DS, Chitwood WR, Cigarroa JE, Collins-Nakai RL, Ellis SG, Gibbons RJ, Grover FL, Heidenreich PA, Khandheria BK, Knoebel SB, Krumholz HL, Malenka DJ, Mark DB, Mckay CR, Passamani ER, Radford MJ, Riner RN, Schwartz JB, Shaw RE, Shemin RJ, Van Fossen DB, Verrier ED, Watkins MW, Phoubandith DR, Furnelli T. American College of Cardiology key data elements and definitions for measuring the clinical management and outcomes of patients with acute coronary syndromes. A report of the American College of Cardiology Task Force on Clinical Data Standards (Acute Coronary Syndromes Writing Committee). J Am Coll Cardiol. 2001;38:2114-30.

Carluccio MA, Massaro M, Bonfrate C, Siculella L, Maffia M, Nicolardi G, Distante A, Storelli C, De Caterina R. Oleic acid inhibits endothelial activation: A direct vascular antiatherogenic mechanism of a nutritional component in the mediterranean diet. Arterioscler Thromb Vasc Biol. 1999;19:220-8.

Carluccio MA, Siculella L, Ancora MA, Massaro M, Scoditti E, Storelli C, Visioli F, Distante A, De Caterina R. Olive oil and red wine antioxidant polyphenols inhibit

endothelial activation: antiatherogenic properties of Mediterranean diet phytochemicals. Arterioscler Thromb Vasc Biol. 2003;23:622-9.

Connor WE, Connor SL. Should a low-fat, high-carbohydrate diet be recommended for everyone? The case for a low-fat, high-carbohydrate diet. N Engl J Med. 1997;337:562-3.

Corella D, Guillen M, Saiz C, Portoles O, Sabater A, Folch J, Ordovas JM. Associations of LPL and APOC3 gene polymorphisms on plasma lipids in a Mediterranean population: interaction with tobacco smoking and the APOE locus. J Lipid Res. 2002;43:416-27.

Cortés B, Núñez I, Cofán M, Gilabert R, Pérez-Heras A, Casals E, Deulofeu R, Ros E. Acute effects of high-fat meals enriched with walnuts or olive oil on postprandial endothelial function in healthy subjects and patients with hypercholesterolemia. J Am Coll Cardiol. 2006;48:1666-71.

Costacou T, Bamia C, Ferrari P, Riboli E, Trichopoulos D, Trichopoulou A. Tracing the Mediterranean diet through principal components and cluster analyses in the Greek population. Eur J Clin Nutr. 2003;57:1378-85.

Covas MI, Nyyssönen K, Poulsen HE, Kaikkonen J, Zunft HJ, Kiesewetter H, Gaddi A, de la Torre R, Mursu J, Bäumler H, Nascetti S, Salonen JT, Fitó M, Virtanen J, Marrugat J, EUROLIVE Study Group. The effect of polyphenols in olive oil on heart disease risk factors: a randomized trial. Ann Intern Med. 20065;145:333-41.

de la Puerta R, Ruiz Gutierrez V, Hoult JR. Inhibition of leukocyte 5-lipoxygenase by phenolics from virgin olive oil. Biochem Pharmacol. 1999;57:445-9.

de la Puerta R, Martinez Dominguez ME, Ruiz-Gutierrez V, Flavill JA, Hoult JR. Effects of virgin olive oil phenolics on scavenging of reactive nitrogen species and upon nitrergic neurotransmission. Life Sci. 2001;69:1213-22.

de Lorgeril M, Salen P, Martin JL, Monjaud I, Delaye J, Mamelle N. Mediterranean diet, traditional risk factors, and the rate of cardiovascular complications after myocardial infarction: final report of the Lyon Diet Heart Study. Circulation. 1999;99:779-85.

Desideri G, Croce G, Marinucci MC, Masci PG, Stati M, Valeri L, Santucci A, Ferri C. Prolonged, low dose alpha-tocopherol therapy counteracts intercellular cell adhesion molecule-1 activation. Clin Chim Acta. 2002;320:5-9.

Djousse L, Folsom AR, Province MA, Hunt SC, Ellison RC; National Heart, Lung, and Blood Institute Family Heart Study. Dietary linolenic acid and carotid atherosclerosis: the National Heart, Lung, and Blood Institute Family Heart Study. Am J Clin Nutr. 2003;77:819-25.

Ellsworth JL, Kushi LH, Folsom AR. Frequent nut intake and risk of death from coronary heart disease and all causes in postmenopausal women: the Iowa Women's Health Study. Nutr Metab Cardiovasc Dis. 2001;11:372-7.

Elosua R, Garcia M, Aguilar A, Molina L, Covas MI, Marrugat J. Validation of the Minnesota Leisure Time Physical Activity Questionnaire In Spanish Women. Investigators of the MARATDON Group. Med Sci Sports Exerc. 2000;32:1431-7.

Elosua R, Marrugat J, Molina L, Pons S, Pujol E. Validation of the Minnesota Leisure Time Physical Activity Questionnaire in Spanish men. The MARATHOM Investigators. Am J Epidemiol. 1994;139:1197-209.

Estruch R, Martínez-González MA, Corella D, Salas-Salvadó J, Ruiz-Gutiérrez V, Covas MI, Fiol M, Gómez-Gracia E, López-Sabater MC, Vinyoles E, Arós F, Conde M, Lahoz C, Lapetra J, Sáez G, Ros E on behalf of the PREDIMED Study Investigators. Effects of a Mediterranean-style diet on cardiovascular risk factors. A randomized trial. Ann Intern Med 2006;145:1-11.

Esposito K, Marfella R, Ciotola M, Di Palo C, Giuglano F, Guiglano G, D'Armiento M, D'Andrea F, Guiglano D. Effect of a Mediterranean-style diet on endothelial dysfunction and markers of vascular inflammation in the metabolic syndrome: a randomised trial. JAMA 2004; 292: 1440 – 6.

Feldman EB. The scientific evidence for a beneficial health relationship between walnuts and coronary heart disease. J Nutr. 2002;132:1062S-1101S.

Ferrara LA, Raimondi AS, d'Episcopo L, Guida L, Dello Russo A, Marotta T. Olive oil and reduced need for antihypertensive medications. Arch Intern Med. 2000 27;160:837-42.

Ferro-Luzzi A, James WP, Kafatos A. The high-fat Greek diet: a recipe for all? Eur J Clin Nutr. 2002;56:796-809.

Fitó M, Guxens M, Corella D, Sáez G, Estruch R, de la Torre R, Francés F, Cabezas C, López-Sabater MC, Marrugat J, García-Arellano A, Arós F, Ruiz-Gutierrez V, Ros E, Salas-Salvadó J, Fiol M, Solá R, Covas MI, on behalf of the PREDIMED Study Investigators. Effect of a traditional Mediterranean diet on lipoprotein oxidation. A randomized, controlled trial. Arch Intern Med 2007;167:1195-1203.

Fraser GE, Sabate J, Beeson WL, Strahan TM. A possible protective effect of nut consumption on risk of coronary heart disease. The Adventist Health Study. Arch Intern Med. 1992;152:1416-24.

Fuentes F, Lopez-Miranda J, Sanchez E, Sanchez F, Paez J, Paz-Rojas E, Marin C, Gomez P, Jimenez-Pereperez J, Ordovas JM, Perez-Jimenez F. Mediterranean and low-fat diets improve endothelial function in hypercholesterolemic men. Ann Intern Med. 2001;134:1115-9.

Fung TT, Rexrode KM, Mantzoros CS, Manson JE, Willett WC, Hu FB. Mediterranean diet and incidence of and mortality from coronary heart disease and stroke in women. Circulation. 2009;119:1093-100.

Gabriel R, Alonso M, Segura A, et al on behalf of the ERICE Cooperative Group. Prevalence, geographic distribution, and geographic variability of major cardiovascular risk factors in Spain. Pooled analysis of data from population-based epidemiological studies: the ERICE Study. Rev Esp Cardiol 2008;61:1030-40.

Garcia-Lorda P, Megias Rangil I, Salas-Salvado J. Nut consumption, body weight and insulin resistance. Eur J Clin Nutr. 2003;57 Suppl 1:S8-11.

Guallar E, Aro A, Jimenez FJ, Martin-Moreno JM, Salminen I, van't Veer P, Kardinaal AF, Gomez-Aracena J, Martin BC, Kohlmeier L, Kark JD, Mazaev VP, Ringstad J, Guillen J, Riemersma RA, Huttunen JK, Thamm M, Kok FJ. Omega-3 fatty acids in adipose tissue and risk of myocardial infarction: the EURAMIC study. Arterioscler Thromb Vasc Biol. 1999;19:1111-8.

Haim M, Tanne D, Boyko V, Reshef T, Goldbourt U, Leor J, Mekori YA, Behar S. Soluble intercellular adhesion molecule-1 and long-term risk of acute coronary events in patients with chronic coronary heart disease. Data from the Bezafibrate Infarction Prevention (BIP) Study. J Am Coll Cardiol. 2002;39:1133-8.

Hu FB, Stampfer MJ, Manson JE, Rimm EB, Colditz GA, Rosner BA, Speizer FE, Hennekens CH, Willett WC. Frequent nut consumption and risk of coronary heart disease in women: prospective cohort study. BMJ. 1998;317:1341-5.

Hu FB, Stampfer MJ, Manson JE, Rimm EB, Wolk A, Colditz GA, Hennekens CH, Willett WC. Dietary intake of alpha-linolenic acid and risk of fatal ischemic heart disease among women. Am J Clin Nutr. 1999;69:890-7.

Hu FB. Dietary pattern analysis: a new direction in nutritional epidemiology. Curr Opin Lipidol 2002;13:3-9.

Hu FB. The Mediterranean diet and mortality--olive oil and beyond.N Engl J Med. 2003;348:2595-6.

Hwang SJ, Ballantyne CM, Sharrett AR, Smith LC, Davis CE, Gotto AM Jr, Boerwinkle E. Circulating adhesion molecules VCAM-1, ICAM-1, and E-selectin in carotid atherosclerosis and incident coronary heart disease cases: the Atherosclerosis Risk In Communities (ARIC) study. Circulation. 1997;96:4219-25.

Jacobs DR Jr, Steffen LM. Nutrients, foods, and dietary patterns as exposures in research: a framework for food synergy. Am J Clin Nutr. 2003;78(3 Suppl):508S-513S.

Jacques PF, Tucker KL. Are dietary patterns useful for understanding the role of diet in chronic disease? Am J Clin Nutr. 2001;73:1-2.

Jequier E, Bray GA. Low-fat diets are preferred. Am J Med. 2002;113 Suppl 9B:41S-46S.

Joint WHO/FAO Expert Consultation on Diet, Nutrition and the Prevention of Chronic Diseases (2002: Geneva, Switzerland). WHO technical report series; 916. Geneva: WHO, 2003.

Kew S, Mesa MD, Tricon S, Buckley R, Minihane AM, Yaqoob P. Effects of oils rich in eicosapentaenoic and docosahexaenoic acids on immune cell composition and function in healthy humans. Am J Clin Nutr. 2004;79:674-81.

Kouris-Blazos A, Gnardellis C, Wahlqvist ML, Trichopoulos D, Lukito W, Trichopoulou A. Are the advantages of the Mediterranean diet transferable to other populations? A cohort study in Melbourne, Australia. Br J Nutr. 1999;82:57-61.

Kris-Etherton P, Eckel RH, Howard BV, St Jeor S, Bazzarre TL; Nutrition Committee Population Science Committee and Clinical Science Committee of the American

Heart Association. AHA Science Advisory: Lyon Diet Heart Study. Benefits of a Mediterranean-style, National Cholesterol Education Program/American Heart Association Step I Dietary Pattern on Cardiovascular Disease. Circulation. 2001;103:1823-5.

Kris-Etherton PM, Zhao G, Binkoski AE, Coval SM, Etherton TD. The effects of nuts on coronary heart disease risk. Nutr Rev. 2001;59:103-11.

Lasheras C, Fernandez S, Patterson AM. Mediterranean diet and age with respect to overall survival in institutionalized, nonsmoking elderly people. Am J Clin Nutr. 2000;71:987-92.

Lercker G Lercker G, Rodriguez-Estrada MT. Chromatographic analysis of unsaponifiable compounds of olive oils and fat-containing foods. J Chromatogr A. 2000;881:105-29.

Lopez-Azpiazu I, Martinez-Gonzalez MA, Leon-Mateos A, Kearney J, Gibney M, Martinez JA. Stages of dietary change and nutrition attitudes in the Spanish population. Public Health. 2000;114:183-9.

Lopez-Garcia E, Schulze MB, Manson JE, Meigs JB, Albert CM, Rifai N, Willett WC, Hu FB. Consumption of (n-3) fatty acids is related to plasma biomarkers of inflammation and endothelial activation in women. J Nutr. 2004;134:1806-11.

Luc G, Arveiler D, Evans A, Amouyel P, Ferrieres J, Bard JM, Elkhalil L, Fruchart JC, Ducimetiere P; PRIME Study Group. Circulating soluble adhesion molecules ICAM-1 and VCAM-1 and incident coronary heart disease: the PRIME Study. Atherosclerosis. 2003;170:169-76.

Machowetz A, Poulsen HE, Gruendel S, Weimann A, Fitó M, Marrugat J, de la Torre R, Salonen JT, Nyyssönen K, Mursu J, Nascetti S, Gaddi A, Kiesewetter H, Bäumler H, Selmi H, Kaikkonen J, Zunft HJ, Covas MI, Koebnick C. Effect of olive oils on biomarkers of oxidative DNA stress in Northern and Southern Europeans. FASEB J. 2007;21:45-52.

Malik I, Danesh J, Whincup P, Bhatia V, Papacosta O, Walker M, Lennon L, Thomson A, Haskard D. Soluble adhesion molecules and prediction of coronary heart disease: a prospective study and meta-analysis. Lancet. 2001;358:971-6.

Manna C, D'Angelo S, Migliardi V, Loffredi E, Mazzoni O, Morrica P, Galletti P, Zappia V. Protective effect of the phenolic fraction from virgin olive oils against oxidative stress in human cells. J Agric Food Chem. 2002;50:6521-6.

Mangiapane EH, McAteer MA, Benson GM, White DA, Salter AM. Modulation of the regression of atherosclerosis in the hamster by dietary lipids: comparison of coconut oil and olive oil. Br J Nutr. 1999;82:401-9.

Martinez-Gonzalez MA, Fernandez-Jarne E, Serrano-Martinez M, Marti A, Martinez JA, Martin-Moreno JM. Mediterranean diet and reduction in the risk of a first acute myocardial infarction: an operational healthy dietary score. Eur J Nutr. 2002;41:153-60.

Martinez-Gonzalez MA, Estruch R. Mediterranean diet, antioxidants and cancer: the need for randomized trials. Eur J Cancer Prev. 2004;13:327-35.

Martinez-Gonzalez MA, Sanchez-Villegas A. The emerging role of Mediterranean diets in cardiovascular epidemiology: monounsaturated fats, olive oil, red wine or the whole pattern? Eur J Epidemiol. 2004;19(1):9-13.

Martinez-Gonzalez MA, Fernandez-Jarne E, Serrano-Martinez M, Wright M, Gomez-Gracia E. Development of a short dietary intake questionnaire for the quantitative estimation of adherence to a cardioprotective Mediterranean diet. Eur J Clin Nutr. 2004;58:1550-2.

Martin-Moreno JM, Boyle P, Gorgojo L, Maisonneuve P, Fernandez-Rodriguez JC, Salvini S, Willett WC. Development and validation of a food frequency questionnaire in Spain. Int J Epidemiol. 1993;22:512-9.

Marrugat J, Covas MI, Fito M, Schroder H, Miro-Casas E, Gimeno E, Lopez-Sabater MC, de la Torre R, Farre M; SOLOS Investigators. Effects of differing phenolic content in dietary olive oils on lipids and LDL oxidation--a randomized controlled trial. Eur J Nutr. 2004;43:140-7.

Mata P, Varela O, Alonso R, Lahoz C, de Oya M, Badimon L. Monounsaturated and polyunsaturated n-6 fatty acid-enriched diets modify LDL oxidation and decrease human coronary smooth muscle cell DNA synthesis. Arterioscler Thromb Vasc Biol. 1997;17:2088-95.

Mataix J, Quiles JL, Huertas JR, Battino M, Manas M. Tissue specific interactions of exercise, dietary fatty acids, and vitamin E in lipid peroxidation. Free Radic Biol Med. 1998;24:511-21.

McManus K, Antinoro L, Sacks F. A randomized controlled trial of a moderate-fat, low-energy diet compared with a low fat, low-energy diet for weight loss in overweight adults. Int J Obes Relat Metab Disord. 2001;25:1503-11.

Meigs JB, Hu FB, Rifai N, Manson JE. Biomarkers of endothelial dysfunction and risk of type 2 diabetes mellitus. JAMA. 2004;291:1978-86.

Miles EA, Thies F, Wallace FA, Powell JR, Hurst TL, Newsholme EA, Calder PC. Influence of age and dietary fish oil on plasma soluble adhesion molecule concentrations. Clin Sci (Lond). 2001;100:91-100.

Mitrou PN, Kipnis V, Thiébaut AC, Reedy J, Subar AF, Wirfält E, Flood A, Mouw T, Hollenbeck AR, Leitzmann MF, Schatzkin A. Mediterranean dietary pattern and prediction of all-cause mortality in a US population: results from the NIH-AARP Diet and Health Study. Arch Intern Med.2007;167:2461-8.

Mossavar-Rahmani Y, Henry H, Rodabough R, Bragg C, Brewer A, Freed T, Kinzel L, Pedersen M, Soule CO, Vosburg S. Additional self-monitoring tools in the dietary modification component of The Women's Health Initiative. J Am Diet Assoc. 2004;104:76-85.

Nigg CR, Burbank PM, Padula C, Dufresne R, Rossi JS, Velicer WF, Laforge RG, Prochaska JO. Stages of change across ten health risk behaviors for older adults. Gerontologist. 1999;39:473-82.

O'Brien PC, Fleming TR. A multiple testing procedure for clinical trials. Biometrics. 1979;35:549-56.

Oomen CM, Ocke MC, Feskens EJ, Kok FJ, Kromhout D. alpha-Linolenic acid intake is not beneficially associated with 10-y risk of coronary artery disease incidence: the Zutphen Elderly Study. Am J Clin Nutr. 2001;74:457-63.

Ordovas JM, Corella D, Demissie S, Cupples LA, Couture P, Coltell O, Wilson PW, Schaefer EJ, Tucker KL. Dietary fat intake determines the effect of a common polymorphism in the hepatic lipase gene promoter on high-density lipoprotein metabolism: evidence of a strong dose effect in this gene-nutrient interaction in the Framingham Study. Circulation. 2002;106:2315-21.

Panagiotakos DB, Pitsavos C, Chrysohoou C, Stefanadis C, Toutouzas P. Risk stratification of coronary heart disease in Greece: final results from the CARDIO2000 Epidemiological Study. Prev Med. 2002;35:548-56.

Patterson RE, Kristal A, Rodabough R, Caan B, Lillington L, Mossavar-Rahmani Y, Simon MS, Snetselaar L, Van Horn L. Changes in food sources of dietary fat in response to an intensive low-fat dietary intervention: early results from the Women's Health Initiative. J Am Diet Assoc. 2003;103:454-60.

Perez-Jimenez F, Lopez-Miranda J, Mata P. Protective effect of dietary monounsaturated fat on arteriosclerosis: beyond cholesterol. Atherosclerosis. 2002;163:385-98.

Perez-Jimenez F, Alvarez de Cienfuegos G, Badimon L, Barja G, Battino M, Blanco A, Bonanome A, Colomer R, Corella-Piquer D, Covas I, Chamorro-Quiros J, Escrich E, Gaforio JJ, Garcia Luna PP, Hidalgo L, Kafatos A, Kris-Etherton PM, Lairon D, Lamuela-Raventos R, Lopez-Miranda J, Lopez-Segura F, Martinez-Gonzalez MA, Mata P, Mataix J, Ordovas J, Osada J, Pacheco-Reyes R, Perucho M, Pineda-Priego M, Quiles JL, Ramirez-Tortosa MC, Ruiz-Gutierrez V, Sanchez-Rovira P, Solfrizzi V, Soriguer-Escofet F, de la Torre-Fornell R, Trichopoulos A, Villalba-Montoro JM, Villar-Ortiz JR, Visioli F. International conference on the healthy effect of virgin olive oil. Eur J Clin Invest 2005;35:421-4.

Pradhan AD, Manson JE, Rossouw JE, Siscovick DS, Mouton CP, Rifai N, Wallace RB, Jackson RD, Pettinger MB, Ridker PM. Inflammatory biomarkers, hormone replacement therapy, and incident coronary heart disease: prospective analysis from the Women's Health Initiative observational study. JAMA. 2002;288:980-7.

Puerta Vazquez R, Martinez-Dominguez E, Sanchez Perona J, Ruiz-Gutierrez V. Effects of different dietary oils on inflammatory mediator generation and fatty acid composition in rat neutrophils. Metabolism. 2004;53:59-65.

Rallidis LS, Paschos G, Papaioannou ML, Liakos GK, Panagiotakos DB, Anastasiadis G, Zampelas A. The effect of diet enriched with alpha-linolenic acid on soluble

cellular adhesion molecules in dyslipidaemic patients. Atherosclerosis. 2004;174:127-32.

Ramirez-Tortosa MC, Urbano G, Lopez-Jurado M, Nestares T, Gomez MC, Mir A, Ros E, Mataix J, Gil A. Extra-virgin olive oil increases the resistance of LDL to oxidation more than refined olive oil in free-living men with peripheral vascular disease. J Nutr. 1999;129:2177-83.

Reddy KS. Cardiovascular disease in non-Western countries. N Engl J Med. 2004;350:2438-40.

Ridker PM, Hennekens CH, Buring JE, Rifai N. C-reactive protein and other markers of inflammation in the prediction of cardiovascular disease in women. N Engl J Med. 2000;342:836-43.

Ridker PM, Rifai N, Stampfer MJ, Hennekens CH. Plasma concentration of interleukin-6 and the risk of future myocardial infarction among apparently healthy men. Circulation. 2000;101:1767-72.

Ros E. Dietary cis-monounsaturated fatty acids and metabolic control in type 2 diabetes. Am J Clin Nutr. 2003;78(3 Suppl):617S-625S.

Ros E, Nunez I, Perez-Heras A, Serra M, Gilabert R, Casals E, Deulofeu R. A walnut diet improves endothelial function in hypercholesterolemic subjects: a randomized crossover trial. Circulation. 2004;109:1609-14.

Sabate J. Nut consumption and body weight. Am J Clin Nutr. 2003;78(3 Suppl):647S-650S.

Sanchez-Villegas A, Delgado-Rodriguez M, Martinez-Gonzalez MA, De Irala-Estevez J; Seguimiento Universidad de Navarra group. Gender, age, socio-demographic and lifestyle factors associated with major dietary patterns in the Spanish Project SUN (Seguimiento Universidad de Navarra). Eur J Clin Nutr. 2003;57:285-92.

Serra-Majem L, Ribas-Barba L, Salvador G, Jover L, Raidó B, Ngo J, Plasencia A. Trends in energy and nutrient intake and risk of inadequate intakes in Catalonia, Spain (1992-2003). Public Health Nutr. 2007;10(11A):1354-67.

Shai I, Schwarzfuchs D, Henkin Y, Shadar DR, Witkow S, Greenberg I, Golan R, Fraser D, Bolotin A, Vardi H, Tangi-Rozental O, Zuk-Ramot R, Sarusi B, Brickner D, Schwartz Z, Sheiner E, Marko R, Katorza E, Thiery J, Fiedler GM, Blüher M, Stumvoll M, Stampfer MJ, Dietary Intervention Randomized Controlled Trial (DIRECT) Group. Weight loss with low-carbohydrate, Mediterranean and low-fat diet. N Engl J Med. 2008;359:229-41.

Sofi F, Cesari F, Abbate R, Gensini GF, Casini A. Adherence to Mediterranean diet and health status: meta-analysis. 2008 Sep 11;337:a1344. doi: 10.1136/bmj.a1344

Tanne D, Haim M, Boyko V, Goldbourt U, Reshef T, Matetzky S, Adler Y, Mekori YA, Behar S. Soluble intercellular adhesion molecule-1 and risk of future ischemic stroke: a nested case-control study from the Bezafibrate Infarction Prevention (BIP) study cohort. Stroke. 2002;33:2182-6.

Trichopoulou A, Kouris-Blazos A, Wahlqvist ML, Gnardellis C, Lagiou P, Polychronopoulos E, Vassilakou T, Lipworth L, Trichopoulos D. Diet and overall survival in elderly people. BMJ. 1995;311:1457-60.

Trichopoulou A, Costacou T, Bamia C, Trichopoulos D. Adherence to a Mediterranean diet and survival in a Greek population. N Engl J Med. 2003;348:2599-608.

Tunstall-Pedoe H, Kuulasmaa K, Mahonen M, Tolonen H, Ruokokoski E, Amouyel P. Contribution of trends in survival and coronary-event rates to changes in coronary heart disease mortality: 10-year results from 37 WHO MONICA project populations. Monitoring trends and determinants in cardiovascular disease. Lancet. 1999 May 8;353(9164):1547-57.

USDA (2000). Nutrition and Your Health: Dietary Guidelines for Americans. Washington: US Department of Agriculture. http://www.health.gov/ dietaryguidelines/ dga2000/ document/ contents.htm.

Visioli F, Galli C. The effect of minor constituents of olive oil on cardiovascular disease: new findings. Nutr Rev. 1998;56:142-7.

Visioli F, Galli C. Biological properties of olive oil phytochemicals. Crit Rev Food Sci Nutr. 2002;42:209-21.

Visioli F, Caruso D, Grande S, Bosisio R, Villa M, Galli G, Sirtori C, Galli C. Virgin Olive Oil Study (VOLOS): vasoprotective potential of extra virgin olive oil in mildly dyslipidemic patients. Eur J Nutr. 2004;1-7 [Epub ahead of print]

Vissers MN, Zock PL, Katan MB. Bioavailability and antioxidant effects of olive oil phenols in humans: a review. Eur J Clin Nutr 2004 ;58: 955–965.

Willett WC, Sacks F, Trichopoulou A, Drescher G, Ferro-Luzzi A, Helsing E, Trichopoulos D. Mediterranean diet pyramid: a cultural model for healthy eating. Am J Clin Nutr. 1995;61(6 Suppl):1402S-1406S.

Willett WC. Will high-carbohydrate/low-fat diets reduce the risk of coronary heart disease? Proc Soc Exp Biol Med. 2000;225:187-90.

World Health Organization. Health for All: statistical database. Geneva: WHO, Regional Office for Europe, 1993.

World Health Organization (2003a). Neglected Global Epidemics: three growing threats. In: WHO: The world health report 2003 - shaping the future. Geneva: WHO, 2003.

World Health Organization (2003b). Joint WHO/FAO Expert Consultation on Diet, Nutrition and the Prevention of Chronic Diseases. WHO technical report series; 916. Geneva: WHO, 2003.

Yaqoob P, Knapper JA, Webb DH, Williams CM, Newsholme EA, Calder PC. Effect of olive oil on immune function in middle-aged men. Am J Clin Nutr. 1998;67:129-35.

Zazpe I, Sanchez-Tainta A, Estruch R, Lamuela-Raventos RM, Schröder H, Salas-Salvado J, Corella D, Fiol M, Gomez-Gracia E, Aros F, Ros E, Ruiz-Gutierrez V, Iglesias P, Conde-Herrera M, Martinez-Gonzalez MA. A large randomized individual and group intervention conducted by dieticians increased the adherence to

Mediterranean-type diets: The PREDIMED study. J Am Diet Assoc. 2008;108:1134-44.

**CONSULTANTS**

1. Hu, Frank B, MD, PhD. Professor of Nutrition and Epidemiology. Harvard School of Public Health, Director, Boston Obesity and Nutrition Research Center, Epidemiology Core, Boston, Massachusetts.
2. Ordovas, Jose, PhD. Director, Nutrition and Genomics Laboaratory. Professor of Nutrition and Genetics, Jean Mayer United States Department of Agriculture. Human Nutrition Research Center on Aging at Tufts University, Boston, Massachusetts.

**Table 4. Quantitative Score of Compliance with the Mediterranean Diet**

| | Foods and frequency of consumption | Criteria for 1 point* |
|---|---|---|
| 1 | Do you use olive oil as main culinary fat? | Yes |
| 2 | How much olive oil do you consume in a given day (including oil used for frying, salads, out of house meals, etc.)? | 4 or more tablespoons |
| 3 | How many vegetable servings do you consume per day? *(1 serving = 200g - consider side dishes as 1/2 serving)* | 2 or more *(at least 1 portion raw or as salad)* |
| 4 | How many fruit units (including natural fruit juices) do you consume per day? | 3 or more |
| 5 | How many servings of red meat, hamburger, or meat products (ham, sausage, etc.) do you consume per day? *(1 serving = 100-150 g)* | Less than 1 |
| 6 | How many servings of butter, margarine, or cream do you consume per day? *(1 serving = 12 g)* | Less than 1 |
| 7 | How many sweet/carbonated beverages do you drink per day? | Less than 1 |
| 8 | How much wine do you drink per week? | 7 or more glasses |
| 9 | How many servings of legumes do you consume per week? *(1 serving = 150 g)* | 3 or more |
| 10 | How many servings of fish or shellfish do you consume per week? (1 serving: 100-150 g fish, or 4-5 units or 200 g shellfish) | 3 or more |
| 11 | How many times per week do you consume commercial sweets or pastries (not homemade), such as cakes, cookies, biscuits, or custard? | Less than 3 |
| 12 | How many servings of nuts (including peanuts) do you consume per week? *(1 serving = 30 g)* | 3 or more |
| 13 | Do you preferentially consume chicken, turkey o rabbit meat instead of veal, pork, hamburger or sausage? | Yes |
| 14 | How many times per week do you consume vegetables, pasta, rice, or other dishes seasoned with *sofrito* (sauce made with tomato and onion, leek, or garlic, simmered with olive oil)? | 2 or more |

* 0 points if these criteria are not met.

**Table 5. Quantitative Score of Compliance with the Control (Low-Fat) Diet.**

| | **Foods and frequency of consumption** | **Criteria for 1 point*** |
|---|---|---|
| 1 | How much olive oil do you consume in a given day (including oil used for frying, salads, out of house meals, etc.)? | 2 or less tablespoons<br>*(1 tablespoon=10 ml)* |
| 2 | Do you remove visible fat (or the skin) of chicken, duck, pork, lamb or veal meats before cooking and the fat of soups, broths, and cooked meat dishes before consumption? | Yes |
| 3 | How many servings of fat-rich meats, hamburger, commercial ground meat, sausage, cold meat, cured ham, bacon, salami, or offal do you consume per week? (meat serving: 100 g; salami or bacon: 30 g) | 1 or less |
| 4 | How many servings of butter, margarine, lard, mayonnaise, milk cream, or milk-based ice cream do you consume per week? (spread fat: serving: 12 g; ice cream: 100 g) | 1 or less |
| 5 | Do you exclusively consume low-fat dairy products? | Yes<br>*(id. If no dairy consumption)* |
| 6 | How many times per week do you prepare rice, pasta, potato, or legume dishes by using “sofrito” sauce (based on olive oil), bacon, salami, or fatty meats such as pork or lamb ribs? | 2 or less |
| 7 | How many times per week do you consume fatty fish or fish or seafood canned in oil? | 1 or less |
| 8 | How many servings of commercial sweets or industrial bakery products (not homemade), such as cakes, cookies, biscuits, or custard do you consume per week? (cake serving: 80 g; 6 biscuits: 40 g) | 1 or less |
| 9 | How many times per week do you consume nuts (including peanuts), potato chips, French fries, or commercial snacks? | 1 or less |

* 0 points if these criteria are not met.
